# Single twistable tendon-driven continuum robots

**DOI:** 10.1038/s41467-026-74225-3

**Published:** 2026-07-04

**Authors:** Jiewen Lai, Yanjun Liu, Tian-Ao Ren, Yan Ma, Tao Zhang, Jeremy Yuen-Chun Teoh, Mark R. Cutkosky, Hongliang Ren

**Affiliations:** 1 Department of Electronic Engineering, CUHK, Hong Kong, China; 2 Department of Mechanical Engineering, Stanford University, Stanford, CA USA; 3 Department of Surgery, Faculty of Medicine, CUHK, Hong Kong, China

**Keywords:** Mechanical engineering, Biomedical engineering, Electrical and electronic engineering

## Abstract

Tendon-driven continuum robots with spatial manipulability face fundamental challenges in miniaturization, stemming from the space required to accommodate multiple actuation tendons. Conventional multi-tendon designs create an inherent trade-off between miniaturization, 3D manipulability, and force output. Here, we introduce a class of continuum robots that achieves controllable body twist and full omnidirectional motion driven by pushing, pulling, and twisting a single tendon, breaking this long-standing design constraint. The resulting robot features an outer diameter of 2.0-3.5 mm and a circumferential hollow ratio exceeding 57%, nearly doubling spatial utilization efficiency over multi-tendon designs. Compared to conventional mechanisms, manipulability improves by over 1000-fold while retaining at least 70% of tip force across all directions. We derive the kinematics for this robot class and provide an open-source simulator. We demonstrate capabilities in teleoperation, navigation in tortuous environments, chopstick-like continuum grippers for in-gripper manipulation, and potential medical applications. Our design redefines actuation paradigms for tendon-driven continuum robots.

## Abstract (web summary)

Miniature continuum robots achieve omnidirectional motion by pushing, pulling, and twisting one eccentric tendon, enabling dexterous teleoperation and tortuous-path navigation and pointing to next-generation surgical actuators.

## INTRODUCTION

Continuum robots can be extrinsically actuated by tendons, cables, or rods for body deformation such that the distal end could reach a desired spot in 2D or 3D space^1^, which constitutes a robotic motion comparable to those of the rigid-bodied robots. Such motion is generated from the proximal base of the manipulator and transmitted along the neutral line (some refer to the backbone) by the motile mechanical elements^2^. With sufficient precision, dexterity, miniaturization, and mechano-conductivity^3^, soft continuum robots have great potential to assist minimally invasive surgery (MIS) and even single-port robotic surgery^4^.

Cable-driven (soft) continuum robots (CDSR/CDCR) usually employ the *pull-release* mechanism, as soft cables have difficulties in transmitting a pushing force. Therefore, elastic backbones, super-elastic materials, and soft elastomers are generally included in the robot composition^5–7^ so that the pulling-induced elastic energy can restore the robot’s shape. For tendon-/rod-driven continuum robots (TDSR/TDCR), an active push on tendons/rods in the axial direction (i.e., the *push–pull* mechanism) can generate visible robot deformation^8^, since the pushing forces are transmitted to the fixed-end through coaxial mechanical entities. A special case arises when the push–pull rod functions as an internal tube, concentrically aligned within the outer flexible robot^9–11^. Nonetheless, a tendon that is coaxially aligned with the robot’s neutral line would produce a 2D motion under the push–pull mechanism, whereas non-coaxially routed tendons are capable of versatile 3D motions despite the routing intricacy^12^. Employing multiple tendons/cables for omnidirectional motions on TDCR has been extensively studied over the past few decades^1^. Such well-established conventions have rarely been challenged, except for some bold attempts like introducing a rotatable base^13^, extensible segments^14^, and special tendon routing schemes^5,12^, among others. However, due to the need to accommodate sufficient space within the robot manipulator for actuation entities and multiple parallel motorized joints at the proximal end, these steerable robots face challenges in further miniaturization and usually suffer from a large footprint^1^. To achieve teleoperative spatial motions, multiple sections of these multi-tendon-driven units are required, which further complicates the system’s overall design. Flexible robots can achieve diverse motions, including twisting, and substantial miniaturization (e.g., 600 um outer diameter^15^) when implemented as origami structures^16^, pre-programmed pneumatic devices^17^, and magnetically driven systems^18^, and related architectures. Nonetheless, these novel structures and actuation methods may trade off spatial manipulability—the ability to move and exert forces in 3D space (see Supplementary Information)—and output force for motion versatility, which includes linear, bending, torsional, and spiral modes^19^, or vice versa. For example, for magnetically driven continuum robots, although magnetic actuation offers significant motion versatility, its effectiveness is constrained by the rapid decay of magnetic field strength with increasing distance from the source. This limits either the controllable working volume of the magnetic field or the magnitude of the actuation force that can be applied to the environment^19^. In origami-based structures, the soft robot’s tip exhibits certain spatial manipulability, but achieving versatile motion typically requires serially linking multiple soft modules^20^. In contrast, here, we consider the simplest form of a tendon-driven continuum robot, actuated by a single tendon joint that can be pushed, pulled, and twisted in a highly coupled manner. The re-engineered robot design and actuation mechanisms overcome the abovementioned long-standing trade-off, once considered fundamental in continuum robot design. Furthermore, the corresponding analytical model is derived and verified on various parametric robots.

Miniature TDCRs made of notched tubes were robotically described by York and Swaney^21^ in 2015. These types of continuum robots have asymmetric cutouts as notches in their tubular body (“side-notched tube”). With certain body elasticity, the notches constitute a compliant section that can be articulated by pulling a single tendon attached to the robot’s tip on the notched side, and restored to its original shape by releasing the tendon. They are mostly made from superelastic metals, such as Nitinol (nickel-titanium, or NiTi). The design parameters of the notches can be mechanically programmed by kinematics for designated bending behaviors^22^. Since notched-tube TDCRs are capable only of bending, they were initially referred to as needle-sized “wrists” upon their introduction. Nevertheless, when we examine the anatomy of the human forearm, we can observe that the forearm can perform supination and pronation – a twist-like motion enabled by the substantial rotation of the ulnar bone relative to the radius bone under the traction of oblique muscles. This anatomical characteristic effectively compensates for the limited lateral range of motion and power output of our wrist. Similarly, elephant trunks exhibit complex, multimodal deformations that enable highly dexterous movements. This capability arises from two distinct orientations of muscle fibers: longitudinal muscles, which facilitate bending, and oblique muscles, which are primarily responsible for twisting^23,24^. Biology has always been a source of inspiration for robotic innovation. This leads us to wonder: what if a continuum robot could twist like these biological manipulators? Can it improve motion versatility, spatial manipulability, or miniaturization?

Due to the simple monolithic structure and the advancement of precision manufacturing, notched tube robots have become one of the smallest tendon-driven robots with considerable force output for external manipulation, which is ideal for frontier robot-assisted MIS applications^25^. For example, Fichera et al.^26^ developed a miniature Nitinol-based asymmetrically notched tube TDCR as an endoscope to obtain vision inside the middle ear. While the asymmetric cutouts enable a bending-only motion, which is insufficient for 3D navigation in a sinuous environment, additional degrees of freedom (DOFs), including robot translation and rotation along its neutral axis, were added to their system. The 3D motion can be achieved in different ways. For instance, Zeng et al.^27^ developed a Nitinol-based ø 3-mm symmetrically notched tube CDCR for neurosurgery. Two pairs of actuation cables were used for pitch and yaw motions, utilizing a pull-release mechanism. In ref. ^28^, a 3D-printed notched soft tube TDCR was introduced with a pilot study of transoral robot-assisted tracheostomy puncture. Even though only a 1-DOF bending motion could be achieved, the robot could perform critical puncture due to the effective tendon-driven mechanism. By combining the unidirectional and bidirectional eccentric notched tubes on a two-segment TDCR, a 2-DOF *S*-shape bending tool can be realized^29^ for pediatric neurosurgery. More examples are summarized in ref. ^30^, suggesting that the simple notched patterns only permit either unidirectional or bidirectional bending. However, this is only true when a prismatic DOF, i.e., a push–pull tendon joint motion, is assigned.

Thus far, soft robots that can twist have attracted much attention in recent years^23,24,31^. These works are often inspired by biological manipulators, such as the elephant trunk—the contraction of radial muscles results in the extension of the trunk, while the contraction of oblique muscles induces torsion around the longitudinal axis. Additionally, the simultaneous contraction of longitudinal muscles on one side and radial muscles leads to bending toward that specific side, achieving omnidirectional bending motion within a compact manipulator. This principle shares common ground with the movement of the many mammalian manipulators (driven by radial and oblique muscles). By introducing a twisting or torsional motion, a versatile motion can be achieved. For example, it has been reported that a magnetically driven soft robotic crawler with an origami structure integrates stretching, folding, bending, and twisting to perform various functions such as grasping and lifting objects^32^. Similarly, an origami-based miniature continuum robotic manipulator with articulated modular actuators, designed to deliver bending or twisting motions, can holistically provide sophisticated spatial motions for tasks^33^. By introducing free-form apical and omnidirectional extension, Mao et al. report a magnetic field-driven vine-like continuum robot that enables complex twisted configurations, facilitating potential microsurgical applications that do not require manipulation^34^. With spatial manipulators over-twisting, a cluster of soft pneumatic strips entangle with each other and the target object, enabling grasping^17,35^. These works collectively demonstrate how soft robot twisting could bring motion versatility, but complicate the robotic structure and ease of fabrication. Despite significant advancements in simulation technologies and one-off characterizations, establishing parametric descriptions for robot design and control, particularly for systems involving twisting motions, remains a challenge. This standardization is essential for ensuring the reproducibility of soft robotic systems^36^.

**Fig. 1:**
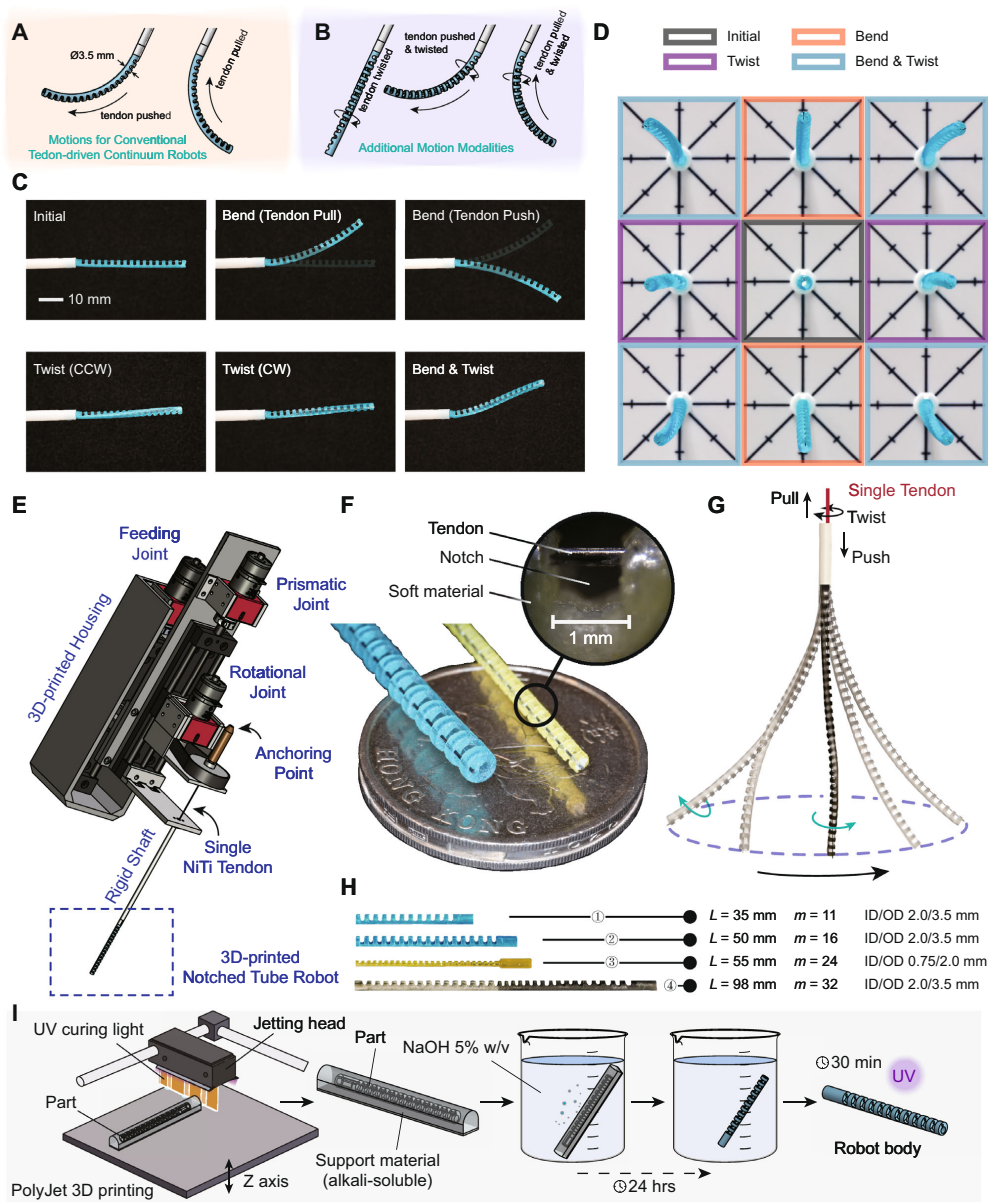
A conceptual schematic illustrating how the proposed single-tendon-driven mechanism will drive a notched tube continuum robot, and the robot design. **A** Robot motion is achieved by conventional push–pull actuation. **B** Additional motion modalities can be achieved by twisting the tendon. **C** We fabricate a sample notched tube continuum robot to verify the robot motion enabled by only actuating a single tendon. The photos above show different continuum robot motions. These are from the side view, and **D** from the front view. Also see Movie S1–S2. (CW Clockwise, CCW Counterclockwise). **E** The compact robot design (230 mm × 105 mm × 60 mm, 1 kg) facilitates three motorized joints. **F** Zoomed-in view of the miniature robots alongside a coin for scale. A microscopic view of a 3D-printed notch is also shown. **G** PPT mechanism acting on the single tendon to produce 3D robot motion. **H** Four miniature continuum robots (indexed as ①–④) with distinctive geometric parameters in length, number of notches, and ID/OD are produced for the experiments. **I** All the continuum robots are 3D-printed under a rigorous post-processing protocol to ensure production consistency. The robots are printed in batches with sufficient alkali-soluble support material. The semi-finished robots are then immersed in a sodium hydroxide aqueous solution (5% w/v) for 24 h, followed by an ultraviolet light bath in a 60 °C drying chamber for 30 min.

In this work, we present a bio-inspired, omnidirectional, notched tube tendon-driven continuum robot that utilizes a single push–pull–twist (PPT) tendon joint, designed for miniaturization and enhanced spatial manipulability (Fig. 1). The proposed PPT tendon joint mechanism, previously undescribed, fundamentally advances the capabilities of notched tube continuum robots by extending beyond conventional unidirectional and bidirectional bending to enable axial twist and near-helical motion. Consequently, tendon-driven robots can be dramatically miniaturized by relying on a single tendon, and most importantly, without substantially compromising mechano-conductivity. The eccentrically placed tendon with the twistable motion can significantly improve the manipulability of the distal tip, facilitating a variety of potential applications where robot dimensions are highly constrained. To understand the functioning of the PPT tendon joint mechanism, this study investigates its behavior in representative TDCRs—primarily those with a hollow working channel—focusing on kinematics, performance, and potential applications that could leverage the additional twisting motion it provides. To the best of our knowledge, this is the first general kinematic model proposed for single-tendon-driven continuum robots incorporating PPT tendon joints. We anticipate that this work will inform future studies on tendon-driven continuum structures at a fundamental level, laying the groundwork for advances in design, optimization, control, and application.

## RESULTS

### Robot design

Our continuum robot adopts the asymmetric notched tube structures that enable unidirectional bending. To facilitate the torsion effect on the notched tube, materials with low shear modulus shall be selected to fabricate the continuum robots. On the contrary, the tendon should be made from materials with a relatively high shear modulus to achieve visible torsion and bending in the continuum robot. Therefore, the continuum segment is selected to be a multi-material-based soft tube (Shore hardness below 60 Scale A) containing lateral cutouts that cause it to deform, as a single tendon on the cutout side attached (glued) to the distal tip is pushed, pulled, or twisted from the proximal anchoring point enabled by a copper collet (Fig. 1E). Either NiTi (55 wt% of Ni) wire or fishing line can be used as tendons. In this work, NiTi was used. To facilitate a compact design, we superimpose the robot’s prismatic and rotational joints at the same point on a tendon. As shown in Fig. 1E, the prismatic joint is enabled by a motorized screw lead that induces linear motion of the tendon along the axial direction of the TDCR, which gives conventional push–pull forces to the tendon. The rotational joint is motorized by another independent direct current (DC) motor attached to a slider controlled by the prismatic joint. The rotational joint and prismatic joint are relatively fixed to each other, which forms the core actuation unit for the PPT tendon joint mechanism. To enhance the output torque of the rotational joint, we utilize a reduction synchronous belt pulley drive (1:3) and formulate a non-coaxial design for ease of robot assembly and disassembly (Supplementary Fig. S1). The PPT joint and the TDCR manipulator are fixed onto another prismatic joint (feeding joint) at the bottom layer to provide an overall insertion–retraction motion along the robot axis, such that the system processes, in principle, three DOFs for basic 3D motion. Consequently, the TDCR can be steered in 3D space via the PPT mechanism, even with only a single tendon being actuated (Fig. 1G). Representative motion modalities of one of the TDCRs are vividly demonstrated in Movies S1–S2.

In this study, we report four miniature continuum robots with distinctive geometric parameters (Fig. 1H and Supplementary Table S1–S4) to verify our concept. Their lengths (*L* = {35, 50, 55, 95}), number of notches (*m* = {11, 16, 24, 32}), and the ratio of inner to outer diameter (ID/OD  = {1.8/3.5, 1.8/3.5, 0.5/2.0, 1.8/3.5}) are specifically selected to facilitate cross-reference experiments. These continuum robots are 3D-printed under a well-controlled fabrication and post-processing protocol (Fig. 1I) to ensure consistent stiffness, strength, and durability. The miniature outer diameters of our robots allow them to pass through most commercially available endoscopic channels (see Table in ref. ^37^).

**Fig. 2:**
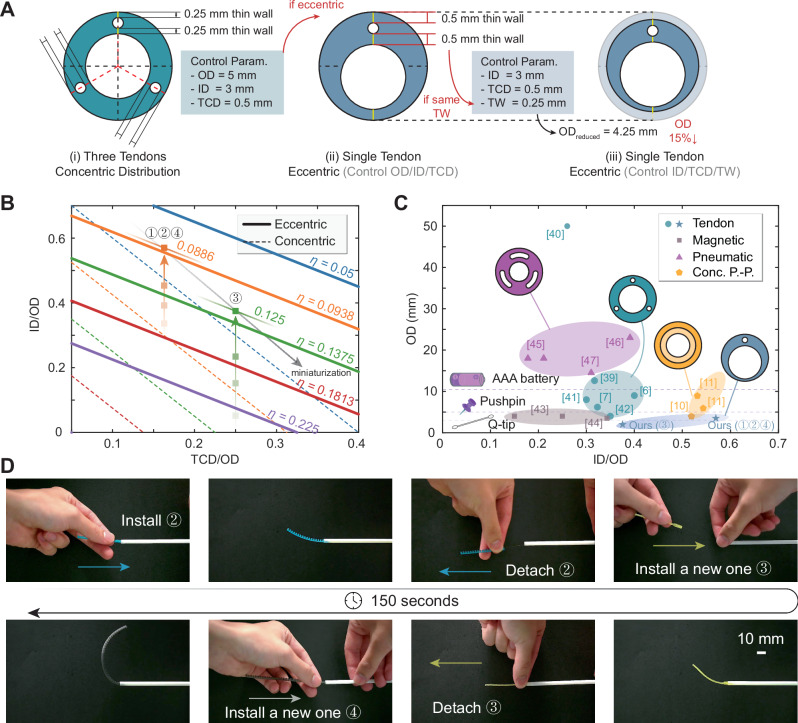
The employment of single-tendon fashion for TDCRs reduces the robot's dimension and facilitates robot assembly. **A** Having an eccentric working channel can reduce the robot’s outer diameter (OD) and increase the ratio of the inner-outer diameter (ID/OD), given the same control parameters, including the inner diameter (ID), tendon channel diameter (TCD), and thin wall (TW) thickness. **B** Compared to the traditional symmetric tendon channel layout, the ID/OD ratio can be improved when employing an eccentric layout (*η* = TW/OD), resulting in higher space efficiency. **C** Compared to other soft continuum robots, our single-tendon-driven design achieves one of the highest ID/OD ratios while maintaining an OD comparable to magnetically driven miniature continuum robots. **D** The single-tendon paradigm enables rapid assembly and disassembly of the disposal continuum. For example, three continuum robots (robot ② → ③ → ④) of different sizes can be conveniently switched for motion within 150 seconds.

Adopting single-tendon actuation can reduce the dimensions of TDCRs without compromising the size of the inner working channel under identical fabrication conditions, thereby extending the upper limit of the circumferential hollow (ID/OD) ratio for steerable miniature continuum robots, particularly those fabricated from soft materials. For example, consider a traditional TDCR with three concentrically distributed tendons, having an OD of 5 mm, an ID of 3 mm, and a tendon channel diameter (TCD) of 0.5 mm, as shown in Fig. 2A(i). Its thin wall (TW) thickness would be 0.25 mm. By controlling the OD, ID, and TCD, and employing a single-tendon eccentric cross-sectional design, the minimal TW thickness would double to 0.5 mm, resulting in a stronger tendon channel (Fig. 2A(ii)). If the fabrication conditions allow for a minimum TW structure of 0.25 mm (regardless of durability), the robot’s OD, with the same ID and TCD, would be reduced to 4.25 mm (Fig. 2A(iii)), achieving a 15% reduction in OD. In this regard, we further estimate the relationship between TCD/OD and ID/OD under various TW/OD ratios (denoted as *η*) to evaluate how the eccentric design can enhance the ID/OD ratio compared to the traditional concentric design with controlled parameters (Fig. 2B, see Supplementary Information). The result suggests that the eccentric design can substantially increase the ID/OD ratio. We also compare to other soft continuum robots driven by tendons, external magnetic fields, and fluid (Fig. 2C). The comparison reveals that our prototyped single TDCRs excel in room efficiency and robot dimensions among other steerable continuum robots (viz., tendon-driven^7,38–42^, magnetically-driven^15,43,44^, pneumatically-driven^45–47^), and most concentric push–pull robots^9–11^. It is worth noting that emerging concentric push–pull robots are constructed from nested thin-walled tubes; consequently, a larger outer diameter, for instance, those in centimeter-size, naturally yields a higher circumferential hollow ratio^11^. While recent progress in elastic metals and laser machining could further advance their miniaturization^9^, such approaches are not well-suited to soft-material-based continuum bodies. In a word, our robot designs offer effective solutions to the challenges of continuum robot miniaturization, ensuring the preservation of the working channel and, thanks to the tendon-driven mechanism, maintaining a high force output for interaction.

Additionally, our design simplifies the robot’s assembly and disassembly due to the PPT mechanism requiring only a single tendon for actuation. For assembly, one can simply thread the NiTi tendon from the distal end of the rigid shaft to the proximal anchoring point (Fig. 1E, F), then hand-tighten the tendon to the copper collet (the anchoring point). Conversely, detachment follows the same process in reverse. As demonstrated in Fig. 2D (and Movie S3), exchanging different continuum robots can be completed within 10 s. We show that three different continuum robots can be conveniently switched one by one for effective motion within 150 s—which was unimaginable for the majority of TDCRs following the traditional multi-tendon actuation schemes.

### Robot characterization

**Fig. 3:**
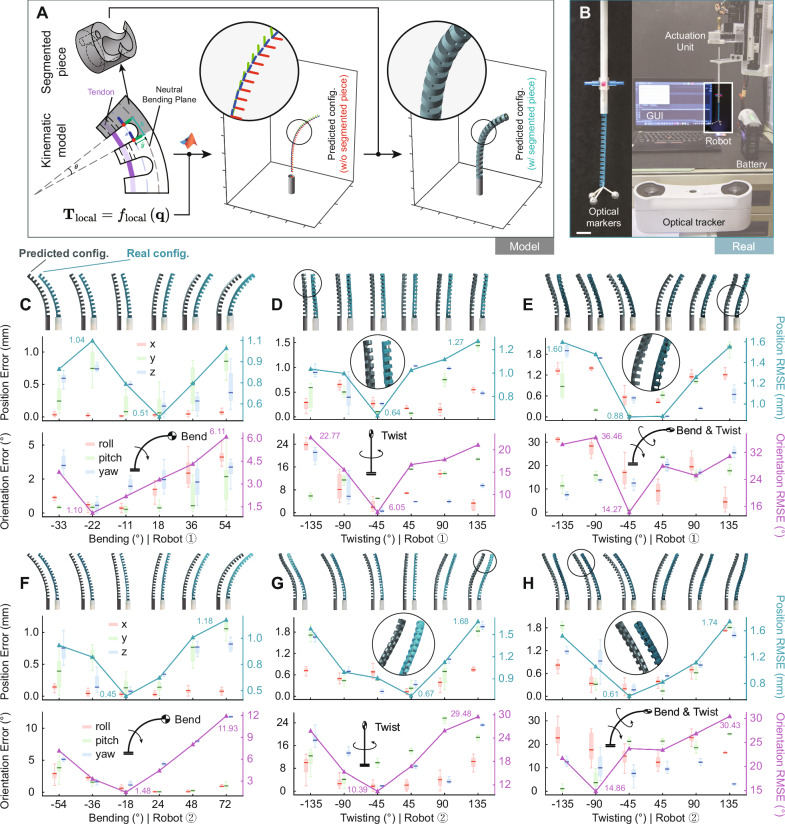
Verification of the newly derived kinematic model on two robots in different lengths (Robot ① with an aspect ratio of 10, and Robot ② with an aspect ratio of 14.29). Each configuration undergoes 10 repeated experiments (i.e., 360 tests in total), comparing the real-world tip position and orientation with the kinematically computed values. The boxplot shows the 25th to 75th percentiles of the trials, with the median indicated in darker bars. The right ordinates represent the root mean square errors (RMSE). **A** Simulator design. **B** Experiment setup for tip pose measurement (scale bar denotes 10 mm). **C** Robot ① bends from -33° to 54°. **D** Robot ① twists from -135° to 135°. **E** Robot ① twists from -135° to 135° in a 36° bent configuration. **F** Robot ② bends from -54° to 72°. **G** Robot ② twists from -135° to 135°. **H** Robot ② twists from -135° to 135° in a 48° bent configuration. The robots’ parameters can be found in Supplementary Table S1–S2. A zoomed-in view of the simulated and real robots is available in Supplementary Fig. S5.

The kinematics of our TDCR with PPT mechanism is established based on the model of an asymmetric (eccentric) continuum robot that only considers 2D bending^21,29^. The newly derived model, explicitly presented in the “Methods” section, takes into account the geometric parameters, eccentric notched tube structure, and tendon twist to describe the quasi-static motion of the robots. To facilitate the analysis, we developed a graphical kinematic simulator on top of our previous open-source work^48^ using mathematical software (MATLAB R2022a (academic use), MathWorks). The developed simulator allows morphological visualization of the model-based robot configuration (represented by finite discrete Cartesian frames equidistantly attached to the cross-sectional centroid) using joint-level actuation as input (Fig. 3A), and also permits optimization-based inverse kinematics. Except for the remote-controlled teleoperation (Supplementary Fig. S2), a graphical user interface (GUI) was built to assist with the precise teleoperation (Supplementary Fig. S3). To obtain the distal position and orientation of the tested TDCRs, we used an optical tracker (fusionTrack 250, Atracsys, Switzerland) to track an optical marker (Fig. 3B). Since the testee robot is known, the robot base frame can be estimated by subtracting the robot length. The details of the optical marker and tracking system are provided in Supplementary Fig. S4.

To showcase modeling accuracy, we compare the model-predicted configuration with the real-world measured configuration of the robot tip. Here, Robot ① (35 mm length, Supplementary Table S1) and Robot ② (50 mm length, Table S2) were selected. The robots were teleoperated to deform in three modes: pure bending (Fig. 3C and Fig. 3F), pure twisting (Fig. 3D and Fig. 3G), and twisting while bent (Fig. 3E and Fig. 3H). Each configuration was tested 10 times to produce a reliable median error, and six configurations were examined for each mode. Note that the orientation root mean square error (RMSE) is calculated as the axis-angle deviation between the prediction and the measurement. A zoomed-in view of this figure is available at Supplementary Fig. S5. For pure bending (Fig. 3C and Fig. 3F), both robots demonstrate minor positional (≤1.18 mm) and orientational RMSE (≤11.93°). The longer robot exhibits a larger orientation error, approximately twice the error of the shorter robot. For pure twisting, a straight initial configuration was first ensured, followed by bidirectional twisting to −135° and 135°, respectively. It can be observed from the simulation and experiment that the longer robot shows significant deviation from the robot centroid axis (Fig. 3G), rather than exhibiting a pure axial twist. This counterintuitive deformation—which we termed as twisting-induced bending—can be kinematically described by our model with a positional RMSE of 0.64–1.68 mm and an orientation RMSE of 6.05–29.48°, verified by our sample robots. Fig. 3E shows Robot ① twists toward −135° and 135° in a bent initial configuration (36° w.r.t. the robot centeroid axis), indicating a positional RMSE of 0.88–1.60 mm, and 14.27–36.46° in orientation. Our model demonstrates no significant error deterioration in such a highly coupled twist-while-bent case for the longer robot (with a bent initial configuration of 48°, as shown in Fig. 3H), provided by a 0.61–1.74 mm and 14.86–30.43° RMSE in tip position and orientation, respectively. It should be noted that these results are based on miniature robots. Even though a 30° orientational error exists, the overall robot configurations align well. These results also indicate that the accuracy of the model prediction decreases in large bending and twisting motions. Therefore, it can be concluded that our model can effectively describe the different miniature TDCRs’ static motion under the newly proposed PPT joint mechanism, with the tip positioning error below 1.74 mm and orientation error below 36.46°.

**Fig. 4:**
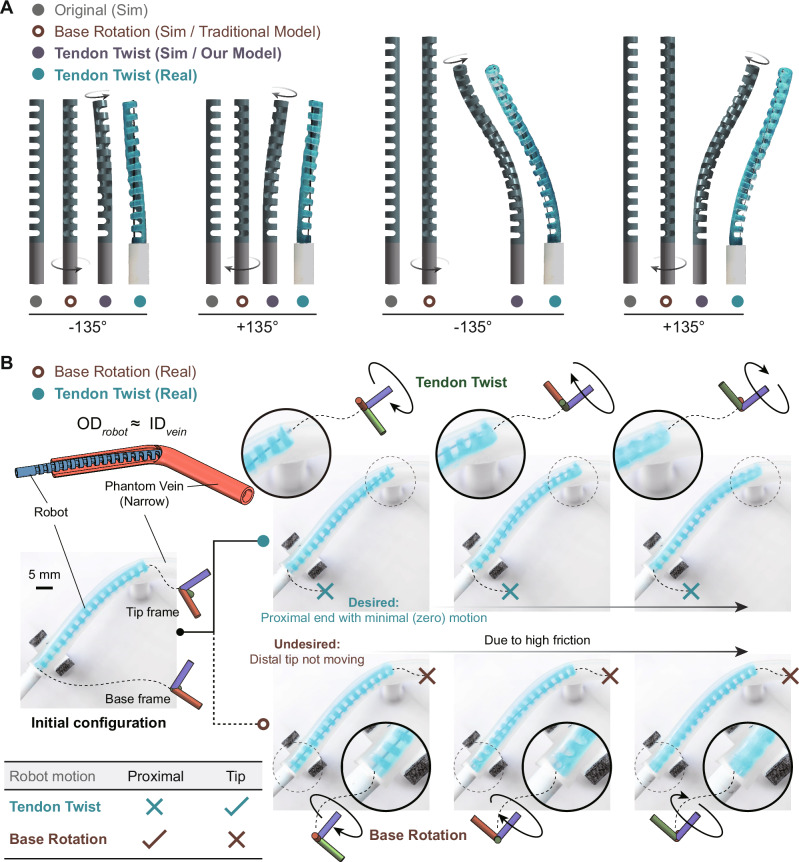
Comparison of the traditional holistic base/robot rotation model with the proposed twistable tendon model. **A** We capture twisting-induced bending effects by presenting kinematic simulations of two robots (Robot ① and ②) under  ± 135° base rotation and pure tendon twist, together with the corresponding real-world robot actuated by tendon twist. The traditional approach fails to capture twisting-induced bending, which is non-negligible in long robots, whereas our model can describe the resulting shape and morphology, demonstrating its practical advantages. **B** In a mock intravascular operation setting, the robot–designed with a diameter comparable to the vein’s narrow inner lumen–demonstrates that tendon twisting can transmit rotation to the functional distal tip with minimal (or even zero) motion at the proximal end. In contrast, the traditional approach of rotating the entire continuum often fails to actuate the distal tip effectively due to high robot-environment friction.

To investigate twist-induced bending, Fig. 4A compares the traditional holistic base/robot rotation model with our proposed twistable tendon model. Unlike most existing approaches that focus solely on base or robot rotation—which fail to capture the non-negligible bending effect—our model advances the state of the art by incorporating tendon twist, accurately delineating the robot’s morphology with notch-level resolution and substantial twist-induced bending. In Fig. 4B, we compare the tendon twisting and base rotation mechanisms in a mock intravascular operation setting, where the robot has an outer diameter comparable to the inner lumen of a narrow soft vein (Ecoflex 00-10 silicone, Smooth-On Inc., USA). High robot–environment friction can hinder the steerability of the functional tip when rotating the robot base—a widely employed convention for steering intravascular tools—since insufficient torque is transmitted to the distal end and instead concentrates at the proximal end. On the contrary, by incorporating the twistable tendon mechanism, the torque can be effectively applied to rotate the tendon that is anchored at only two fixed ends, thereby transmitting torque to the distal tip and directly transferring rotation to the far end. This could be useful to advance continuum robot manipulation in extremely narrow lumens. Compared with traditional methods, our PPT tendon joint mechanism shows strong potential for interactive manipulation of continuum robots in highly confined anatomical pathways, such as vascular interventions and bronchial terminals.

### Workspace, manipulability, and output force performance

**Fig. 5:**
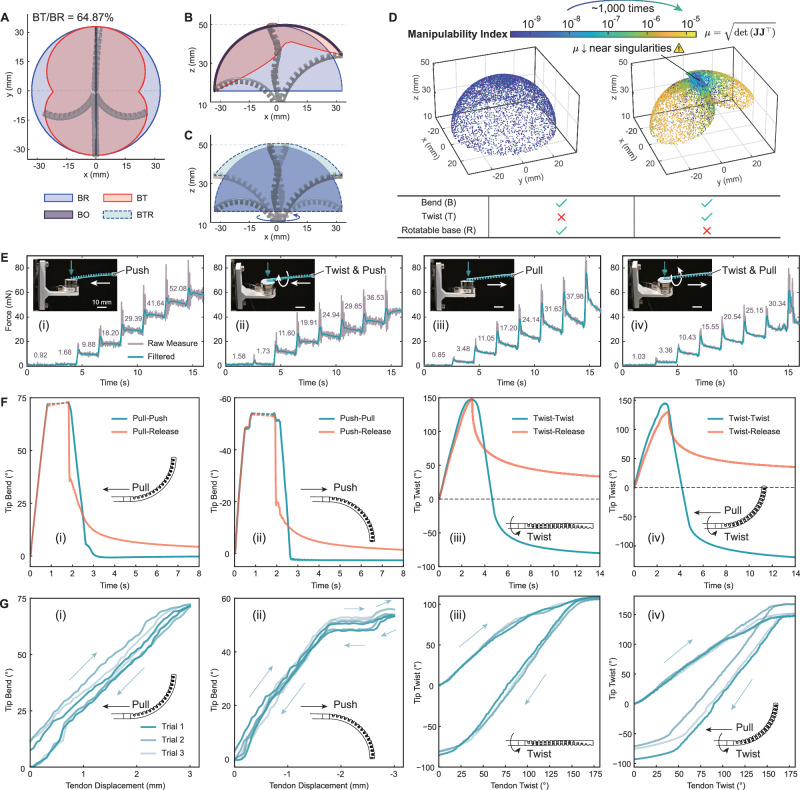
Motion and force performance of the TDCR (Robot ②) under the single tendon PPT mechanism. **A** Workspace comparison (top view) of the same robot under various actuation mechanisms, including bending & rotatable base (BR), bending & twisting (BT), bending only (BO), and bending & twisting with a rotatable base (BTR). **B** Side view of the workspace comparison among BO, BR, and BT. Note that tendon pulling and pushing produce distinct bending angles under equal actuation. **C** Side view of the workspace comparison between BR and BTR. **D** The manipulability of the robot tip among BR and BT indicates that the tendon bending-twisting mechanism improves over 1000 times in most configurations compared to the traditional bending-rotating base mechanism. Note that the manipulability is configuration-dependent. **E** Tip lateral force exertion under different modes of motion, viz., bending (pushing/pulling) and bending with twisting (pushing/pulling & twisting). **F** Hysteresis tests for different modes of motion, including (i-ii) bending, (iii) twisting, and (iv) coupled motion. We compare the hysteresis effect between the opposite joint motion (ours) and the actuate-release joint motion (a commonly adopted mechanism for other TDCRs), evidencing significant hysteresis in joint twisting in both cases. **G** Hysteresis tests with repeated trials for different modes of motion illustrating the joint-tip motion relationship.

The proposed PPT mechanism offers the robot a distal workspace distinct from the traditional push–pull mechanism on a rotatable robot base. To evaluate the impact on tendon twisting motion and understand how this mechanism compensates for the loss of additional workspace provided by a rotatable robot base, we primarily compare the theoretical distal workspace between bending on a rotatable base (BR) and bending with twisting (BT) on the same Robot ②. Additionally, we examine a more comprehensive motion involving bending with twisting on a rotatable base (BTR). The configuration used for workspace computation is described in the Methods section. Since the distal workspaces are not volumetric but rather form shell-like curved surfaces, we primarily visualize them through two-dimensional projections (Fig. 5A–C). For Robot ②, the traditional BR motion demonstrates a hemispherical shell-like workspace, while the BT motion induced by the PPT mechanism produces an asymmetric shell. We estimate the curved surface areas of BR and BT using triangulated meshes (with code provided in the supplementary), and find that the area of BT is approximately 64.87% of that of BR. The pulling-induced bending side coincides with the BR robot motion, and the pushing-induced bending side extends toward the *x*-axis direction (Fig. 5B) due to the robot elongation. It should be noted that, in our analysis, tendon pulling and pushing produce unequal bending angles under identical actuation levels. Nonetheless, the twisting can exceed ±90°, and the bending can reach up to over 180° on both sides, thereby providing a higher percentage of workspace restoration. Introducing an additional rotating base for the TDCR with a PPT mechanism can enhance the workspace, exceeding the workspace provided by the conventional BR mechanism (Fig. 5C).

The proposed PPT mechanism contributes a substantial enhancement to the robots’ manipulability. This is evidenced by calculating the manipulability index (see Supplementary Information). The manipulability index in robotics, introduced by Yoshikawa in 1985^49^, is a measure of how easily a robot can move its end effector in different directions from a given configuration. For the 2-DOF systems, the theoretical manipulability index remains around 10^−8^ for BR motion and 10^−5^ for BT motion across the workspace, meaning BT is roughly 1000 times higher than BR (Fig. 5D). It is noteworthy that the manipulability index for BR motion remains nearly uniform across the entire workspace, whereas for BT motion it varies substantially: the index decreases near singular configurations, while other regions exhibit consistently higher manipulability. Therefore, the improvement in the manipulability offered by the PPT joint can efficiently extend the dexterity of the existing tendon-driven mechanism on a single tendon.

To evaluate the output forces, we examined the lateral force exertion under normal bending motions and twisting-bending motions (Fig. 5E). A 6-axis force sensor (Nano17, ATI Industrial Automation, USA) was employed for the measurements, positioned to align with the tip bending direction to accurately capture the resultant force. A 2-s pause was programmed to allow for a gradual increase in bending motion (joint space level). As illustrated in Fig. 5E(i), the robot bends towards the force sensor via tendon pushing, achieving a maximum static exertion force of approximately 60 mN. In a 30° twisted configuration (Fig. 5E(ii)), the same joint space bending motion yields a similar force output profile, albeit with a reduction of 20–30% from the bending-only scenario. A comparable force output profile is observed in tendon pulling-induced bending motions (Fig. 5E(iii–iv)), with a maximum exertion force of around 40 mN. Additional results on measuring the force output on the non-bending-perpendicular planes under the same configuration settings (as in Fig. 5E) are provided in Fig. S6. Consequently, it can be concluded that the twisting-bending configuration retains at least 70% of the exertion force at the distal tip across various directions compared to the default non-twisted bending direction, demonstrating effective omnidirectional manipulability for the TDCRs.

Hysteresis arising from tendon friction and flexural deformation is generally observed in tendon-driven notched tube continuum robots^50^. To evaluate the hysteresis of the proposed PPT joint motion in soft notched tube TDCRs, we conducted controlled experiments comparing it with the actuate-release joint motion, a commonly adopted mechanism in many other multi-TDCRs. Various modes of motion, including bending, twisting, and coupled motion, were examined on Robot ②. The tip bending/twisting angle was computed based on the optically measured tip position (Fig. 3B). Specifically, the robot was first bent via a 3-mm tendon pull, then returned to the zeroing joint status via pushing. In the controlled experiment, the tendon was released by manually loosening the copper collet (the anchoring point) after achieving the same bending motion. As shown in the results (Fig. 5F(i)), the pull-push joint motion demonstrates a trapezoidal bending deformation profile over time, indicating low hysteresis in the pulling-induced bending. Conversely, the pull-release joint motion exhibits rapid deformation upon tendon release and fails to return to the initial pose. A similar result is observed in the pushing-induced bending case (Fig. 5F(ii)). In an active tendon-twisting case (Fig. 5F(iii)), the forward and inverse twisting (from 0° to 175°, and from 175° to 0° in the joint space) show a more symmetrical plotting profile than the twist-release convention, suggesting a highly maneuverable angular velocity in robot twisting. However, reverse tendon twisting, even when ending at the zeroing position, results in over-twisting tip motion in the opposite direction with substantial hysteresis, whereas the twist-release method results in insufficient reverse motion (i.e., under-twisting), failing to return to the initial robot pose. The over-twisting occurs due to residual stress in the low-elastic soft material, which is subjected to high angular acceleration of the tendon. A similar result can be observed in the case of bending-twisting coupled motion (Fig. 5F(iv)). The observed motion hysteresis is primarily caused by amplified tendon-routing friction during twisting, together with the viscoelasticity and residual stress of the soft material. Nevertheless, hysteresis can be easily addressed using closed-loop control methods^11,50,51^, which are beyond the scope of this work. Fig. 5G presents the corresponding repeated trials (three times) for the results shown in Fig. 5F, reaffirming the robot motion hysteresis, particularly when tendon twisting is involved. Additionally, it is observed that tendon pushing can elongate the robot’s length under excessive bending (Fig. 5G(ii)), resulting in a plateau in the bending angle. This plateau can be overcome by further stretching the tendon. The difference in bi-directional bending aligns with our modeling and workspace analysis (Fig. 5B–D).

### Navigating through tortuous environments

**Fig. 6:**
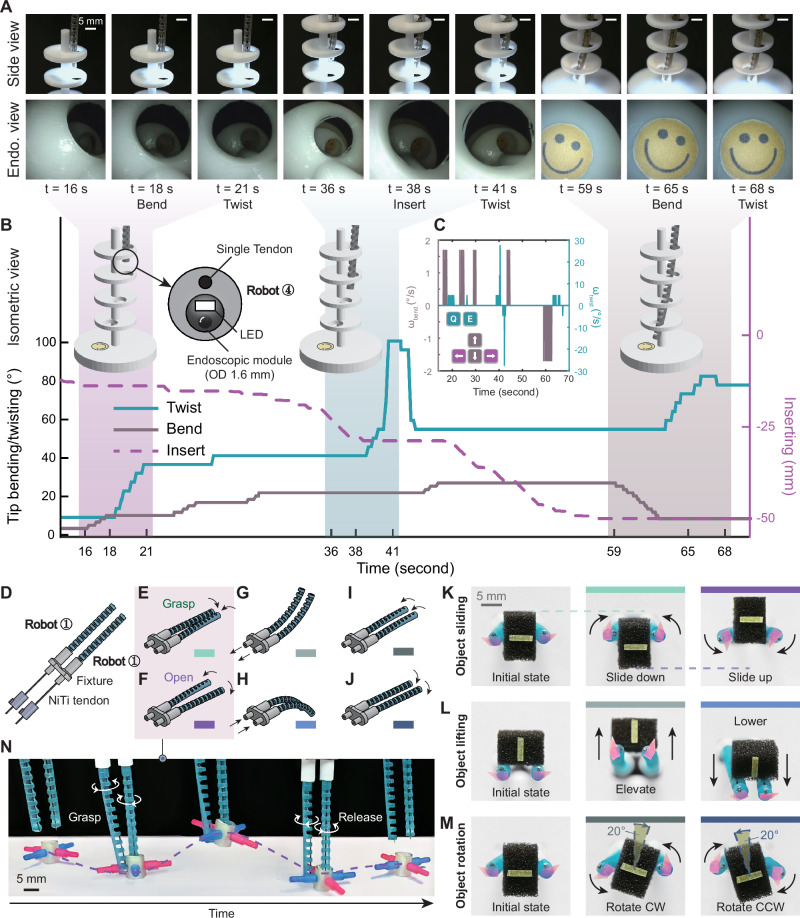
The PPT mechanism enables the TDCR to navigate tortuous environments. When paired, two such continua form a chopstick-like soft gripper capable of in-hand manipulation and grasping of tiny, soft objects. **A** Robot ④ passes through a four-story tower with a non-coaxial passage on each floor. The tortuous experimental setup requires the robot to have spatial mobility for navigation and axial rotation/twist to adjust its distal tip’s vision. **B** The robot was teleoperated and equipped with a commercial endoscopic module. Sufficient twisting up to 100° was required for this task. **C** The robot was set to bend and twist at a maximum distal deformation speed of ±1.69°/s and ±27.48°/s, respectively. **D** The soft gripper is made of two identical but independently controlled TDCRs (Robot ①, each weighing 0.105 g) and is installed in parallel on a fixture. The soft gripper offers various motions, including **E**, **F** object grasping and in-hand object sliding, **G**, **H** object moving, and **I, J** in-hand object rotation. **K** Experiment demonstration on object sliding, **L** object lifting, and **M** object rotation. **N** The gripper can tightly grasp a non-standard object (0.405 g) for transportation.

The PPT mechanism enables the TCDR to navigate through tortuous environments. To showcase its 3D navigation ability, we set up a four-story tower (3D printed, see Supplementary Fig. S9), with a non-coaxial passage on each floor, for the robot to pass through from top to bottom (see Fig. 6A and Movie S4). The non-coaxial passages create a highly tortuous environment (i.e., a helical path), requiring the robots to possess sufficient spatial mobility to navigate from top to bottom without major collisions or getting stuck midway. On the bottom floor lies a target pattern, necessitating the robot tip to either rotate or twist along the robot centroid axis to perceive a non-skewed view from its endoscopic view (OV6948, OmniVision, USA). Using only the endoscopic view, we teleoperated the robot with keyboard input (see Supplementary Fig. S3) to navigate. Twisting motions play a critical role in reorienting the bending direction, with axial twists of up to 100° employed. (Fig. 6B). Moreover, the robot was set to bend and twist at a maximum distal deformation speed of ±1.69°/s and ±27.48°/s, respectively (Fig. 6C), such that the navigation task can be completed in about a minute. In a comparison experiment where the TDCR was set to bend-only motion (see Movie S5), the robot failed to navigate through the second passage. This failure occurred because the centers of these passages were not aligned in the same plane, exceeding the robot’s workspace capabilities without the ability to twist.

Moreover, from a human-robot interface perspective, twisting motion, though mechanically advantageous, rotates the endoscopic view with the robot’s body—similar to other steerable endoscopes that rotate along their flexible shafts—potentially disorienting the operator and increasing cognitive demands. This drawback can be mitigated by applying a derotation (de-rolling) algorithm to the camera view, either by compensating for predictable tip twisting or by using computer vision methods that periodically recognize visual landmarks^52^. While such approaches may improve the operator’s visual orientation, they could also disrupt the sense of teleoperation. The presented experiments did not implement a derotation algorithm, as this lies beyond the scope of the present study.

In summary, the experiment demonstrates that the proposed PPT mechanism endows single TDCRs with 3D motion capability for navigating tortuous environments, potentially enhancing the motion complexity of a wide range of TDCRs that utilize single or multiple actuation tendons. This can be useful when the rotational motion of a robot base is not feasible, such as in confined task spaces.

### Chopstick-like dual continua-based in-hand manipulation

Due to its minimalist single-tendon-driven structure, the proposed continuum robot can be conveniently modularized to assemble diverse robots with multiple continua. To demonstrate its potential extensibility, we developed a chopstick-like dual continua-based soft gripper for delicate object manipulation (see Fig. 6D). Traditional examples include the development of a two-fingered micromanipulator hand based on a parallel kinematics chain mechanism^53^, similar to how chopsticks are used. These parallel rigid grippers are typically driven by piezo-electric actuated prismatic joints, enabling precise control and manipulation of delicate microscopic objects. However, they lack compliance with the objects and the ability for in-hand manipulation^33,53,54^.

In this regard, we utilized two identical but independently controlled TDCRs (Robot ①, each weighing 0.105 g) and installed them in parallel on a fixture to create a dual continua-based soft gripper (or tweezer), as shown in Supplementary Fig. S11. By combining various bending and twisting motion modalities on different robots, the gripper can achieve object grasping and in-hand object sliding (Fig. 6E, F, K), object lifting (Fig. 6G, H, L), and in-hand object rotation (Fig. 6I, J, M), also shown in Movie S6. This is feasible due to the friction between the robot and the object. When the tendons are twisted in opposite directions, the deviation of the two distal tip positions creates a closing and opening movement, making the gripper suitable for grasping delicate objects. Additionally, this motion pattern enables in-hand object sliding (Fig. 6K). When the tendons are twisted in the same direction, the grasped object can be rotated, with the rotation angle depending on the object’s shape. In our sample trials, the assembled soft gripper was able to rotate a soft sponge cube up to ±20° without causing any visible deformation on the sponge cube. This capability is highly desirable for many precise grasping tasks involving soft, fragile objects.

In addition, the grasping performance on various objects was verified. As shown in Fig. 6N, we used the soft gripper to grasp a 3D-printed cylindrical part (0.405 g) and transport it from point to point. The test demonstrated that the gripper can securely hold the target object even under manual jittering (see Movie S7). Additionally, various objects with distinct shapes and weights were tested, as shown in Fig. S10A and Movie S8, including a wire (0.388 g), a Panadol tablet (0.875 g), an M6 screw nut (2.05 g), and a 10 mL syringe (6.76 g). Despite their varying shapes, these objects correspond to 1.85 times, 4.17 times, 9.76 times, and 32.19 times the soft gripper’s net weight (0.21 g), respectively (see Fig. S10B).

In summary, the results demonstrate that the dual continua-based soft gripper utilizing the PPT joint mechanism is proficient in in-hand manipulation of tiny soft objects (sliding, lifting, and rotation) without deforming them, and is capable of adaptive grasping of various objects, in comparison to rigid manipulator-based grippers.

### Flexible otoendoscopic robot

The proposed TDCR with the PPT joint mechanism can facilitate flexible otoendoscopy (endoscopy of the ear) for safer and multifunctional transcanal operations. Traditional otoendoscopy involves inserting a long, rigid endoscope (connected to a fiberoptic light source) into the ear to examine both the exterior and middle portions. These tools enable surgeons to visualize high-resolution anatomical details at close range, allowing for procedures such as the removal of foreign bodies, otomycotic flakes, and aural polyps. However, there are two drawbacks to traditional otoendoscopes. First, existing rigid otoendoscopes are generally made from metal, which can potentially harm the patient’s ear if the patient (denied anesthesia) makes sudden movements (like sneezing). Secondly, during object removal procedures, surgeons are required to hold the otoendoscope in one hand and manipulate another instrument, like a gripper, with the other hand. Surgeons must keep their arms raised in mid-air throughout the procedure, which can lead to fatigue and exhaustion. Therefore, having a flexible robotic otoendoscope can be beneficial.

**Fig. 7:**
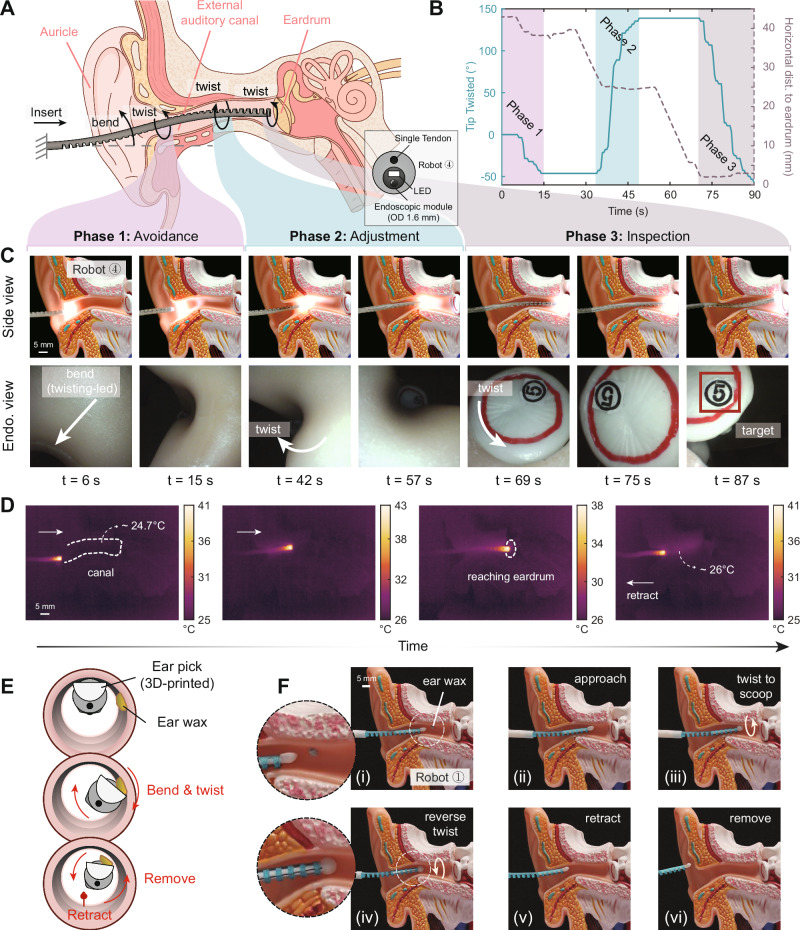
The miniature robot as a teleoperative multi-functional flexible otoendoscope. **A** The steerable bending and twisting motions accommodate the curved and varied structures of the auditory canal among patients. **B** When using the single-TDCR (Robot ④), three major phases of twisting motion are identified: avoidance near the entrance, adjustment within the ear canal, and inspection of the eardrum. **C** Our mockup trial on a 1:1 human ear anatomy phantom demonstrates the efficiency (<90 s) of the robot-assisted eardrum inspection process, **D** without major collisions or LED-induced thermal damage to the canal (<1.3°C temperature rise after robot retraction). **E** Additionally, utilizing its manipulability, the robot can serve as a teleoperative carrier for personalized tools used in object (e.g., earwax) removal from the canal. **F** Our mockup trial (Robot ①) demonstrates that the bending and twisting motions facilitate effective robot-assisted ear wax removal.

In Fig. 7, we demonstrate that the proposed TDCR can potentially be used as a teleoperative otoendoscope, offering safe robot-environment interaction (soft material-based compliant structure) and high stability (roboticized). We utilized Robot ④ (OD: 3.5 mm) as the robotic carrier to host an endoscopic module (OD: 1.6 mm, OV6948, OmniVision, USA) within its working channel, creating a steerable flexible otoendoscope (see Fig. 7A). The steerable bending and twisting motions accommodate the curved and varied structures of the auditory canal among patients, making it adaptable to environmental changes. In a mockup trial on a 1:1 human ear anatomy phantom (see Fig. 7B–C and Movie S9), we identified three major phases where twisting motion is necessary: avoidance near the entrance (Phase 1), adjustment within the ear canal (Phase 2), and inspection of the eardrum with a right-up view (Phase 3). In Phase 2, the robot twisted approximately 200° to adjust its body shape in adaptation to the curved canal, ensuring minimal collision. At the final stage of Phase 3, the twisting motion was used for view adjustment and tip re-location around the eardrum. The entire teleoperation was completed within 90 s, and we envision that such a procedure can be made more efficient with artificial intelligence (AI) techniques like feature learning-based autonomous navigation^7^.

The safe steering is also evidenced by the low heat transfer throughout the robot navigation procedure. Cumulative equivalent minutes at 43 °C (CEM_43_) is the recognized metric for assessing thermal dose, which correlates effectively with thermal damage across various tissues^55^. The metric involves the knowledge of thermal exposure history as ${{{{\rm{CEM}}}}}_{43}=\sum t{R}^{43-{{{\mathcal{T}}}}}$, where ∑*t* signifies the summation time over the length of exposure, ${{{\mathcal{T}}}}$ denotes the average temperature during the time interval *t*, and *R* is a bistable constant (*R* = 0.25 for ${{{\mathcal{T}}}} < 4{3}^{\circ }$C and *R* = 0.5 for ${{{\mathcal{T}}}} > 4{3}^{\circ }$C)^56^. In a mockup experiment, we measured the thermal diffusion throughout the entire Phase 1 to Phase 3 process (see Fig. 7D) with a thermal camera module (Lepton 3.5, FLIR, Teledyne Technologies, USA). The results show that the LED-equipped endoscopic module has a peak central temperature around 41 °C, and its corresponding CEM_43_ could reach up to 18.75 within 5 min when in direct contact with the tissue. It should be noted that, for skin and muscle, the acceptable threshold is considered 16^57^. In our experiment, the canal’s temperature rose by no more than 1.3 °C throughout the eardrum inspection process. This is due to the robot’s steerability and motion stability, which enable delicate tip motion for collision avoidance, making it an ideal instrument for complicated ear surgeries that require prolonged operations.

Taking advantage of the rapid robot assembly enabled by the single-tendon design, we modified another ear pick-equipped continuum robot (Robot ①) to perform transcanal foreign object removal (see Fig. 7E, F and Movie S10). The mockup task simulates earwax stuck on the canal wall, requiring the ear pick to perform a scooping motion for object removal. This process utilizes the bending and twisting motions provided by the proposed PPT joint mechanism. As shown in Fig. 7F, the TDCR can be steered to approach the object location and twisted to securely hold the “ear wax” (Blu-Tack reusable adhesive, Bostik, France) for retraction. The result underscores the exceptional manipulability of our robot in confined environments, significantly enhancing the usability of force-related manipulation in miniature continuum robots.

### Endoluminal navigation

To assess the robot’s manipulability across both spacious and constrained anatomical environments, we performed experiments using synthetic phantom models and ex vivo porcine organs (sourced from a local butcher) to emulate endoluminal navigation conditions.

**Fig. 8:**
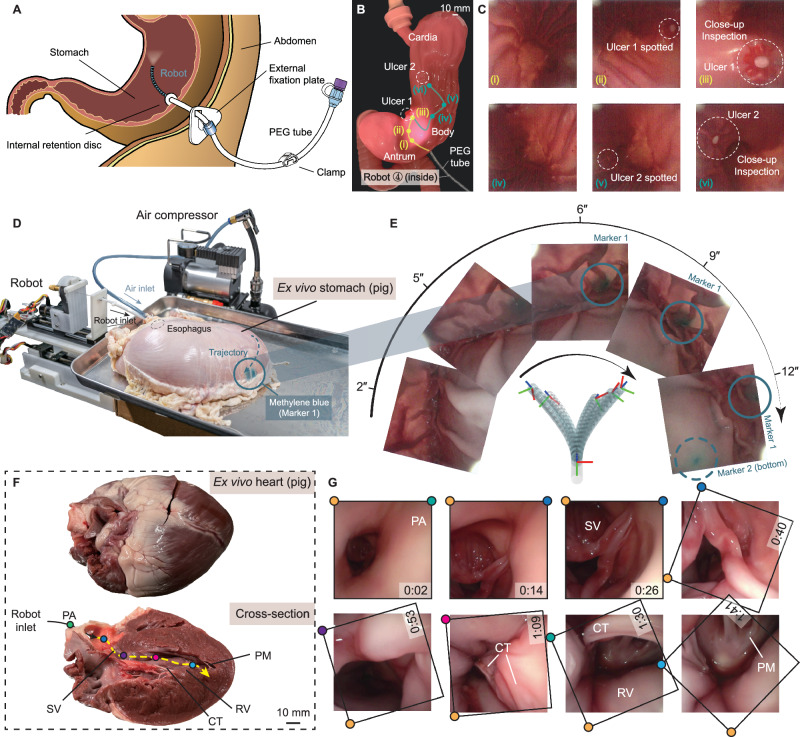
Owing to its miniaturized design and high manipulability, our continuum robot shows strong potential for a wide range of endoluminal tasks. **A** The miniature robot can be used as an omnidirectional endoscope for post-percutaneous endoscopic gastrostomy (PEG) care. Schematics of post-PEG care, illustrating the insertion of an endoscopic continuum robot into the stomach via the PEG tube for intragastric administration. Clinical illustration modified from ref. ^58^. **B** Path of the robot tip in the mockup experiment with a commercial phantom stomach. The stomach contains two gastric ulcers. The navigation path starts from a standard entry incision near the antrum and proceeds toward the cardia. **C** Endoscopic view corresponding to the step-wise path in **B**, showing a clear view of the ulcers. **D** Experiment setup for robot teleoperation on an ex vivo porcine stomach. **E** Two target locations were dyed with a methylene blue injection. The robot can be steered via a distal twisting motion to inspect these targets without causing friction to the esophagus. **F**, **G** The cardioendoscopic robot navigates through an ex vivo porcine heart from PA to the bottom of RV (PA Pulmonary artery, SV Semilunar valve, CT Chordae tendinae, RV Right ventricle, PM Papillary muscle).

To demonstrate the robot’s manipulability in a larger space, we employed an endoscope-equipped robot to navigate an inflated phantom stomach (LM-103, EGD simulator, KOKEN, Japan) in a mockup post-percutaneous endoscopic gastrostomy (PEG) care setting (Fig. 8A). PEG is a medical procedure in which a tube (PEG tube, commonly 6–8 mm in diameter) is inserted into a patient’s stomach through the abdominal wall using endoscopic guidance. This procedure is primarily used to provide a means of long-term feeding (>4–6 weeks) when oral intake and enteral nutrition are insufficient. Once the PEG tube is established, it can serve as a channel for endoscopic inspection if the endoscope is small enough. In a mockup experiment, as shown in Fig. 8B, our miniature TDCR can pass through the plastic tube (4 mm OD) and be inserted into the phantom stomach that contains several gastric ulcers. Thanks to the high manipulability offered by the proposed PPT joint mechanism, the robot demonstrates the ability for large-space maneuvering, navigating from the bottom of the stomach to the cardia location. The operator (a non-clinical student) can easily teleoperate the robot to closely screen for abnormalities (Fig. 8C and Movie S11).

We further validated the robot’s manipulability within a large endoluminal space using an ex vivo porcine stomach. As shown in Fig. 8D, the experimental setup incorporates an air compressor that supplies positive pressure to inflate the stomach via the esophagus. Two target sites, both located along the greater curvature, were marked using methylene blue injections, with one positioned laterally and the other at the base. During the procedure, the robot was introduced through the esophagus and actively steered to visualize the dyed targets. As demonstrated by the end-effector view in Fig. 8E, the robot employed its twisting motion modality to dynamically adjust its forward-looking perspective. Despite its thin diameter of 3.5 mm—significantly smaller than that of state-of-the-art bent-only gastroscopes such as the Olympus GIF-N180 (released in 2024, with a 5.4 mm distal end)—the robot effectively minimized friction between its body and the esophageal wall, which may help reduce patient discomfort, including nausea, during gastroscopic procedures.

To validate the robot’s manipulability within confined anatomical spaces, we evaluated its performance as a steerable cardioendoscopy using an ex vivo porcine heart. Clinical studies demonstrate that cardioendoscopy offers a safer, minimally invasive approach for visualizing intracardiac structures and excising ventricular lesions, enhancing surgical precision while reducing risks^59^. Furthermore, recent advancements have expanded its applications to valve repair and replacement procedures. Endoscopic approaches have been successfully employed for aortic valve replacement and mitral valve repair, demonstrating the versatility and efficacy of endoscopic techniques in addressing complex valvular heart diseases^60–63^. Additionally, cardioendoscopy serves as a valuable adjunct to fluoroscopic imaging in endomyocardial biopsy (EMB), enhancing procedural accuracy and safety^64^. In this experiment, both the robot and the porcine heart were positioned in fixed configurations. As illustrated in Fig. 8F, G, the robot was introduced via the pulmonary artery (PA), traversed the semilunar valve (SV), and reached the base of the right ventricle (RV). During the 100-s navigation sequence, the robot dynamically adjusted its tip orientation to align with the desired lumen when encountering bifurcations in the pathway (Movie S12). This twisting motion was localized to the continuum body of the robot, rather than involving the entire catheter shaft, which may span several meters in a real-world minimally invasive cardiac surgery (MICS). Such localized actuation minimizes friction between the robot and the surrounding “vessels,” potentially reducing mechanical resistance and improving navigational efficiency.

The navigation and inspection results on phantom models and ex vivo porcine organs suggest that our robots and actuation approach are versatile and potentially clinically useful. Its precise steering and flexible operation can assist ongoing improvements in robotic endoluminal navigation.

### In vivo porcine experiments

An in vivo experiment on transurethral bladder procedures was conducted in a 7-month-old female adult pig (98.2 kg, general anesthetized), with ethical approval granted by the Animal Experimental Ethics Committee (AEEC) of The Chinese University of Hong Kong.

For the experimental setup, a sterilized silicone tube (inner diameter 4 mm, outer diameter 5 mm, length 30 cm) was inserted transurethrally to the bladder neck, primarily serving as an artificial channel for the continuum robot. To facilitate smooth insertion, the tube was lubricated and housed a coaxial catheter with a Nelaton tip (10 Fr) for urethral guidance. A flexible endoscope was advanced through this catheter to aid in tube positioning and provide intraluminal stiffness and support. Once the channel was established, the inner catheter and endoscope were withdrawn. A T-shaped connector was then affixed to the exterior end of the channel, with one arm linked to a saline pump for periodic bladder irrigation, and the other serving as the access port for the robot. The continuum robot was also modified for in vivo deployment by encasing its free-end tendon in a plastic sheath (inner diameter 2 mm) to enhance torque transmission. Specifically, our flexible robotic system was evaluated on two bladder procedures: cystoscopy and biopsy.

Bladder endoscopy, or cystoscopy, is a procedure in which a thin, lighted tube with a camera (cystoscope) is inserted through the urethra into the bladder to examine the inner linings. It enables urologists to diagnose and sometimes treat conditions such as bladder cancer, infections, and urinary tract blockages. Commercially available flexible cystoscopes, such as the Olympus CYF-VH HD cysto-nephro videoscope, typically offer bending capabilities of up to 220°/130°. However, they lack the ability to twist along their longitudinal axis. As a result, changing the bending direction requires rotating the entire cystoscope externally, which demands substantial twisting from the operator’s wrist and upper arm and can be particularly challenging for negotiating complex intraluminal geometries.

**Fig. 9:**
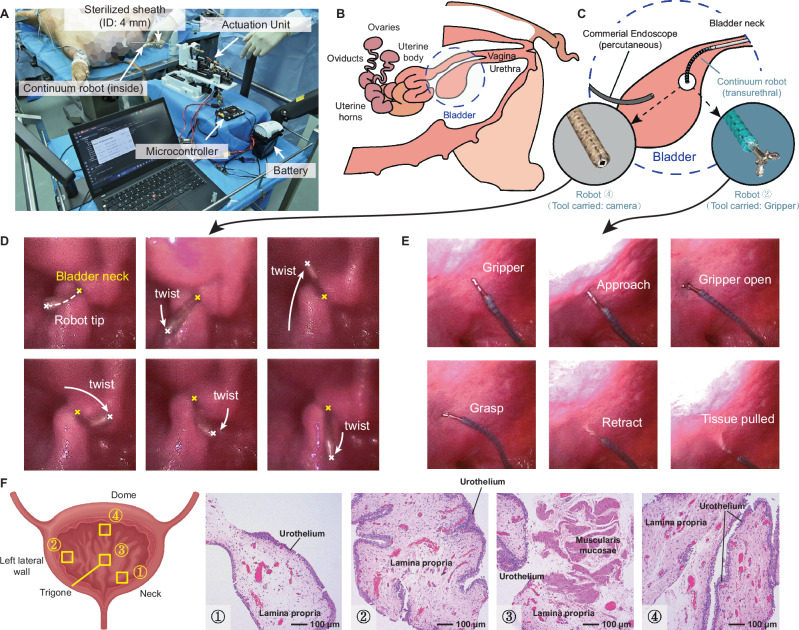
In vivo experiments on a female porcine bladder. **A** Experimental setup. **B** Local anatomy of the bladder and urethra. **C** Illustration of the robot and the third-person observer (endoscope) positioned inside the bladder. **D** Endoscopic view showing the twisting motion of Robot ④ within the bladder. The robot enters through the bladder neck and performs rapid omnidirectional bending intravesically. **E** Endoscopic view of tissue sampling performed by Robot ② on the bladder wall. The robot executes localized tissue grasping using an integrated commercial biopsy tool. **F** Approximate biopsy location in the pig bladder and the corresponding H& E staining results.

In contrast, our PPT tendon joint mechanism enables localized twisting of the continuum body to adjust the position and orientation of the distal tip, eliminating the need for holistic axial rotation (including the sheath) that requires significant twisting involvement from the surgeon and could potentially harm the urethra. In vivo validation was performed using Robot ④, equipped with a camera (OV6946, diameter 1.6 mm). As shown in Fig. 9A–B, the robotic system was introduced through the artificial urethral channel, successfully traversed the urethra, and reached the bladder. The design enables omnidirectional bending of the distal tip (up to 180°) without requiring global axial rotation or multiple tendon actuation (Fig. 9D and Movie S13). Due to its complete waterproof design, the robot successfully performed endoscopy within a bladder filled with urine.

The continuum robot further demonstrates compatibility with other commercial surgical instruments, enabling procedures that require force output, such as biopsy and tissue sampling. As illustrated in Fig. 9C, in addition to the onboard camera, a disposable endoscopic biopsy forceps (“Alligator” jaw type twin-wire biopsy forceps, diameter 2.3 mm, Micro-Tech, China) was integrated at the distal end of the robot (Robot ②). Conventional flexible biopsy forceps typically offer only a single degree of freedom for gripper actuation and lack post-positioning mobility once the target tissue is reached. These limitations are reflected in the motion capabilities shown in the first row of Fig. 9E. Moreover, due to wire-driven actuation, gripper opening often induces unintended bending, complicating precise localization—an essential factor for accurate biopsy procedures. In contrast, our continuum robotic forceps inherently support both bending and twisting motions without compromising gripping performance. As demonstrated in the second row of Fig. 9E, the continuum-based system achieves substantial bending to conform to inclined surfaces on the bladder wall while maintaining sufficient pulling force to extract urothelial tissue (see Movie S13).

To evaluate the biopsy performance comprehensively, specimens were obtained from the bladder neck region, left lateral wall, trigone, and bladder dome region, as depicted in Fig. 9F, and subjected to haematoxylin and eosin (H&E) staining for histopathological assessment. The resulting sections revealed that biopsies obtained via the robotic system successfully captured superficial vesical mucosa, encompassing the urothelium, lamina propria and muscularis mucosae, with a penetration depth of approximately 2–3 mm. This depth is sufficient for accurate tumor staging in non-muscle-invasive urothelial carcinoma. Moreover, the robot’s kinematic dexterity enables on-demand, site-specific sampling across intravesical regions of pronounced curvature, while its axial twisting induces shear stresses that facilitate retraction of the highly compliant bladder wall. Compared with conventional transurethral bladder biopsy and resection instruments^65–68^, our platform achieves comparable biopsy depth and clinical utility. Notably, although the twisting mechanism occasionally induces minor tissue fragmentation, it facilitates dissection from the adherent bladder wall without necessitating repetitive manual wrist or arm manipulation by the surgeon. This capability underscores the potential of the twistable continuum robot to enhance precision, ergonomics, and access in intravesical diagnostic interventions.

## DISCUSSION

Twisting, whether in biological or engineered systems, exemplifies the intersection of mechanics, geometry, and morphology. Biological systems, such as the elephant’s trunks, demonstrate how twistable manipulators maximize the range of workspace and dexterity of motion within a compact dimension. By mimicking these mechanisms, we designed tendon-driven continuum robots that replicate the key principles of twistable tendon joints, introducing omnidirectional continuum robots enabled by a single push–pull–twist tendon. These previously unreported designs and findings provide a foundation for further development of miniature tendon-driven continuum robots, enabling more lifelike, versatile movements and improved manipulability.

### Miniaturization and modularization for tendon-driven continuum robots

Using a single tendon for robot actuation on an eccentric layout significantly reduces the necessary cross-sectional space to accommodate additional tendon channels—which used to be the norm—thus allowing substantial robot miniaturization under the same fabrication conditions and working channel requirements. In an ideal scenario, as illustrated in Fig. 2A, the eccentric cross-sectional layout reduces the robot outer diameter by 15% while preserving the inner diameter of the working channel and maintaining the same thin wall thickness, ensured by the same material strength and fabrication constraints. This approach is particularly beneficial for miniature continuum robots that require a sub-centimeter working channel for additional functions, such as those used in robot-assisted surgeries. Moreover, while offering these merits, the tendon-driven mechanism retains the benefits of high force and torque transmission (Fig. 5E) even after a size reduction comparable to emerging magnetically-driven robots^15,43,44^ (Fig. 2C). The concept of coupling a prismatic (push–pull) and a rotational (twist) joint at the same tendon anchoring point, extending beyond the separated radial and oblique muscle forms in biological systems^23,24,31^, departs from the conventional TDCR actuation paradigm and allows greater compactness in hollow manipulators while providing a practical metric for continuum robot miniaturization.

Another advantage of using a minimal tendon is that it facilitates the rapid assembly and disassembly of the continuum (Fig. 2D), which de facto modularizes the TDCRs. Most existing tendon-driven mechanisms require multiple tendons, cables, or rods to enable spatial motion of the end-effector. This setup complicates the rapid replacement of the manipulator due to the repetitive assembly and detachment actions for each tendon, and most importantly, the co-calibration of the tension. However, leveraging the proposed PPT joint mechanism, we facilitate the rapid replacement of various single-TDCRs without necessitating the co-calibration of multiple tendons. The tension of the only tendon can be readily determined and adjusted through coarse observation of the manipulator’s straightness, thereby obviating the requirement for additional sensors. The enabled swift ‘tool-side’ replacement mechanism can potentially be utilized in robot-assisted biomedical applications, including endoscopic surgery^37^, cell and soft tissue manipulation^69^, and chemical handling^70^, where stringent sterilization and anti-contamination protocols are imperative.

### Manipulability breakthrough via tendon twist

The proposed twisting motion for the tendon joint substantially increases the manipulability of the continuum robot’s tip compared to the traditional bending-rotatable base mechanism. Our theoretical derivation shows that adding a twisting degree of freedom to a single actuation tendon can elevate manipulability, a possibility that, to our knowledge, has not been extensively explored. At the tested configurations, replacing the rotating base with a twistable tendon motion increases manipulability by roughly three orders of magnitude in the poses we examined (Fig. 5D). Note that this improvement is configuration dependent: the manipulability index can fall sharply as configurations approach singularities, so the multiplicative factor reported here applies to major poses and nearby configurations rather than uniformly across the entire workspace. This PPT joint mechanism has the potential to be generalized to scenarios involving multiple tendons in other TDCRs, enabling the assembly of out-of-plane twisting robot motions introduced by the fixed helical routing method^71^, but in a controllable manner. Moreover, incorporating additional DOF into a single tendon joint can potentially reduce the necessity of employing multiple continuum segments, which were previously motivated by spatial motion and distal manipulation. While not yet experimentally validated, our twistable tendon actuation approach is expected to markedly improve manipulability, reachability, and dexterity in multi-tendon continuum systems^72^, enabling more precise operations. Confirming this will require further investigation and should be highlighted as a direction for future work.

Our characterization test of tip lateral force exertion (see Fig. 5E) demonstrates that the twisting-bending configuration retains over 70% of the exertion force compared to traditional bending motion, thereby confirming the robot’s effective manipulability. Additionally, our demonstrative experiments illustrate how the twistable tendon joint provides unconventional motion modalities with enhanced manipulability to miniature TDCRs, enabling their navigation through tortuous environments (Fig. 6A–C), in-hand manipulation through the assembled dual continua-based soft grippers (Fig. 6D–M), and multi-functional flexible otoendoscopes (Fig. 7) within compact robot dimensions. These potential applications highlight new possibilities for TDCRs.

For ease of comparison, Supplementary Table S5 summarizes recent reports of soft and continuum robots that exhibit twistable-body motion. While most studies exploit body twist either passively or through pre-programmed routines, the PPT tendon joint enables twisting through dynamic, runtime configuration; its tendon-driven design also affords high tip-force transmissibility, which is crucial for slender robots operating in confined environments that require effective tip manipulation and low friction (Fig. 4).

### Analytical framework for the morphology and kinematics

This work presents *the first* closed-form analytical framework for modeling the morphology and kinematics of notched tube TDCRs involving a twistable tendon as the continuum robot joint. By assigning local Cartesian frames to the inner centroid of both sides of each notch, we leverage key geometric parameters of the notched tube structure along with beam theory to derive the spatial positions and orientations of these local frames, thereby establishing the robot’s forward kinematics, accounting for the quasi-static robot motion actuated by the pushing, pulling, and twisting of a single tendon. Additionally, by parameterizing key geometric factors within the robot model, this work provides a principled approach to guiding continuum robot design, enabling the engineering of motion ranges tailored to specific application requirements. Moreover, the robot’s inverse kinematics are computed via numerical optimization, which is seamlessly integrated into the simulation environment (see Methodology section). The segmented elements—each corresponding to an identical notch—are sequentially cascaded according to their respective local frames (as illustrated in Fig. 3A), resulting in a complete morphological reconstruction of the continuum robot. Fig. 3C–H systematically compares the morphology predicted by our model with the actual physical appearance of the robot under controlled joint-space inputs. The results validate the model’s high accuracy in predicting both robot morphology and kinematics. To further demonstrate the model’s robustness and generality, the framework was evaluated across various robot lengths and motion modalities. It can be inferred from the results that the proposed analytical framework can be effectively generalized to a broader spectrum of soft robotic systems employing the single-tendon-driven paradigm, thereby enabling a wide range of potential applications.

### Limitations and future steps

As an initial step toward the development of single-tendon-driven continuum robots capable of omnidirectional motion, this work lays the foundation for future investigations, while acknowledging that some important aspects remain beyond the scope of this study due to page constraints.

The first thing should be the continuum robot structures. While our study explores variations in robot length and diameter (Fig. 1H introduces four robots), it does not address non-cylindrical geometries commonly found in other soft continuum robots. For instance, cone-shaped cable-driven designs^73,74^ exhibit pronounced spiral curvatures upon actuation and could benefit significantly from the adoption of our twistable tendon/cable mechanism. Similarly, cone-shaped pneumatic continuum robots, which are widely studied for their ability to produce planar bending motions per segment, typically require multiple units to achieve complex tasks^75^. These systems may also benefit from a twistable joint, potentially realized through innovations in chamber winding patterns that enable adjustable pneumatic chambers for torsional actuation.

The second aspect worth further study is soft material mechanics. Our work focuses on a specific combination of a soft continuum body composed of a photosensitive polymer (see “Robot Design” and “Robot Fabrication and Mechatronics” sections) and a NiTi tendon. However, preliminary experiments suggest that the relative elastic modulus between the continuum body and the actuation tendon significantly influences both morphological and kinematic behavior (see Movie S14). While our model accounts for variations in body stiffness, a comprehensive exploration of how different material properties affect system performance remains an open and promising area for further research.

A third pathway for future investigation involves the hysteresis effects induced by tendon friction and the flexural deformation of the soft body. Due to the selected materials, our prototyped TDCRs exhibit noticeable hysteresis during twisting motions (see Fig. 5F, G). Potential mitigation strategies include employing highly elastic materials for the notched tube continuum body—though this may increase the required actuation torque—and incorporating a hysteresis compensation term into the model. For instance, Wang and Dupont^50^ demonstrated that modeling frictional hysteresis and employing data-driven approaches can significantly enhance the control accuracy of clinical single-tendon cardiac catheters. These mitigation strategies could facilitate the broader use of diverse soft materials as continuum bodies, thereby enabling customization to meet the specific requirements of a wider spectrum of applications.

## METHODS

### Kinematics

#### Notation

The flexible body of the proposed TDCR comprises alternating notched and uncut segments. We investigate the mechanisms underlying four distinct motion modalities: the independent motion of the uncut segments, as well as the bending, twisting, and twisting-bored bending of the notched segments, as detailed in the following sections. Take **R** as an example, the overhead or underlined notation is used for key variables: $\breve{{{{\bf{R}}}}}$ for bending, ${{{{\bf{R}}}}}^{\circ }$ for twisting, ${{{{\bf{R}}}}}^{*}$ for twisting-induced bending and $\underline{{{{\bf{R}}}}}$ for uncut section.

#### Bending

Traditional side-notched tube TDCRs fabricated from NiTi alloys are typically limited to unidirectional bending toward the tendon side^22,76,77^. The combination of NiTi’s superelasticity and relatively high stiffness, along with the notch geometry, renders the structure resistant to deformation in directions other than the intended bending path. In contrast, our 3D-printed continuum robots demonstrate bidirectional bending, including configurations where the tendon side forms the convex (outer) surface of the curve when the tendon is pushed. This behavior is enabled by the material’s capacity to sustain compressive loads without buckling, particularly on the un-notched side. Notably, the bending response is direction-dependent. To characterize this asymmetry, we define two types of bending based on the tendon’s position relative to the curvature: positive bending, when the tendon lies on the concave side, and negative bending, when the tendon lies on the convex side. This bidirectional bending behavior is mathematically described by the neutral bending plane.

**Fig. 10:**
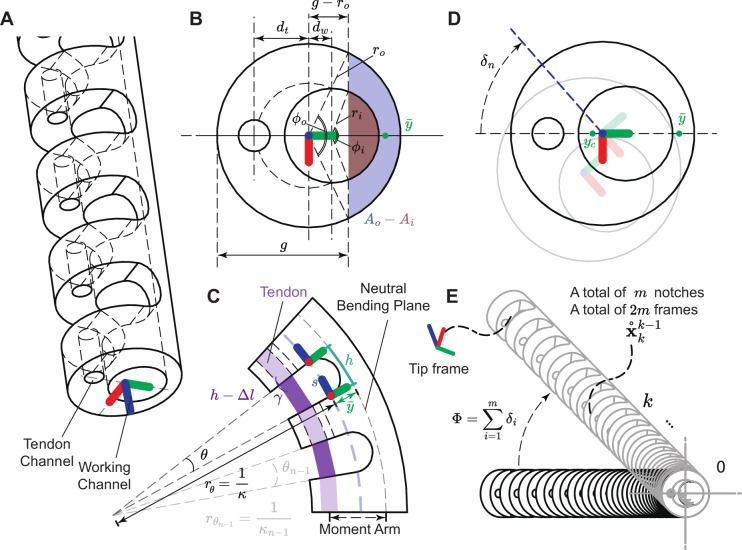
Graphical notation for robot parameters and kinematics. **A** Isometric view: Design of the eccentric notched tube TDCRs—the working channel is eccentric to spare a thicker wall on the tendon side. **B** Cross-sectional view of a notch and its parameters. **C** Side-view of two notches and their parameters in an ideal bending status. **D** Notation of the local twist at the *n*-th notch. **E** Hypothesized resultant twist of the robot with a total of *m* notches.

Following the notations in ref. ^21^, the centroid of the natural bending plane located at the *y*-axis of the local frame is given by the subtraction of two intersecting bounded regions given by

1 $$\bar{y}=\frac{{\bar{y}}_{o}{A}_{o}-{\bar{y}}_{i}{A}_{i}}{{A}_{o}-{A}_{i}},$$

where *A*_o_ and *A*_*i*_ are part of the sectorial areas as shaded in Fig. 10A, B, conforming with *A*_*o*_ ∩ *A*_*i*_ and *A*_o_ > *A*_*i*_; and ${\bar{y}}_{o}$ and ${\bar{y}}_{i}$ are the centroids of the corresponding areas, which can be trigonometrically defined by

$$\left\{\begin{array}{l}{\bar{y}}_{o}=\frac{2{r}_{o}^{3}}{3{A}_{o}}{\sin }^{3}\left(\frac{{\phi }_{o}}{2}\right)\hfill\qquad \qquad 2({{{\rm{a}}}})\quad \\ {\bar{y}}_{i}=\frac{2{r}_{i}^{3}}{3{A}_{i}}{\sin }^{3}\left(\frac{{\phi }_{i}}{2}\right)+{r}_{o}\cos \left(\frac{{\phi }_{o}}{2}\right)-{r}_{i}\cos \left(\frac{{\phi }_{i}}{2}\right)\qquad \qquad 2({{{\rm{b}}}})\quad \end{array}\right.$$

where

3 $${A}_{o}=\frac{{r}_{o}^{2}\left({\phi }_{o}-\sin \left({\phi }_{o}\right)\right)}{2},\,\,\,\,\,\,\,\,{A}_{i}=\frac{{r}_{i}^{2}\left({\phi }_{i}-\sin \left({\phi }_{i}\right)\right)}{2},$$

4 $${\phi }_{o}=2\,\arccos \left(\frac{g-{r}_{o}}{{r}_{o}}\right),\,\,\,\,\,\,\,\,{\phi }_{i}=2\,\arccos \left(\frac{g-{r}_{o}-{d}_{w}}{{r}_{i}}\right).$$

The geometric definition of *ϕ*_*o*/*i*_ is shown by Fig. 10B, which concerns the cutting depth of the notches *g* and the radii of the tubular body *r*_*o*_ and internal working channel *r*_*i*_. Note that the *d*_*w*_ denotes the offset of the eccentric working channel. Unlike existing NiTi-based notched tube TDCRs^26,27^, the eccentric design in our approach mitigates structural failure of thin-walled segments under prolonged tendon loading, thereby enhancing the durability of soft material-based robots. Moreover, the robot’s dimensions can be optimized by tuning *d*_*w*_, subject to controlled parameters such as *r*_*o*_, *r*_*i*_, *g*, and the minimum wall thickness requirement. As a result, the centroid $\bar{y}$ shifts slightly toward the un-notched side, enabling a larger bending angle compared to concentric tubular structures. Detailed derivations are provided in the Supplementary Information. As illustrated in Fig. 10A and Fig. 10C, filleted corners are employed in the cutouts to reduce stress concentrations that are typically introduced by square-cornered designs^30^.

Leveraging the structural advantages of the proposed design, the TDCR also demonstrates enhanced bending capacity due to material compliance. Beyond geometric considerations, a static analysis incorporating material properties is introduced to describe the bending behavior. To this end, we first model the tendon force. The force **F**_*t*_ exerted on the tendon (with initial length *L*) is governed by Hooke’s law:

5 $${{{{\bf{F}}}}}_{{{\mathit{t}}}}=-\frac{{E}_{t}{A}_{t}\Delta l}{L}{{{{\bf{e}}}}}_{z},$$

where *E*_*t*_ is the Young’s modulus of the tendon, *A*_*t*_ is its cross-sectional area, **e**_*z*_ is the unit vector along the *z*-axis in Fig. 10A, and Δ*l* denotes the tendon elastic deformation. Note that although the joint displacement *q*_2_ mostly account for the geometric change of tendon length induced by bending, the assumption here holds that Δ*l* is proportional to *q*_2_ (Δ*l* > 0 for pulling and Δ*l* < 0 for pushing). **F**_**t**_ is either tensile or compressive, depending on the Δ*l,* and acts perpendicular to the cross-sectional plane. This force is transmitted from the tendon to robot’s distal end and induces a total bending moment **M** applied on the robot via the lever arm vector **r** (defined as the distance from the neutral plane to the tendon channel along *y*-axis):

6 $${{{\bf{M}}}}={{{\bf{r}}}}\times {{{{\bf{F}}}}}_{t}=\left(\bar{y}+{d}_{t}\right)\cdot \frac{{E}_{t}{A}_{t}\Delta l}{L}\cdot {{{{\bf{e}}}}}_{x},$$

where *d*_*t*_ denotes the distance from the robotic centroid to the center of the tendon channel; and **e**_*x*_ is the unit vector along the *x*-axis.

The classic Euler-Bernoulli equation for a static linearly elastic beam^78^ establishes the relationship between bending moment and curvature through the bending stiffness *EI*, where *E* is the elastic modulus and *I* is the second moment of area of the beam’s cross-section. However, practical considerations such as friction, force transmission efficiency, and material nonlinearities can compromise the bending capacity. A prominent factor is the asymmetric bending stiffness, leading to unequal bending angles in opposite directions under the same driving force. For positive bending (Δ*l* > 0), the bending action closes the notches, compressing the vacuum volume within the notched section. This compression reduces the effective bending stiffness, thereby allowing greater curvature. Conversely, for negative bending (Δ*l* < 0), the bending action opens the notches, and the 3D-printed body, which is compressed in the notched section, exhibits increased stiffness, resulting in less pronounced curvature compared to positive bending.

To account for the effects arising from the aforementioned factors, we introduce two empirical constants, *ξ*_+_ and *ξ*_−_, for positive and negative bending cases, respectively. We model the bending of the continuum robot using a piecewise constant curvature (PCC) model, assuming each of the *m* notches experiences an evenly distributed reduced moment. Therefore, the bending curvature of an individual notch *n* can be modeled as:

7 $${\kappa }_{n}=\left\{\begin{array}{ll}\frac{2{\xi }_{+}\left\Vert {{{\bf{M}}}}\right\Vert }{m{E}_{s}{I}_{s}}\quad &\,{\mbox{if}}\,\Delta l\ge 0\\ \quad \\ \frac{2{\xi }_{-}\left\Vert {{{\bf{M}}}}\right\Vert }{m{E}_{s}{I}_{s}}\quad &\,{\mbox{if}}\,\Delta l < 0\end{array}\right.$$

where *E*_*s*_ is the Young’s modulus of the soft material, and *I*_*s*_ is the second moment of area of the robot’s cross-section about the neutral bending plane. For the eccentric hollow cross-section depicted in Fig. 10B, the second moment of area should be calculated as:

8 $${I}_{s}=\frac{\pi }{4}\left({r}_{o}^{4}-{r}_{i}^{4}\right)+\pi {r}_{o}^{2}{\bar{y}}^{2}-\pi {r}_{i}^{2}{\left(\bar{y}-{d}_{w}\right)}^{2}.$$

Note that tendon channels are neglected in the calculation of *I*_*s*_, as their dimensions are too small to have a measurable effect. Specifically, in the absence of a working channel, *I*_*s*_ should be evaluated with *r*_*i*_ = 0 and $\bar{y}=0$. With *κ* determined, the arc length *s* and bending angle *θ* per notch are derived from arc geometry as

9 $${s}_{n}=\frac{h}{1+{\kappa }_{n}\bar{y}},$$

10 $${\theta }_{n}={\kappa }_{n}{s}_{n},$$

where *h* represents the notch height.

By assigning two local frames to both ends of each notch, the first frame of an arbitrary notch *n* would be the *k*-th frame from the robot base, where $k=\left(2n-1\right)$ and the second frame to be *k* = 2*n*, and $n=\{1,2,...,(2m+1)\}\in {{\mathbb{Z}}}^{+}$. Then, the bending-induced (⌣) homogeneous transformation matrix of the *k*-th local frame w.r.t. its previous frame can be defined as

11 $${\breve{{{{\bf{T}}}}}}_{k}^{k-1}=\left[\begin{array}{cc}{\breve{{{{\bf{R}}}}}}_{k}^{k-1}&{\breve{{{{\bf{x}}}}}}_{k}^{k-1}\\ {{{\bf{0}}}}&1\end{array}\right],$$

where

12 $${\breve{{{{\bf{R}}}}}}_{k}^{k-1}=\left[\begin{array}{ccc}1&0&0\\ 0&\cos \left({\kappa }_{n}{s}_{n}\right)&-\sin \left({\kappa }_{n}{s}_{n}\right)\\ 0&\sin \left({\kappa }_{n}{s}_{n}\right)&\cos \left({\kappa }_{n}{s}_{n}\right)\end{array}\right],$$

13 $${\breve{{{{\bf{x}}}}}}_{k}^{k-1}={\left[\begin{array}{ccc}0&\frac{-1+\cos \left({\kappa }_{n}{s}_{n}\right)}{{\kappa }_{n}}&\frac{\sin \left({\kappa }_{n}{s}_{n}\right)}{{\kappa }_{n}}\end{array}\right]}^{\top }.$$

Note that Eqs. (11)–(13) valid  ∀ *k* ∈ 2*n*, as we assume the deformations in the notched parts dominate that of the uncut sections. For the uncut sections, i.e., $\forall k\in \left(2n-1\right)$, we neglect their insignificant deformation (compared to the notched sections), such that

14 $${\breve{\underline{{{{\bf{T}}}}}}}_{k}^{k-1}=\left[\begin{array}{cc}{\breve{\underline{{{{\bf{R}}}}}}}_{k}^{k-1}&{\breve{\underline{{{{\bf{x}}}}}}}_{k}^{k-1}\\ {{{\bf{0}}}}&1\end{array}\right],$$

where ${\breve{\underline{{{{\bf{R}}}}}}}_{k}^{k-1}={{{{\bf{I}}}}}_{3}$, and ${\breve{\underline{{{{\bf{x}}}}}}}_{k-1}^{k-2}={\breve{{{{\bf{x}}}}}}_{k}^{k-1}+{[0,0,w]}^{\top }$ with *w* being the uniform width of notched and uncut sections. Here, the underlines indicate the uncut sections.

#### Twisting

The torque applied to the tendon by the copper collet (motorized) is transmitted to the robot via an adhesive bond at the distal end, generating a torsional moment that induces twist along the entire continuum body. Based on the twisted configurations observed in our prototype trials, we adopt a geometric assumption that the overall twist of the continuum robot arises from the cumulative effect of discrete local twists at each notched segment, as illustrated in Fig. 10E. To facilitate modeling, and analogous to a beam under torsion, the twisting angle at each notch, representing an incremental deformation of the overall robot twist, is assumed to follow a linearly increasing profile from the base. Given that the notches are uniformly distributed along the robot body, the local twist imparted by the *n*-th notch relative to its predecessor is defined as *δ*_*n*_ = 2*Φ*/*m*, as depicted in Fig. 10D, where *Φ* denotes the desired tip twist and *m* is the total number of notches.

Due to the eccentric placement of the tendon, the robot twist is inherently non-axial, and this effect becomes more pronounced in longer robots. This phenomenon stems from the mechanical principle that torsional shear stresses can induce bending when stiffness or structural arrangement (including the “lattice” from a metamaterial perspective^79^) is distributed asymmetrically, creating compliance pathways that couple twist and curvature. In a perfectly symmetric structure, concentric torsion produces only shear stresses and uniform twisting about the longitudinal axis, as the center of mass coincides with the center of stiffness. By contrast, when stiffness is distributed asymmetrically—an inherent feature of our geometry and actuation scheme—the structure cannot twist uniformly. The center of stiffness becomes offset from the center of mass or the line of action of the applied load. This eccentricity means that a torsional input (such as our tendon-induced twist) not only generates torsion but also induces bending moments to restore equilibrium. Therefore, for our notched tube TDCRs, each notch unit induces both rotational and translational components, which enables lateral displacement of the robot in addition to pure orientation changes. Accordingly, the homogeneous transformation matrix from frame *k* − 1 to frame *k* is therefore expressed as:

15 $${{{{{\mathbf{T}}}}}^{\circ}}^{k-1}_{k}=\left[\begin{array}{cc}{{{{\mathbf{R}}}}^{\circ}}^{k-1}_{k}&{{{{\mathbf{x}}}}^{\circ}}^{k-1}_{k}\\ {{{\bf{0}}}}&1\end{array}\right],$$

where

16 $${{{{\mathbf{R}}}}^{\circ}}^{k-1}_{k}=\left[\begin{array}{ccc}\cos {\delta }_{n}&-\sin {\delta }_{n}&0\\ \sin {\delta }_{n}&\cos {\delta }_{n}&0\\ 0&0&1\end{array}\right],$$

17 $${{{{\mathbf{x}}}}^{\circ}}^{k-1}_{k}={\left[\begin{array}{ccc}-{y}_{c}\sin {\delta }_{n}&{y}_{c}(\cos {\delta }_{n}-1)&0\end{array}\right]}^{\top }.$$

Particularly, the geometrical centroid of the cross-section of uncut sections *y*_*c*_ w.r.t. local frame *k* (see Fig. 10D can be calculated as

18 $${y}_{c}=\frac{-{d}_{w}{r}_{i}^{2}}{{r}_{o}^{2}-{r}_{i}^{2}}.$$

Additionally, lateral displacement arising from local twist of the robot’s notch alters the effective length along its pre-twisted longitudinal axis, which generates compressive or tensile stresses and subsequently induces bending moments about both the *x*- and *y*-axis. We hypothesize that these norm stresses are proportional to the torsional moment, which acts in a mechanism similar to friction arising from contact pressure. Recall that the torsional moment is also proportional to the twist angle, as established by the torsion equation for circular shafts^78^ as *θ* = *TL*/*GJ*, where *G* is shear modulus, *L* is the length of the shaft, *T* is the applied torque, *J* is the polar second moment of area and *θ* is the angular deflection. Therefore, the resulting bending curvatures on *x*- and *y*-axis can be respectively expressed as:

19 $${\kappa }_{[x],n}={K}_{x}{\sum }_{i=1}^{n}{\delta }_{i},$$

20 $${\kappa }_{[y],n}={K}_{y}{\sum }_{i=1}^{n}{\delta }_{i},$$

where *K*_*x*_ and *K*_*y*_ are empirically determined proportionality constants (to be introduced in the Supplementary Information), *n* denotes the *n*-th notch, and *δ*_*i*_ denotes the local twist of a notch. Since the torsional moment is evaluated based on the cumulative twist from the base, the total sum of twists is used here.

The transformation governing coupled bending about the *x*-axis adopts a formulation ${{{{{\bf{T}}}}}^{*}}_{[x],k}$ similar to that in Eqs. (9)–(13), with parameters adjusted such that *κ*_*n*_ is replaced by *κ*_[*x*],*n*_, and similar substitutions can be applied to other relevant terms (e.g., *s*_*n*_). For coupled bending about the *y*-axis, we define the transformation matrix as:

21 $${{{{\mathbf{T}}}}^{*}}^{k-1}_{[y],k}=\left[\begin{array}{cc}{{{{\mathbf{R}}}}^{*}}^{k-1}_{[y],k}&{{{{\mathbf{x}}}}^{*}}^{k-1}_{[y],k}\\ {{{\bf{0}}}}&1\end{array}\right],$$

where

22 $${{{{\mathbf{R}}}}^{*}}^{k-1}_{[y],k}=\left[\begin{array}{ccc}\cos ({\kappa }_{[y],n}h)&0&\sin ({\kappa }_{[y],n}h)\\ 0&1&0\\ -\sin ({\kappa }_{[y],n}h)&0&\cos ({\kappa }_{[y],n}h)\end{array}\right],$$

23 $${{{{\mathbf{x}}}}^{*}}^{k-1}_{[y],k}={\left[\begin{array}{ccc}\frac{-1+\cos ({\kappa }_{[y],n}h)}{{\kappa }_{[y],n}}&0&0\end{array}\right]}^{\top }.$$

where the local frame *k* satisfies  ∀ *k* ∈ (2*n* − 1). The *z*-axis component is eliminated here because ${{{{{\bf{T}}}}}^{*}}_{[x],k}$ has compensated the displacement along the longitudinal axis needed for the twist-induced bending. In the twisting case, the deformation for the uncut sections is also neglected, as in Eq. (14).

#### Analytic form

Taking the above sections on the bending and twisting kinematics into account, this section summarizes the analytic form of the continuum robot under different configurations.

For the uncut sections, we neglect the insignificant deformation and orientation change; thus, the homogeneous transformation matrix from frame *k* to frame *k* − 1 is described as:

24 $${{{\rm{Uncut}}}}:\quad \quad {{{{\bf{T}}}}}_{k}^{k-1}={\breve{\underline{{{{\bf{T}}}}}}}_{k}^{k-1}.$$

For notched sections, three primary motion modalities are considered: pure bending, pure twisting, and twisting-induced bending. Although the homogeneous transformation matrices derived from the tip frame of the last uncut section to the base frame of the next uncut section can be unified into a single mathematical form, the underlying mechanisms differ, particularly in cases where twisting induces additional bending effects. To ensure clarity, we discuss each modality separately.

In the case of pure bending, no coupling effects are present, and the transformation reduces to the standard bending formulation, as expressed in Eq. (11):

25 $${{{\rm{Bending}}}}:\quad \quad {{{{\bf{T}}}}}_{k}^{k-1}={\breve{{{{\bf{T}}}}}}_{k}^{k-1}$$

In the case of pure tendon twisting, both the axial rotation and the twist-induced bending along two orthogonal axes are considered. This coupling effect results in a more complex transformation, which can be expressed as:

26 $${{{\rm{Twisting}}}}:\quad \quad {{{{\bf{T}}}}}_{k}^{k-1}={{{{{\bf{T}}}}}^{*}}_{[y],k}^{k-1}({\kappa }_{[y],n})\cdot {{{{\mathbf{T}}}}^{\circ}}^{k-1}_{k}({\delta }_{n})\cdot {{{{\mathbf{T}}}}^{*}}^{k-1}_{[x],k}({\kappa }_{[x],n}),$$

In the twisting-induced bending condition, bending along the *x*-axis results from a combination of tendon-driven moments and twist-induced stresses. Specifically, longitudinal tendon forces (either pushing or pulling) generate a primary bending moment, while torsional loading introduces an additional bending component. We model the latter as an additive angular offset to the former. The resulting transformation is expressed as:

27 $${{{\rm{Twisting}}}}-{{{\rm{induced}}}}\,{{{\rm{bending}}}}:\quad \quad {{{{\bf{T}}}}}_{k}^{k-1}=	 {{{{\mathbf{T}}}}^{*}}^{k-1}_{[y],k}({\kappa }_{[y],n})\cdot {{{{\mathbf{T}}}}^{\circ}}^{k-1}_{k}({\delta }_{n})\cdot {\breve{{{{\bf{T}}}}}}_{k}^{k-1}\\ 	 ({\kappa }_{n}+{\kappa }_{[x],n}).$$

This concludes the kinematic analysis.

#### Model limitations and material failure

Extensive characterization experiments on Robot ② were conducted across the full actuation range to evaluate both functional boundaries of the kinematic model and physical limits (Fig. S8). All reported values correspond to actuation-space inputs (motor displacement or rotation). Results demonstrate that the model maintains high accuracy within an actuation envelope, beyond which progressive deviation occurs. For twisting motions, model predictions remain consistent with experimental measurements up to inputs between −204° and 250° (tip rotation ranging from − 209° to 255°), beyond which tip rotation exhibits nonlinear saturation. This nonlinear response arises from geometric stiffening and stress-strain nonlinearities at large torsional deformations, causing the effective torsional compliance to decrease with increasing actuation. For bending motions, model accuracy is preserved up to inputs between -3 mm and 6 mm (tip bending ranging from −61° to 110°). Beyond these actuation thresholds, deviations emerge due to bending plane deviation from tendon compliance, non-uniform material stiffness distribution and pre-twist accumulation. These critical actuation values define the model accuracy boundary, within which kinematic predictions remain reliable for practical applications.

Beyond the model accuracy boundary, our 3D-printed soft continuum robots experience material failure through two distinct mechanisms: adhesive interfaces debonding at the distal tip, and the elastomeric body fracturing. The debonding refers to the catastrophic detachment of the actuation tendon from the continuum body, which occurs at the distal anchoring point under excessive torsional loading (Fig. S8C). This failure mode arises when the applied torque exceeds the torsional compliance limit of the continuum body, resulting in shear stress concentrations at the adhesive-resin interface (603 Retaining Compound, Loctite, USA) that surpass the interfacial bond strength. Extending the anchoring zone into the proximal uncut section with high-performance adhesives can enhance the load transfer capacity and delay the onset of debonding. The body fracturing refers to a progressive material failure through crack initiation and propagation at the notch roots under cyclic tensile loading, particularly during extreme negative bending, where maximum principal stresses develop on the convex surface. Experimental observations reveal that the proximal notch experiences the highest strain concentrations across both positive and negative modes, establishing it as the primary failure location (Fig. S8F). In our experiments, the operational actuation boundary before catastrophic failure extends to ±720° for twist actuation (yielding −368.67° to 370.15° tip rotation) and ±10 mm for bending actuation (yielding −166.77° to 180.14° tip angle), though model accuracy degrades substantially beyond the aforementioned critical points.

Both failure modes exhibit strong dependence on cumulative loading cycles. Newly printed prototypes exhibit substantial damage tolerance and can endure extreme deformation without immediate failure. However, prolonged cyclic loading is expected to (i) plasticize and degrade the adhesive, reducing interfacial load-bearing capacity, and (ii) nucleate and grow microcracks at notch stress concentrators. Progressive accumulation of damage ultimately leads to catastrophic failure once local damage parameters exceed material-specific critical thresholds. The first issue can be mitigated by improving fabrication quality; the second can be addressed by conducting repeated fatigue testing to establish the cycle threshold. These material tests are beyond the scope of this study.

#### Workspace analysis

The theoretical robotic workspaces shown in Fig. 5A–D are computed using the kinematic models derived above (including twisting) and the traditional notched-tube kinematics (excluding twisting)^26^, and are visualized in MATLAB R2022a (academic use, MathWorks). For traditional bending cases (BR, BO), the bending range is constrained to [0°, 180°], and rotation, where applicable, is set to [−180°, 180°]. For the twist-enabled cases (BT, BTR), the maximum positive bending, aligned with the notched side, is set to 180°, while the negative bending is defined as the inverse of the positive joint input, computed via either inverse kinematics or direct forward approximation. Twisting motion is set to [−180°, 180°], with corresponding tendon twist inputs calculated accordingly. This configuration establishes a fair basis for comparing our robot’s workspace with that of traditional designs from a joint space perspective. The resulting joint inputs produce a workspace profile that is not perfectly symmetrical, as illustrated in Fig. 5A–D. For each case, a total of 100,000 uniformly distributed random joint inputs are sampled.

The 2D projections of the point-cloud-based workspace are generated through orthogonal projection onto the canonical planes (i.e., *X-Y* and *Y-Z* planes). To extract the workspace boundary from the scattered point cloud, we first identify the convex hull or alpha-shape boundary of the projected points in each plane. For cases with complex, non-convex workspace geometries (e.g., BT configuration), the boundary is refined by splitting the point cloud into upper and lower regions, computing separate boundaries, and merging them by identifying and excluding intersection regions using polygon intersection operations. The boundary vertices are then sorted sequentially to form a closed polygon.

The surface area of the shell-like 3D workspace was quantified directly from the scattered point cloud using an alpha-shape-based surface reconstruction workflow implemented in MATLAB. The characteristic radius for triangulation was estimated from the point cloud using critical Alpha function. An alphaShape object was then constructed and its boundary facets extracted via boundaryFacets to yield the triangulated open shell. Spurious bridge-like triangles arising from sparse sampling were suppressed by rejecting any triangle whose longest edge exceeded a threshold. The final surface area was obtained by summing the areas of the retained triangles. Workspace coverage is quantified by calculating the percentage ratio between different actuation modes.

#### Simulator

The graphical kinematic simulator is developed based on our previous open-source work^48^ using MATLAB R2022a (academic use, MathWorks). The core of this simulator is to define a series of discrete 3D coordinates representing the robot centroid. First, the target TDCR with *m* notches is segmented into pieces (.stl file) containing a notch and a “disk” (Fig. 2A). Then, a local frame is assigned to the cross-sectional centroid, ensuring the *z*-axis is perpendicular to the disk’s surface following the right-hand rule. Consequently, each segmented piece processes two local frames. For a TDCR with *m* notches, a total of (2*m* + 2) local frames are assigned (including the base frame and the tip frame). Based on the kinematic model, the homogeneous transformation matrix of each local frame (**T**_local_) can be kinematically computed with the given instantaneous joint input (**q**). Finally, the segmented pieces are spatially aligned with the computed local frames. For the inverse kinematics, we utilize the numerical optimization method^48^ that minimizes the cost function:

28 $$\arg {{\min }_{{{{\bf{q}}}}}}\parallel {{{{\bf{P}}}}}_{{{{\rm{task}}}}}-{f}_{{{{\rm{FK}}}}}\left({{{\bf{q}}}}\right){\parallel }_{2}+\parallel \Delta {{{\bf{q}}}}{\parallel }_{2},$$

subjected to the customized constraints for different TDCRs, including the upper and lower bounds of the joint motion. A wide range of optimizers can be selected, and we opt for the fmincon solver due to the rapid convergence and accuracy.

### Robot fabrication and mechatronics

#### 3D-printed TDCRs

The continuum body is made from a digital blend of VeroClear rigid polymer and Agilus30 rubber-like polymer using a PolyJet 3D printer (J826 Prime, Stratasys, USA). The Young’s modulus of VeroClear is about 2–3 GPa, and the approximate Young’s modulus of Agilus30 is generally estimated to be in the range of 0.5–1.5 MPa, which is much softer than most engineering plastics and closer in stiffness to soft biological tissues like skin or relaxed muscle. By controlling the digital mixing ratio of these two materials, we can fabricate soft continuum bodies with customized rigidity. The printer can print a batch of continuum robots (up to 60) in one go within 2 h (Movie S15 and Supplementary Information). To ensure the completeness of the miniature notched tube structure, we select heavy support material (SUP705/SUP706, Stratasys, USA) to wrap up the printing pieces. These support materials can be primarily removed by physical scraping and water jet, then resolved in an alkaline solution overnight. In our work, we opt to immerse the printed parts (wrapped by the support materials) in a sodium hydroxide (NaOH) aqueous solution (5% w/v) for 24 h. This process can sufficiently clear the support materials. Then, the wet TDCRs are dried under ultraviolet (UV) light in a 60 °C drying chamber (Form Cure, Formlabs, USA) for 30 min. The only assembly appears to be gluing (Ergo 5400, Kisling AG, Switzerland) the tendon tip to the robot tip after passing the tendon through the tendon passage.

#### Actuation unit

Regarding the actuation unit, each joint is driven by a brushless direct current (BLDC) gear motor (M2006 P36, RoboMaster, DJI, China), which is affixed to the 3D printed housing as shown in Fig. 1E, and each motor is connected to a capstan via a flexible coupler. The rotational joint is responsible for tendon twists by a pair of synchronous pulleys. For this prototype, we select a 60-tooth driven pulley (2GT-60T, FitSain) and a 20-tooth capstan (2GT-20T, FitSain, China), and locate them 90 mm apart with their axes parallel to each other for a 1:3 speed reduction. A 10-mm width gear belt (2GT-260, FitSain, China) is used for transmission. The tendon is fastened to an anchoring point along the axis of the driven pulley with a copper collet. Additionally, each motor is connected to a BLDC speed controller that is linked to the same microcontroller (Development Board Type-A, RoboMaster, DJI, China) via the Controller Area Network (CAN) bus protocol. The actuation unit is powered by a 22.8 V DC battery (TB48, DJI, China).

#### Communication system for teleoperation

To facilitate characterization, two teleoperation modes—joypad remote control and graphical user interface (GUI) control—are developed. For the remote control, a remote controller (2.4 GHz frequency band, 1 km communication distance, DJI, China) and a wireless receiver (DR16, DJI) for drones are used for teleoperation. As indicated by Fig. S2, three channels are used, namely, for (1) tendon push–pull, (2) tendon twist clockwise (CW) and counterclockwise (CCW), and (3) robot base feeding forward and backward. In this mode, the robotic system can be teleoperated solely without a host computer.

For the GUI-based control, numerical input for those three channels can be assigned to the microcontroller through a serial link connection. The interface (Fig. S3) is based on the TKINTER package and comes with scroll bars and float inputs. Besides, discrete joint motions (${{\mathbb{R}}}^{{{{\mathcal{N}}}}\times 3}$) can be fed to the system through the serial link at a 115,200 Baud rate for quasi-static continuous task space motion. In any event, the robot’s motion can be programmed for the desired motion in the joint space and task space.

### Experiment environment setup

#### Force measurement

To measure the interaction force in Fig. 5E, a force/torque (F/T) sensor (Nano 17, ATI Industrial Automation, USA) was mounted on a manually rotatable stage. For pure bending motions, the continuum robot was horizontally aligned with the F/T sensor. In contrast, for twist-bending motions, the sensor base was rotated to match the robot’s pre-defined twist angles, ensuring maximum contact area.

Data acquisition was performed using a dual-channel setup. Tendon displacement commands were issued via custom Python scripts, while F/T measurements were recorded at 67.5 Hz using the DAQ interface (ATI Industrial Automation, USA). The tendon actuation followed a quasi-static loading protocol, with displacements incremented in 0.25 mm steps up to 2 mm. Each step was followed by a 2-second pause to allow mechanical stabilization, during which real-time F/T readouts were monitored to confirm equilibrium. The distance between the sensing reference frame and the robot tip was calibrated such that contact was initiated at a tendon displacement of 0.5 mm for both motion patterns. Force output was quantified using the Euclidean norm of $F=\sqrt{{F}_{x}^{2}+{F}_{y}^{2}+{F}_{z}^{2}}.$

#### Optical measurement

The optical tracking system used in Fig. 3B comprised a binocular infrared camera (fusionTrack250, Atracsys LLC, Switzerland) paired with a custom-designed tetrahedral fiducial marker weighing 0.22 grams. This marker consisted of three retro-reflective spheres mounted on a 3D-printed resin frame (Fig. S4). Leveraging the known geometry of the marker, the posture of the robot tip at any given time point was computed by applying rigid-body transforms between its current and initial configurations (see Supplementary Information).

During experiments, the marker’s origin coordinates *P* and rotation matrices *R* were recorded at 120 Hz using the fusionTrack Windows interface, while robot actuation was controlled via Python-based serial communication. For kinematic trials, data collection was terminated immediately upon motor stoppage to eliminate hysteresis effects. In contrast, for hysteresis characterization, data acquisition continued for a fixed duration to capture the full hysteresis response. Prior to each trial, the system was reset to minimize pre-twist and pre-tension, thereby ensuring kinematic accuracy.

### Evaluation metrics

The tip positional error $\tilde{P}$ and orientational error $\tilde{\theta }$ between the optical measurements [**P**_*e*_, **R**_*e*_] and the theoretical model predictions [**P**_*m*_, **R**_*m*_], as shown in Fig. 3C–H, were computed as follows:

29 $$\tilde{P}=\left\Vert {{{{\bf{P}}}}}_{e}-{{{{\bf{P}}}}}_{m}\right\Vert,$$

30 $$\tilde{\theta }=\arccos \left(\frac{{{{\rm{Tr}}}}\left({{{{\bf{R}}}}}_{e}{{{{\bf{R}}}}}_{m}^{\top }\right)-1}{2}\right).$$

The root-mean-square error (RMSE) over *N* samples was computed as:

31 $${{{{\rm{RMSE}}}}}_{P}=\sqrt{\frac{1}{N}{\sum }_{i=1}^{N}{\tilde{P}}_{i}^{2}},\quad {{{{\rm{RMSE}}}}}_{\theta }=\sqrt{\frac{1}{N}{\sum }_{i=1}^{N}{\tilde{\theta }}_{i}^{2}},$$

### Reporting summary

Further information on research design is available in the Nature Portfolio Reporting Summary linked to this article.

## Supplementary information

Supplementary Information

Description of Additional Supplementary Files

Supplementary Movie 1

Supplementary Movie 2

Supplementary Movie 3

Supplementary Movie 4

Supplementary Movie 5

Supplementary Movie 6

Supplementary Movie 7

Supplementary Movie 8

Supplementary Movie 9

Supplementary Movie 10

Supplementary Movie 11

Supplementary Movie 12

Supplementary Movie 13

Supplementary Movie 14

Supplementary Movie 15

Reporting Summary

Transparent Peer Review file

## Source data

Source Data

## Data Availability

The data generated in this study are provided in the Supplementary Information and Source Data file. Source data are provided with this paper.

## Code availability

The codes that support the findings of this study are available at https://github.com/yanjunliu-ac/sorotwist.
