# Supplementary material for: Single twistable tendon-driven continuum robots
Source: Nat Commun. 2026 Jul 4;17:8322. doi: 10.1038/s41467-026-74225-3 (PMC13469136; doi:10.1038/s41467-026-74225-3)
Supplement: Supplementary file 2 [file 41467_2026_74225_MOESM19_ESM.pdf]

nature portfolio

Peer Review File

Single twistable tendon-driven continuum robots

Corresponding Author: Professor Hongliang Ren

This file contains all reviewer reports in order by version, followed by all author rebuttals in order by version.

Version 0:

Reviewer comments:

Reviewer #1

(Remarks to the Author)
This manuscript presents a highly innovative and interesting advancement in the field of continuum robotics. The proposed single-tendon Push-Pull-Twist (PPT) actuation paradigm is an elegant solution to the long-standing trade-off between miniaturization, dexterity, and functionality. The comprehensive work, spanning from structure design and analytical modeling to extensive experimental validation including compelling demonstrations, is very impressive. The paper is well-written, clearly structured, and the results are promising for medical and industrial applications. The following comments are intended to further strengthen the manuscript by addressing several key issues that need deeper investigation and discussion.

1. The motivation of using pull-push-twist mechanism is not fully convincing to me. In this mechanism, two motors (rotational joint and prismatic joint) are required, as shown in Fig 2 A, to achieve bending, twisting or their combination, as shown in Fig.2 C. In my opinion, this mobility can be simply achieved by one prismatic joint (the same as the one used by the authors) along with a rotating base. In other words, we don't twist the tendon, instead, we rotate the base of the robot. This pull-push-rotating mechanism has a single tendon and two motors, which are the same as the pull-push-twist mechanism presented by the authors, but, it result in very simple modeling and control methods.

2. The kinematic model demonstrates impressive predictive accuracy, yet its physical underpinnings could be further elucidated to enhance its scientific contribution. Specifically, the model introduces empirical constants (Kx, Ky) to represent the twist-bend coupling without a detailed mechanical explanation of how torsional forces translate into bending within the asymmetric soft structure. Supporting this empirical formulation with a discussion of the underlying mechanics, perhaps validated by a preliminary Finite Element Analysis, would shift the model from a descriptive tool to an explanatory one. Furthermore, the methodology for calibrating these constants (ξ±, Kx, Ky) and their generalizability across different robot dimensions or materials remains unclear. A discussion on these aspects is crucial for establishing the model's broader utility as a predictive design tool.

3. The introduction of torsion, while a key innovation, brings unaddressed questions regarding mechanical stress and structural stability. Applying torsional loads generates internal stresses that are not quantified in the manuscript. For potential surgical applications, an analysis of these stresses is critical to define safe operational limits and prevent material failure. Moreover, the manuscript notes that longer robots exhibit "significant deviation" under pure torsion, which may be linked to the absence of specialized torsional reinforcement in the structural design. A deeper investigation is needed to determine whether this behavior is a stable, predictable deformation or a precursor to torsional buckling, thereby defining the reliable operational envelope of the device.

4. The complex motion of the single tendon, involving simultaneous translation and rotation, likely introduces significant friction and hysteresis that are not addressed in the current work. This is a critical omission, as hysteresis can severely degrade open-loop positioning accuracy and repeatability, which are vital for precision tasks. The manuscript would be substantially strengthened by a quantitative characterization of the system's hysteresis under various actuation modes (bending, twisting, and combined). A subsequent discussion on the impact of these effects on control and potential mitigation strategies would provide a more complete picture of the system's real-world performance limitations and future research directions.
BTW, I think the issues mentioned in comments 4 and 5 can be eliminated by the pull-push-rotating mechanism mentioned in comment 1.

5. The manuscript presents the large hollow core as a primary justification for the single-tendon approach. However, this framing may understate the true innovation of the PPT mechanism. A large internal channel is fundamentally a feature of the robot's cross-sectional geometry—a design parameter tunable in any continuum robot—rather than a unique outcome of using a single tendon. The core contribution of this work appears to be the radical simplification of the actuation scheme, which unlocks unprecedented dexterity and maneuverability at a micro-scale. The resulting hollow space is a valuable consequence that facilitates functional integration, but the primary breakthrough is the new realm of manipulation capabilities. Clarifying this hierarchy of contributions would better focus the narrative on the most impactful aspect of the design.

6. A critical aspect of teleoperated surgical systems is the human-robot interface, particularly concerning visual feedback. The "Twist" actuation, while mechanically powerful, causes the integrated camera's view to rotate along with the robot's body. This camera roll can be extremely disorienting for a human operator, making precise manipulation difficult and increasing cognitive load. This is a well-known challenge in steerable endoscopes. The manuscript would be significantly strengthened by acknowledging this issue and discussing potential solutions, such as software-based image de-rotation to stabilize the visual frame for the operator, which is often a prerequisite for clinical adoption.

(Remarks on code availability)

Reviewer #2

(Remarks to the Author)
The paper presents a new and useful morphology for a TDCR with a single tendon capable of twist and push pull bending capabilities. The authors also offer several insights and innovations related to this design, such as using eccentric tendon/tool channel placement to improve tool channel/OD ratios, kinematics for combined bending and twisting with a single tendon, and several mock up experiments demonstrating the utility of such a device, particularly in medical applications. Overall, the paper provides novel and innovative work in the area of tendon driven continuum robotics with thorough experimental validation of the device. At times, the authors claimed contributions seem to require additional contextualization. Majorly, the work by Tummers in a Cosserat Rod Model for Concentric Push-Pull Robots also includes some work with relative torsion of tubes in what produces similar results as the authors contributions. The discussion should more clearly articulate how this work differs in mechanism and performance with this existing work. Also, claims of increased tool diameter to outer diameter neglect other classes of robots which can achieve even higher results (CTRs, CPPRs, etc). Given this, I am unsure that the contributions of this work extend beyond the continuum robotics and medical robotics specialties.

The main positive aspects of the paper are:
1) The concept of a single, twistable tendon is both interesting and useful. The authors do well to highlight the manufacturing benefits, manipulability benefits, and control benefits with hysteresis analysis.

2) The figures and supplementary videos do an excellent job of explaining device operation, experimental setups, and results. While they often contain a lot of information in one figure, they are able to answer many questions that emerge from the text.

3) The experiments and mock ups generally provide convincing evidence of the potential impact of the device and results. They display good agreement between the models, show the benefits of the device in complex navigation environments. In some of the cases, the benefits of the twisting are less clear than others. The tower provides a good example of capabilites, while some endoscopic uses are less clear in how such a device may be beneficial over a traditional multi-tendon or existing clinical tool which bends and rotates the entire tool. Better clarification of the need for the twisting would make the medical applications even more convincing.

The work would benefit from clarification on a few points:

Some claims seem broad or need to be contextualized. Any claims about miniaturization compared to other robots should include the caveat that the concentric push-pull devices by Tummers, Childs, Oliver-Butler achieve these and even higher ratios of tool channel to OD ratio. The current comparisons are only made to multi-tendon robots or magnetic robots.

The inclusion of twisting induced bending to the robot kinematics is interesting, however it is unclear how much this inclusion improves the modeling results. The presented bending kinematic models are the same as those presented by Webster, Oliver-Butler, and Rothe. It would be good to see the tangible benefits of the authors modeling improvement.

The workspace analysis comes across as comparing the areas of projected workspace volumes. The methods and calculation of these values are unclear, and the comparison of projected area does not provide a complete comparison. I believe there are benefits to the omnidirectional motion, however the benefits are not clearly quantified; presenting volumetric workspace metrics or dexterity indices (e.g., Leibrandt (designing robots for reachability and dexterity)) would make the comparison stronger. While potentially outside the scope of this work, it would be an interesting comparison.

Calculation of the manipulability index is clear in the supplementary text, however the rational behind the claims of 1000 times improvement is unclear to me. It seems intuitive that there will be large improvements to manipulability under this

morphology, but the manuscript lacked justification for that claim.

In regards to force application, the ability of such a device to apply a force in the bending and twisting configuration is not clear. The authors state that it retains 70% of the force capabilites in one orientation but forces will be applied in multiple directions as the tension causes bending in a variety of planes depending on twisting. Force application is only tested in the tip bending plane from what I can tell, and more comprehensive results may give a complete understanding of device capabilities.

Overall, the manuscript presents a promising and well-validated mechanism, but several claims require stronger contextualization against prior work. Addressing these would strengthen the clarity and impact of the contribution and potentially raise the contributions beyond the specialty field.

(Remarks on code availability)

Version 1:

Reviewer comments:

Reviewer #1

(Remarks to the Author)
The authors have done an impressive work on addressing my previous concerns. The addition of the mock intravascular experiment (Figure 5) perfectly justifies the motivation for a twistable tendon over a rotating base. Furthermore, the elaboration on the mechanical principles (center of stiffness offset), operational boundaries, and material failure modes significantly strengthens the scientific rigor of this manuscript. Before final acceptance, I only have a few minor suggestions for the authors to polish the manuscript:
1. Precision in describing hysteresis (Page 19, Line 356): The phrase "tendon-twisting is the primary cause of robot motion hysteresis" is slightly imprecise. Tendon-twisting is the action, whereas the underlying physical causes are likely the tendon-routing friction (amplified during twisting) and the viscoelasticity/residual stress of the soft material. Consider rephrasing to reflect the physical root causes more accurately.
2. Precision in describing manipulability enhancement (Page 34, Line 635): The claim that the proposed mechanism "enhances the robot's manipulability by over 1,000 times" is mathematically accurate based on the provided Yoshikawa index comparison (10e-5 vs. 10e-8). However, presenting this as a strict 1,000-fold improvement may be perceived as slightly exaggerated, as the index drops exponentially when approaching near-singular configurations. Consider softening or contextualizing this claim to reflect the underlying mechanism more rigorously.
3. Typographical and Formatting Errors: While the manuscript has been significantly improved, there are still a few minor typographical and spelling errors that should be corrected prior to publication:
• In the manuscript (Figure 11E, Page 32): In the second sub-figure of the top row, the text label is misspelled as "Appraoch". It should be corrected to "Approach"
• Typographical error in section heading (Page 50, Line 963): The section header "Robot fabrication and mechantronics" contains a typo. It should be corrected to "Robot fabrication and mechatronics"?

(Remarks on code availability)

Reviewer #2

(Remarks to the Author)
Figure 1 does not properly convey the complex shapes that should be produced by the PPT under twist and pull or twist and push. The shapes look comparable to A despite the notches rotating. Potentially including another view would communicate the 3D effects of the actuation.

The addition and emphasis of direct control over distal tip orientation compared to base first bending in friction-heavy environments is compelling and a good addition to the contributions.

In Fig. 5, please label the robots in the figure as well
Thank you for the additional details on the computation of the workspace. The comparison of projected workspaces is still not fully convincing to me. Though the workspace shapes are shell-like structures, the shells take on a surface in the 3D space, and the areas of such surface can be compared, rather than the projection. The projection into 2D of the 3D still does not allow for completely accurate comparison of the workspaces (i.e. additional workspace at the edge of the curve would not correspond to a proportional increase in the workspace area once projected).

Figure 6C, Why is the BTR not able to bend to the same amount as the BR? Clarify this in the caption.

Thank you for the specification on the manipulability. The comparison between 2 DoF and 3 DoF systems leads to an exaggeration of the device benefits regarding manipulability. A comparison with a base rotation, bending, and insertion robot would contextualize the ~1 billion times increase. Alternatively, the comparison to a 3DoF system could be removed entirely,

with a comparison between the 2 DoF systems being the most relevant to this work.

Thank you for the additional experiments and commentary on the loading under bending and twisting.

(Remarks on code availability)

Version 2:

Reviewer comments:

Reviewer #1

(Remarks to the Author)
The authors have thoroughly modified the manuscript to address the reviewers' concerns. I have no further comments. I agree to accept it in its current form.

(Remarks on code availability)

Reviewer #2

(Remarks to the Author)
The authors have nicely addressed all of my comments.

(Remarks on code availability)

Open Access This Peer Review File is licensed under a Creative Commons Attribution 4.0 International License, which permits use, sharing, adaptation, distribution and reproduction in any medium or format, as long as you give appropriate credit to the original author(s) and the source, provide a link to the Creative Commons license, and indicate if changes were made.
In cases where reviewers are anonymous, credit should be given to 'Anonymous Referee' and the source.
The images or other third party material in this Peer Review File are included in the article's Creative Commons license, unless indicated otherwise in a credit line to the material. If material is not included in the article's Creative Commons license and your intended use is not permitted by statutory regulation or exceeds the permitted use, you will need to obtain permission directly from the copyright holder.
To view a copy of this license, visit https://creativecommons.org/licenses/by/4.0/

Response Letter – Paper ID: NCOMMS-25-76163-T/A

SINGLE TWISTABLE TENDON-DRIVEN CONTINUUM ROBOTS

Jiewen Lai[1,†], Yanjun Liu[1,†], Tian-Ao Ren[2], Yan Ma[1], Tao Zhang[1]
Jeremy Y.C. Teoh[3], Mark R. Cutkosky[2], Hongliang Ren[1]

[1]EE, CUHK [2]ME, Stanford [3]CUHK Medical School

{jwlai;hlren}@ee.cuhk.edu.hk

Februray 2026

Acknowledgments & Short Summary

We would like to thank your thorough review of our manuscript and your instrumental comments, which have helped us improve the manuscript for the broader benefit of potential Nature Communications readers. Based on your comments, we have addressed each point with necessary clarifications, amendments, revisions, and results. We would like to invite you to use this response letter as a guide sheet to examine our revised manuscript (NCOMMS-25-76163-T/A) attached.

Color Scheme for this response letter
Red - Reviewers' comment
Blue - Our response & clarification
Green - Our action in the revised manuscript
Brown - Hyperlink

Color Scheme for the revised manuscript
Blue – Revision based on Comments from Reviewer#1
Pink – Revision based on Comments from Reviewer#2

Contents

Reviewer#1 **2**
Comment 1.1 (About the Motivation) 2
Comment 1.2 (Empirical formulation of $\xi_{\pm}$, K_x, and K_y) 6
Comment 1.3 (Safe Operational Limits & Material Failure) 11
Comment 1.4 (Hysteresis) 13
Comment 1.5 (Hollow Robots as the Subject of Investigation) 16
Comment 1.6 (Camera View De-rotation) 18

Reviewer#2 **19**
Comment 2.1 (Contextualization) 20
Comment 2.2 (Modeling Improvement by introducing Twisting) 22
Comment 2.3 (About the Volumetric Workspace) 23
Comment 2.4 (1,000 Times Improvement on Manipulability Index) 27
Comment 2.5 (Force Retention Analysis) 28

Reviewer#1 (See BLUE text in the revised manuscript)

This manuscript presents a highly innovative and interesting advancement in the field of continuum robotics. The proposed single-tendon Push-Pull-Twist (PPT) actuation paradigm is an elegant solution to the long-standing trade-off between miniaturization, dexterity, and functionality. The comprehensive work, spanning from structure design and analytical modeling to extensive experimental validation including compelling demonstrations, is very impressive. The paper is well-written, clearly structured, and the results are promising for medical and industrial applications. The following comments are intended to further strengthen the manuscript by addressing several key issues that need deeper investigation and discussion.

Comment 1.1 (About the Motivation)

The motivation of using pull-push-twist mechanism is not fully convincing to me. In this mechanism, two motors (rotational joint and prismatic joint) are required, as shown in Fig. 2A, to achieve bending, twisting or their combination, as shown in Fig. 2C. In my opinion, this mobility can be simply achieved by one prismatic joint (the same as the one used by the authors) along with a rotating base. In other words, we don't twist the tendon, instead, we rotate the base of the robot. This pull-push-rotating mechanism has a single tendon and two motors, which are the same as the pull-push-twist mechanism presented by the authors, but, it results in very simple modeling and control methods.

Action: In the revised manuscript:

- We include additional examples in the literature review to **clarify the motivation** and inform readers that existing "pull–push–rotating mechanisms" have been reported. The abovementioned works are included in our revised manuscript to further justify the motivation of the PPT actuation paradigm.
- One of the underlying motivations is to figure out a mathematical way to describe the twisting-induced bending deformation: the continuum robot can still bend when we only twist the tendon (See Fig. R3).
- We provide an additional mock demonstrative case in **Figure 5** (see Fig. R3) to justify the motivation and advantage of our PPT tendon joint mechanism **in comparison to the traditional base rotating approach**.
- We include an additional **Table 1** (Fig. R1), which presents a comparative overview of recently reported continuum robots exhibiting/utilizing twistable-body motion. **This table clarifies our motivation and underscores the significance of our approach.**

Please refer to our revision as shown below:

Table 1: Comparative overview of continuum robots featuring twistable-body motion

Ref.	Actuation for Continuum	Mechanism for Body Twisting	3D Manipulability with Twisting Motion	Tip force Transmissibility ↑	Ease of Detachment
Oliver-Butler, *T-RO*, 2019 (*5*)	Tendon-driven	Converging tendon routing	Pre-programmed	High	✗
Russo, *T-MECH*, 2025 (*71*)	Tendon-driven	Helical tendon routing	Pre-programmed	High	✗
Peng, *Nat. Commun.*, 2025 (*40*)	Tendon-driven	Aerial base*	Passive	High	✗
Jones, *Nature*, 2021 (*17*)	Pneumatic-driven	Eccentric wall thickness	Pre-programmed	-	✓
Zhao, *T-MECH*, 2022 (*13*)	Pneumatic-driven	Rotatable base*	-	Medium	✗
Lee, *Nat. Commun.*, 2024 (*33*)	Pneumatic-driven	Mechanical constraint	Pre-programmed	Medium	✓
Zhao, *PNAS*, 2022 (*31*)	-	Twisted LCE ribbon	Surface rolling	-	-
Leanza, *Adv. Funct. Mater.*, 2024 (*23*)	LCE-driven	Helical LCE routing	Pre-programmed	Low	✗
Wu, *PNAS*, 2021 (*32*)	Magnetic-driven	Origami (Kresling unit)	Pre-programmed	Low	✓
Dreyfus, *Sci. Robot.*, 2024 (*18*)	Magnetic-driven	Helical protrusion	Steerable magnetic tip	-	✗
Tummers, *IJRR*, 2025 (*11*)	Push-pull concentric tubes	Helicoidal centroid lines	Dynamically configured	High	✓
**This work**	**Tendon-driven**	**PPT tendon joint**	**Dynamically configured**	**High**	✓

*Not an active continuum body twist in the strict sense.

Figure R1: New Table added to the Discussion section: Comparative overview of continuum robots featuring twistable-body motion.

Cable-driven (soft) continuum robots (CDSR/CDCR) usually employ the *pull-release* mechanism, as soft cables have difficulties in transmitting a pushing force. Therefore, elastic backbones, super-elastic materials, and soft elastomers are generally included in the robot composition (*5*–*7*) so that the pulling-induced elastic energy can restore the robot's shape. For tendon-/rod-driven continuum robots (TDSR/TDCR), an active push on tendons/rods in the axial direction (i.e., the *push-pull* mechanism) can generate visible robot deformation (*8*), since the pushing forces are transmitted to the fixed-end through coaxial mechanical entities. A special case arises when the push-pull rod functions as an internal tube, concentrically aligned within the outer flexible robot (*9*–*11*). Nonetheless, a tendon that is coaxially aligned with the robot's neutral line would produce a 2D motion under the push-pull mechanism, whereas non-coaxially routed tendons are capable of versatile 3D motions despite the routing intricacy (*12*). Employing multiple tendons/cables for omnidirectional motions on TDCR has been extensively studied over the past few decades (*1*). Such well-established conventions have rarely been challenged, except for some bold attempts like introducing a rotatable base (*13*), extensible segments (*14*), and special tendon routing schemes (*5*, *12*), among others. However, due to the need to accommodate sufficient space within the robot manipulator for actuation entities and multiple parallel motorized joints at the proximal end, these steerable robots face challenges in further miniaturization and usually suffer from a large footprint (*1*). To achieve teleoperative spatial motions, multiple sections of these multi-tendon-driven units are required, which further complicates the system's overall design. Flexible robots can exhibit diverse motion modalities (including twisting) and extreme miniaturization (600 um in outer diameter (*15*)) when they take on other forms, such as origami structures (*16*), pre-programmed pneumatic structure (*17*),

Figure R2: The "rotating base" approach is noted in the introduction.

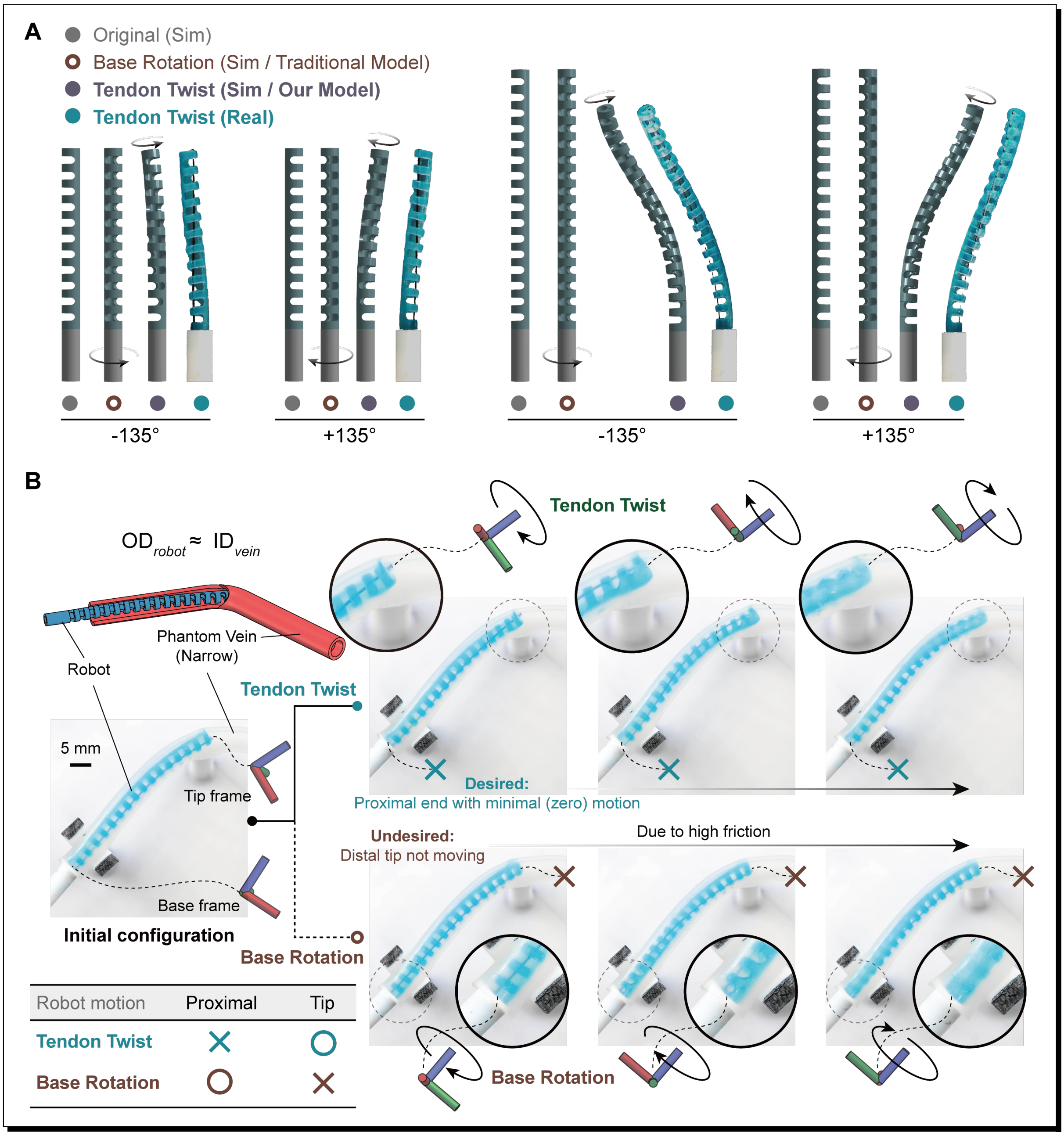

Figure R3: **Action:** We also compare our robot with a conventional "rotating-base" mechanism, with a newly added Figure 5.

Panel A shows that one of the underlying motivations is to figure out a mathematical way to describe the twisting-induced bending deformation: the robot can still bend when we only twist the tendon.

Panel B shows the traditional holistic base/robot rotation model with the proposed PPT tendon joint mechanism. In a mock intravascular operation setting, the robot—designed with a diameter comparable to the vein's narrow inner lumen—demonstrates that tendon twisting can transmit rotation to the functional distal tip with minimal (or even zero) motion at the proximal end (up). In contrast, the traditional (down) of rotating the entire continuum often fails to actuate the distal tip effectively due to high robot–environment friction.

Answer: To answer your questions -

- Utilizing a rotatable base can change the robot shape with respect to a reference frame (such as concentric tube continuum robots Childs and Rucker (2023)), and utilizing both spatial motion and a rotatable base could enable an interesting "null-space-like" motion—where the robot rotates along its curved axis without altering its overall shape from a reference viewpoint. This phenomenon has been explored by Zhao et al. (2022). However, prior works have not explicitly addressed the twist motion of TDCRs (except for helical tendon routing), which could potentially serve a role similar to that of incorporating a rotatable base. Our presented work is trying to fill this gap.

- Continuum robots with side notches can only bend in the direction of the notches—bending on either one side or both side[1]. Therefore, incorporating a rotatable base, or rotating the entire robot, would provide a bending-only robot with 3D maneuverability. **The "rotating base" approach indeed simplifies modeling** (rotate the tip frame w.r.t. the axial direction of the base frame) **and control** (independent base joint, fully decoupled to the bending and can be easily "compensated through joint-level motion" (Dupont et al., 2022)), **and it has been investigated in several prior works** (Simaan et al., 2009).

- Helical tendon routing is commonly regarded as the standard solution for achieving twisting or out-of-plane bending motion in tendon-driven continuum robots (as noted in highly cited review articles (Dupont et al., 2022; Burgner-Kahrs et al., 2015)). **Our results challenge this well-established perception.**

- In this work, we focus on tendon twisting in tendon-driven continuum robots, which has been largely overlooked and undescribed in previous research, to explore how it can be utilized as a potential alternative to base rotation.

[1] See Chitalia et al. (2020), Rothe et al. (2025) and other major works by Prof. Jaydev P. Desai's group

Comment 1.2 (Empirical formulation of $\xi_\pm$, K_x, and K_y)

The kinematic model demonstrates impressive predictive accuracy, yet its physical underpinnings could be further elucidated to enhance its scientific contribution. Specifically, the model introduces empirical constants (K_x, K_y) to represent the twist-bend coupling without a detailed mechanical explanation of how torsional forces translate into bending within the asymmetric soft structure. Supporting this empirical formulation with a discussion of the underlying mechanics, perhaps validated by a preliminary Finite Element Analysis, would shift the model from a descriptive tool to an explanatory one.

Furthermore, the methodology for calibrating these constants ($\xi_\pm$, K_x, K_y) and their **generalizability** across different robot dimensions or materials remains unclear. A discussion on these aspects is crucial for establishing the model's broader utility as a predictive design tool.

Action: In the revised manuscript:

- We include a detailed mechanical explanation of how torsional forces translate into bending within the asymmetric soft structure. A discussion with a ref. Zheng et al. (2025) on metamaterial robotics is made. The revisions are made in the "Methods–Kinematics–Twisting" section, as shown in Fig. R4.

from the base. Given that the notches are uniformly distributed along the robot body, the local twist imparted by the n-th notch relative to its predecessor is defined as $\delta_n = 2\Phi/m$, as depicted in **Fig. 12D**, where Φ denotes the desired tip twist and m is the total number of notches.

Due to the eccentric placement of the tendon, the robot twist (about the tendon axis) is inherently non-axial (about the local notch frame), which can be extremely apparent on longer robots. This phenomenon stems from the mechanical principle that torsional shear stresses can induce bending when stiffness or structural arrangement (including the "lattice" from a metamaterial perspective (79)) is distributed asymmetrically, creating compliance pathways that couple twist and curvature. In a perfectly symmetric structure, concentric torsion produces only shear stresses and uniform twisting about the longitudinal axis, as the center of mass coincides with the center of stiffness. By contrast, when stiffness is distributed asymmetrically—an inherent feature of our geometry and actuation scheme—the structure cannot twist uniformly. The center of stiffness becomes offset from the center of mass or the line of action of the applied load. This eccentricity means that a torsional input (such as our tendon-induced twist) not only generates torsion but also induces bending moments to restore equilibrium. Therefore, for our notched tube TDCRs, each notch unit induces both rotational and translational components, which enables lateral displacement of the robot in addition to pure orientation changes. Accordingly, the homogeneous transformation matrix from frame $k-1$ to frame k is therefore expressed as:

$$\mathring{\mathbf{T}}_k^{k-1} = \begin{bmatrix} \mathring{\mathbf{R}}_k^{k-1} & \mathring{\mathbf{x}}_k^{k-1} \\ \mathbf{0} & 1 \end{bmatrix}, \tag{15}$$

Figure R4: How torsional forces translate into bending within the asymmetric soft structure.

- We add a detailed description outlining the methodology for calibrating the constants $\xi_{\pm}$, K_x, and K_y. Since this calibration process is not among the main findings we intend to emphasize, it is presented in the Supplementary, Section 6, Parameter Calibration (See Fig. R5–R7).

6. Parameter Calibration

The proposed kinematic model incorporates four empirically determined hyper parameters: the bending gains $\xi_{\pm}$ characterizing actuation asymmetry in bilateral bending, and the twist-induced bending coefficients $[K_x, K_y]$ governing coupled deformations along the X- and Y-axes. The former ones quantify the combined effects of tendon transmission, adhesive bonding, frictional losses, and material nonlinearity during push-pull actuation. Although positive and negative bending constitute a single kinematic DOF, their mechanical responses diverge due to distinct notch closure versus opening dynamics, which necessitates independent calibration of ξ_+ and ξ_- as scalar variables. In contrast, K_x and K_y are inherently coupled through the robot's geometric length and internal stress distribution, and are thus treated as a vector in the optimization process.

To identify these parameters, a hierarchical grid search comprising two stages (**Fig. S7A**) is proposed: (1) optimization of the bending gains ξ_+ and ξ_-; (2) optimization of the twist-induced coefficients $[K_x, K_y]$ conditioned on the values from stage (1). In principle, $\xi_{\pm}$ could be calibrated directly from experimental data, assuming pre-characterized material properties E_s and E_t. However, in practice, E_s varies due to fabrication tolerances and environmental factors. To reduce sensitivity, we normalize $\xi_+ = 1$ and calibrate an effective E_s using data from positive bending trials, where tendon actuation occurs on the concave side and notch closure yields more predictable shape. This establishes a reference state for subsequent calibration of ξ_- and $[K_x, K_y]$.

The grid search is implemented with ranges determined by physical constraints and preliminary experiments. In stage (1), ξ_- is searched over $[0, 1]$ with 100 uniformly spaced candidates, using approximately 30 experimental trials comprising 3 distinct configurations with roughly 10 repetitions each (details in **Fig. S5**). The bounded search range reflects the observation that negative bending performance is compromised relative to the opposite situation due to variations in tendon stiffness during push-pull actuation. In stage (2), the twist-induced coefficients are searched over $[0, 0.15]$ for K_x and $[0, 0.05]$ for K_y, yielding a 50×50 candidate grid. These empirically determined ranges are validated using approximately 120 trials spanning 6 twisting and 6 coupled

S7

Figure R5: A hierarchical grid search method were uesd to calibrate the parameteres including K_x, K_y, and $\xi_{\pm}$. This section can be found in the Supplementary in our revised manuscript.

twist-bending configurations, each with roughly 10 repetitions.

The optimization criterion adopts a geodesic distance metric between model predictions and experimental measurements, which is geometrically appropriate for quantifying pose discrepancies on the SE(3) manifold. For a parameter set $\boldsymbol{\theta}$ (either ξ_- or $[K_x, K_y]$), the optimal values are determined by minimizing the mean geodesic distance over the trials:

$$\boldsymbol{\theta}^* = \arg\min_{\boldsymbol{\theta}} \frac{1}{N} \sum_{i=1}^{N} \rho(\mathbf{T}_{e,i}, \mathbf{T}_{m,i}(\mathbf{q}_i, \boldsymbol{\theta})), \tag{S26}$$

where $\mathbf{T}_{m,i}, \mathbf{T}_{e,i} \in \mathrm{SE}(3)$ denote the model-predicted and experimentally measured poses of the robot tip for the i-th data point, respectively; $\mathbf{q}_i$ represents the corresponding actuator inputs; and N is the number of data points. Each pose $\mathbf{T}$ comprises a rotation matrix $\mathbf{R} \in \mathrm{SO}(3)$ and position vector $\mathbf{P} \in \mathbb{R}^3$. The distance ρ combines rotational and translational discrepancies as:

$$\rho(\mathbf{T}_e, \mathbf{T}_m) = \sqrt{\tilde{\theta}(\mathbf{P}_e, \mathbf{P}_m)^2 + \tilde{P}(\mathbf{P}_e, \mathbf{P}_m)^2}, \tag{S27}$$

$$\tilde{\theta}(\mathbf{R}_e, \mathbf{R}_m) = \arccos\left(\frac{\mathrm{Tr}(\mathbf{R}_e^T \mathbf{R}_m) - 1}{2}\right), \quad \tilde{P}(\mathbf{P}_e, \mathbf{P}_m) = \|\mathbf{P}_e - \mathbf{P}_m\|. \tag{S28}$$

In stage (1), negative bending data are used to calibrate ξ_-, yielding a convex error profile (**Fig. S7B**). In stage (2), $[K_x, K_y]$ are calibrated using data from pure twisting and combined twist-bending configurations, resulting in a convex heatmap over the parameter space (**Fig. S7C**). These profiles confirm the convexity of the kinematic model with respect to the hyper parameters, suggesting opportunities for advanced techniques such as adaptive parameter estimation and model-based control. For Robot ①, the calibration yielded $\xi_- = 0.56$, $K_x = 0.0735$, and $K_y = 0.0020$, while Robot ② exhibited $\xi_- = 0.68$, $K_x = 0.0306$, and $K_y = 0.0061$. The consistently small magnitudes of K_y across both instances corroborate the minor influence of coupling-induced bending, as this coefficient characterizes deformations aligned with the primary actuation direction.

The calibrated coefficients are inherently type-specific, influenced by design factors (e.g., length, notch density, wall thickness), material properties, and fabrication inconsistencies. Thus, we suggest executing this calibration protocol for each robot instance. Nonetheless, the hierarchical optimization framework is broadly applicable to diverse continuum robot architectures, albeit with variant parameter outcomes.

Figure R6: Continue to Figure R5

- Our work demonstrates the method's generalizability across two robots (Robot ①–②) with **controlled geometric dimensions** (length/notch number) and with **quantitative confirmation**, representing an initial step toward broader generalization. Robots ③–④, while lacking quantitative results, still leverage these advantages and were used in

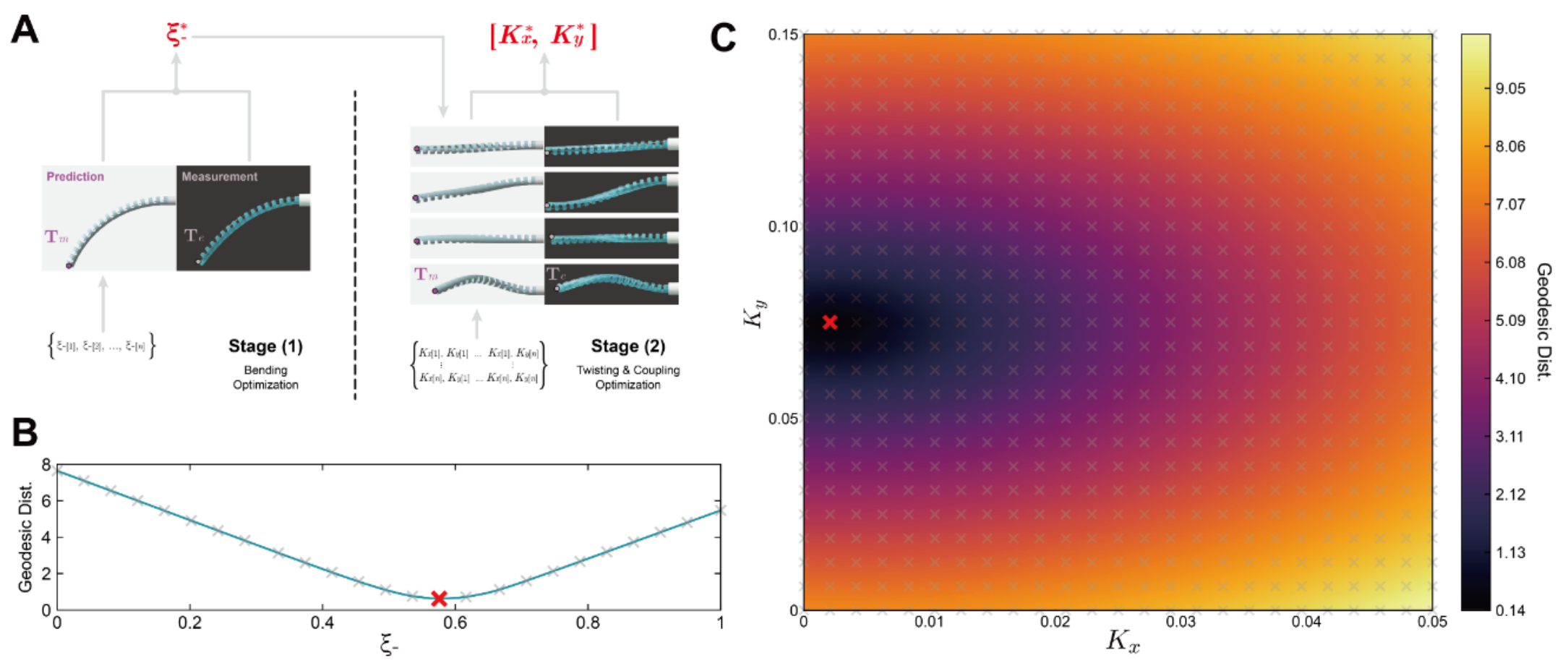

Figure S7: **Hierarchical parameter calibration.** (**A**) Schematic of the optimization workflow. Stage (1) involves fitting the negative bending gain ξ_- using data from pure bending trials; Stage (2) fits the twist-induced coupling coefficients K_x and K_y from twisting and coupled twist-bending trials. (**B**) Error profile as a function of ξ_- for Robot ①, showing a convex profile with a well-defined minimum (red cross). (**C**) Heatmap of the error surface across the parameter space spanned by $[K_x, K_y]$ for Robot ①, showing a convex basin highlighted by the global minimum (red cross).

Figure R7: Additional **Figure S7** in the revised supplementary materials to visualize the calibration process. Corresponding description is given at Page 46.

the demonstrative experiments. Different material types are not yet included; however, we have added a paragraph in the Discussion section (Limitations and future steps) to acknowledge that further investigation will be required to address this aspect (see **Fig. R8**).

- **Movie S14** presents our preliminary observations on how various combinations of tendon materials and continuum robot materials may influence the robot's morphological and kinematic behavior.

The first thing should be the continuum robot structures. While our study explores variations in robot length and diameter (**Fig. 2D** introduces four robots), it does not address non-cylindrical geometries commonly found in other soft continuum robots. For instance, cone-shaped cable-driven designs (*73, 74*) exhibit pronounced spiral curvatures upon actuation and could benefit significantly from the adoption of our twistable tendon/cable mechanism. Similarly, cone-shaped pneumatic continuum robots, which are widely studied for their ability to produce planar bending motions per segment, typically require multiple units to achieve complex tasks (*75*). These systems may also benefit from a twistable joint, potentially realized through innovations in chamber winding patterns that enable adjustable pneumatic chambers for torsional actuation.

The second aspect worth further study is soft material mechanics. Our work focuses on a specific combination of a soft continuum body composed of a photosensitive polymer (see "Robot Design" and "Robot Fabrication and Mechatronics" sections) and a NiTi tendon. However, preliminary experiments suggest that the relative elastic modulus between the continuum body and the actuation tendon significantly influences both morphological and kinematic behavior (see Movie S14). While our model accounts for variations in body stiffness, a comprehensive exploration of how different material properties affect system performance remains an open and promising area for further research.

A third pathway for future investigation involves the hysteresis effects induced by tendon friction and the flexural deformation of the soft body. Due to the selected materials, our prototyped TDCRs exhibit noticeable hysteresis during twisting motions (see **Fig. 6F–G**). Potential mitigation strategies include employing highly elastic materials for the notched tube continuum body—though this may increase the required actuation torque—and incorporating a hysteresis compensation term into the model. For instance, Wang and Dupont (*50*) demonstrated that modeling frictional hysteresis and

Figure R8: Although different material types are not yet included, we have added a paragraph in the Discussion section (Limitations and future steps) acknowledging that further investigation will be required to address this aspect

Comment 1.3 (Safe Operational Limits & Material Failure)

The introduction of torsion, while a key innovation, brings unaddressed questions regarding mechanical stress and structural stability. Applying torsional loads generates internal stresses that are not quantified in the manuscript. For potential surgical applications, an analysis of these stresses is critical to define safe operational limits and prevent material failure. Moreover, the manuscript notes that longer robots exhibit "significant deviation" under pure torsion, which may be linked to the absence of specialized torsional reinforcement in the structural design. A deeper investigation is needed to determine whether this behavior is a stable, predictable deformation or a precursor to torsional buckling, thereby defining the reliable operational envelope of the device.

Action: Applicability of the model — Limits & Boundary: While we are confident in our findings and the proposed mathematical solution, we acknowledge that we have omitted discussion of the operational limits and applicability boundaries of our model. We agree that there is a need to define safe operational limits and prevent material/model failure. Below see our additional results included in the revised supplementary material.

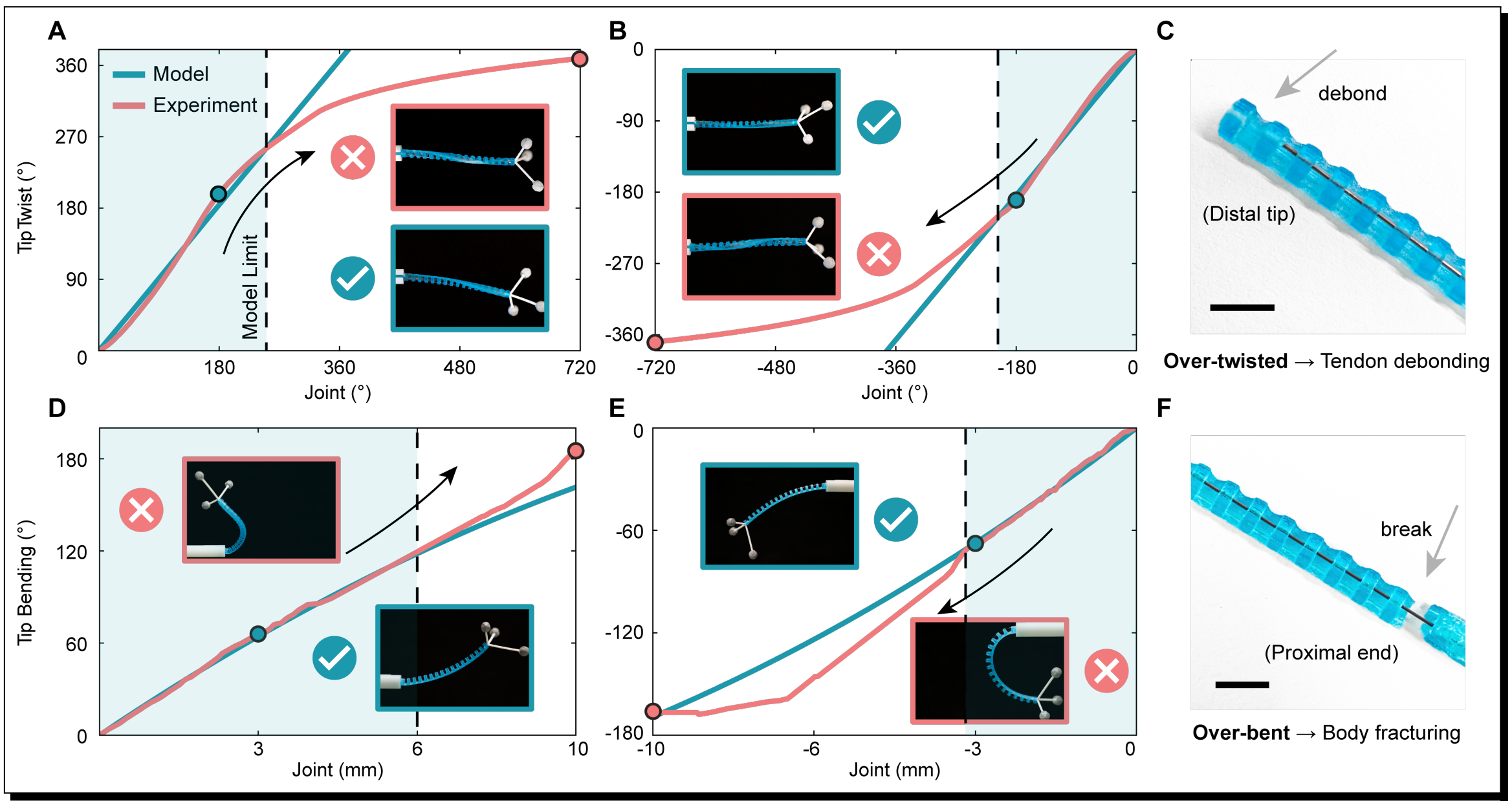

Figure R9: These additional results (**Figure S8**) illustrate the applicable range in which the model can sufficiently predict motion. Panels C and F show extreme cases where the continuum bodies were damaged due to excessive twisting and bending, conditions that may occur after long-term use (6+ months). Due to occlusion of the optical markers under extreme deformation, the complete failure process could not be captured.

Answer: To answer your questions -

- About the mechanical stress: In our manuscript, we did not examine the internal mechanical stress (i.e., the force/torque applied to the tendon). Rather, **our focus was on a kinematic perspective:** figuring out the relationship between tendon joint motion and robot task-space motion by accounting for the continuum robot's geometry and mechanics. The knowledge we present is therefore more readily applicable and reproducible (without an F/T sensor at the tendon joint, it will be easier for readers to replicate).

- About the torsional reinforcement in the structural design: Our work focuses on investigating the unexpected torsional behavior of continuum robots induced by a single tendon twist, and exploring/describing how this phenomenon can be harnessed to achieve more

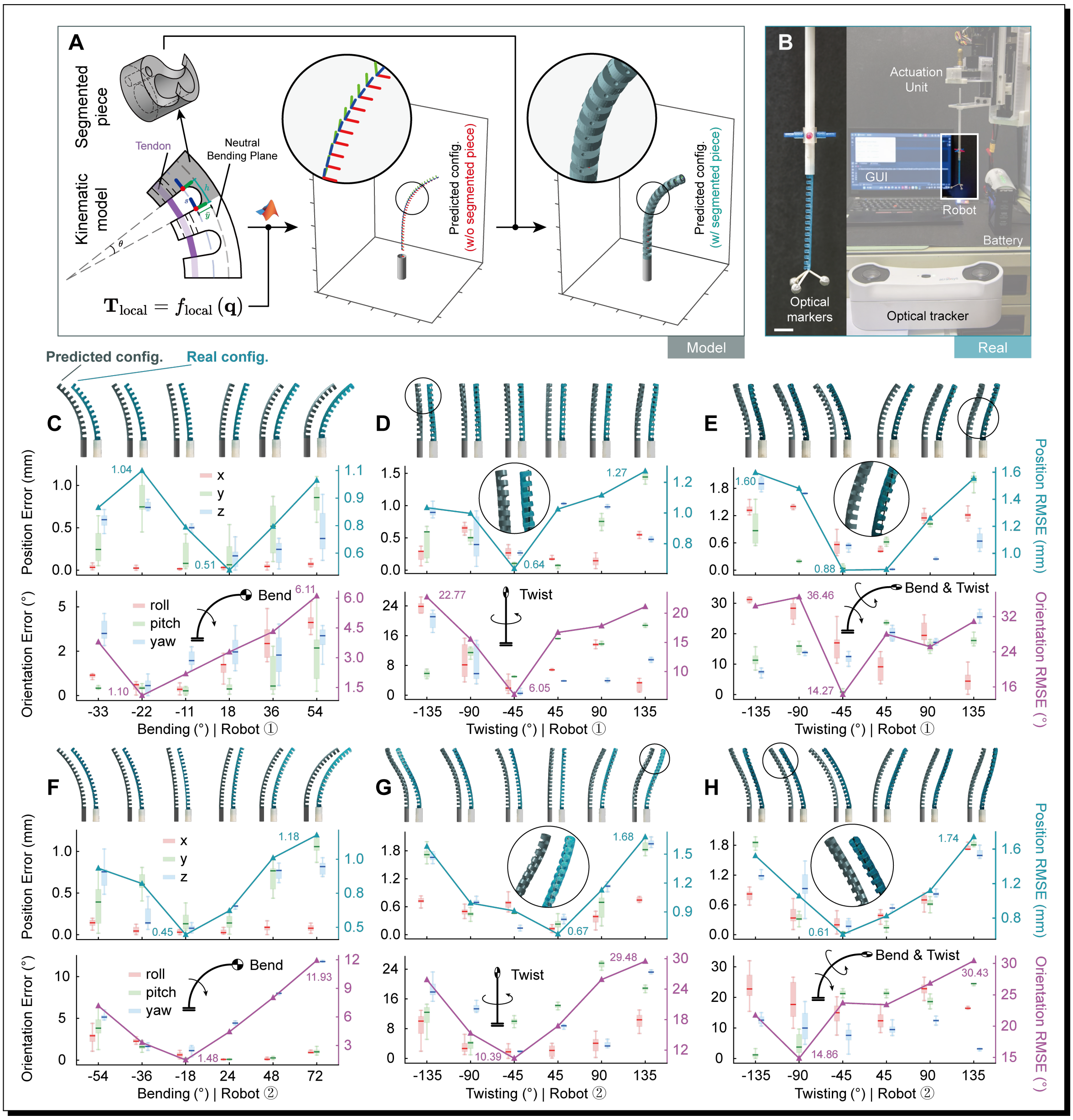

Figure R10: Verification of the newly derived kinematic model on two robots in different lengths (Robot ① with an aspect ratio of 10, and Robot ② with an aspect ratio of 14.29). This is Figure 4 in the manuscript.

complex spatial motions using only one tendon. **Reinforcing the continuum structure (e.g., by increasing its stiffness to resist twisting) would diminish the very twist deformation that we aim to study and utilize.**

- Stable, predictable deformation through modeling: We provide a kinematic description of the continuum robots' deformation. This is supported by quantified verification. As illustrated in **Fig. R10** (**Fig. 4** in the manuscript), we compare the tip position (X–Y–Z) and orientation (R–P–Y) errors between the model-predicted and experimentally measured robot configurations. Quantitative results suggest that our model is not perfect—the error will be enlarged with extreme twisting. Two continuum robots of different lengths were tested to demonstrate the model's generalizability. The results presented in **Fig. 5** and **Fig. 6** also demonstrate that **our investigation offers an explainable solution for achieving stable and predictable continuum robot deformation.**

Comment 1.4 (Hysteresis)

The complex motion of the single tendon, involving simultaneous translation and rotation, likely introduces significant friction and hysteresis that are not addressed in the current work. This is a critical omission, as hysteresis can severely degrade open-loop positioning accuracy and repeatability, which are vital for precision tasks. The manuscript would be substantially strengthened by a quantitative characterization of the system's hysteresis under various actuation modes (bending, twisting, and combined). A subsequent discussion on the impact of these effects on control and potential mitigation strategies would provide a more complete picture of the system's real-world performance limitations and future research directions.

BTW, I think the issues mentioned in comments 4 and 5 can be eliminated by the pull-push-rotating mechanism mentioned in comment 1.

Answer: Thank you for your comments.

- **Action:** The hysteresis introduced by tendon twisting and soft continuum body twisting is noted and addressed in our revised manuscript (Section: Results / Subsection: Workspace, manipulability, and output force performance) — Page 17 (Fig. R11).
- (**Figure 6F**) On Robot ②, we first conducted controlled experiments comparing our approach with two alternatives: (1) the **pull-release** joint motion—a commonly adopted mechanism in many other multi-TDCRs but not used in our design—and (2) the **two-way actuation** (reversible push-pull/positive-negative twist) implemented in our case. The results show that our push-pull mechanism significantly reduces hysteresis in bending, yielding a more symmetrical tip-bending profile.
- (**Figure 6F(iii, iv)**) However, reverse tendon twisting, even when ending at the zeroing position, results in over-twisting tip motion in the opposite direction with substantial hysteresis, whereas the twist-release method results in insufficient reverse motion (i.e., under-twisting), failing to return to the initial robot pose. The over-twisting occurs due to residual stress in the low-elastic soft material, which is subjected to high angular acceleration of the tendon.
- (**Figure 6G**) The repeated trials reaffirm the continuum's hysteresis in tendon twisting. **We show that this hysteresis in continuum twisting is predictable.**
- Since our experiments were conducted only on a specific robot (Robot ②), it is impractical to derive a general mitigation strategy solely from the hysteresis profile of a single robot on an offline basis. Such a strategy would require **sensory feedback** and the development of advanced **closed-loop methods** (typically involving deep learning, e.g., Wang and Dupont (2025); Cho et al. (2024)), which lie beyond the scope of this work. **Action:** A discussion has been updated (see Fig. R11).

Hysteresis arising from tendon friction and flexural deformation is generally observed in tendon-driven notched tube continuum robots (*50*). To evaluate the hysteresis of the proposed PPT joint motion in soft notched tube TDCRs, we conducted controlled experiments comparing it with the actuate-release joint motion, a commonly adopted mechanism in many other multi-TDCRs. Various modes of motion, including bending, twisting, and coupled motion, were examined on Robot ②. The tip bending/twisting angle was computed based on the optically measured tip position (**Fig. 4B**). Specifically, the robot was first bent via a 3-mm tendon pull, then returned to the zeroing joint status via pushing. In the controlled experiment, the tendon was released by manually loosening the copper collet (the anchoring point) after achieving the same bending motion. As shown in the results (**Fig. 6F(i)**), the pull-push joint motion demonstrates a trapezoidal bending deformation profile over time, indicating low hysteresis in the pulling-induced bending. Conversely, the pull-release joint motion exhibits rapid deformation upon tendon release and fails to return to the initial pose. A similar result is observed in the pushing-induced bending case (**Fig. 6F(ii)**). In an active tendon-twisting case (**Fig. 6F(iii)**), the forward and inverse twisting (from 0° to 175°, and from 175° to 0° in the joint space) show a more symmetrical plotting profile than the twist-release convention, suggesting a highly maneuverable angular velocity in robot twisting. However, reverse tendon twisting, even when ending at the zeroing position, results in over-twisting tip motion in the opposite direction with substantial hysteresis, whereas the twist-release method results in insufficient reverse motion (i.e., under-twisting), failing to return to the initial robot pose. The over-twisting occurs due to residual stress in the low-elastic soft material, which is subjected to high angular acceleration of the tendon. A similar result can be observed in the case of bending-twisting coupled motion (**Fig. 6F(iv)**), which confirms that tendon-twisting is the primary cause of robot motion hysteresis. Nevertheless, hysteresis can be easily addressed using closed-loop control methods (*11, 50, 51*), which are beyond the scope of this work. **Figure 6G** presents the corresponding repeated trials (three times) for the results shown in **Fig. 6F**, reaffirming the robot motion hysteresis, particularly when tendon twisting is involved. Additionally, it is observed that tendon pushing can elongate the robot's length under excessive bending (**Fig. 6G(ii)**), resulting in a plateau in the bending angle. This plateau can be overcome by further stretching the tendon. The difference in bi-directional bending aligns with our modeling and workspace analysis (**Fig. 6B–D**).

Figure R11: Discussion on the **hysteresis** in the main text. The discussion is made based on the results from Figure 6.

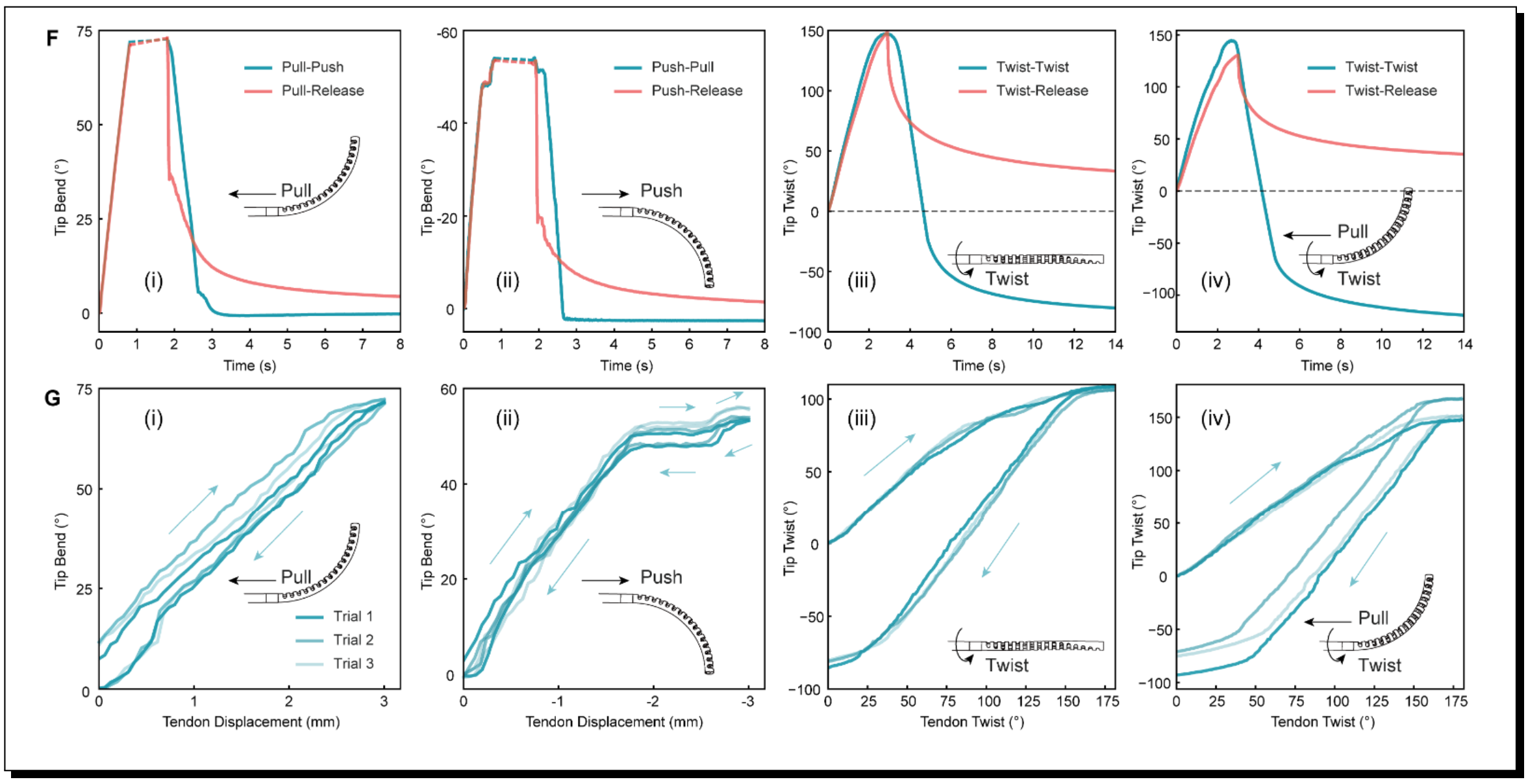

Figure R12: **Above items are Figure 6F and 6G from the manuscript.** Item **(F)** shows the hysteresis tests for different modes of motion, including (i-ii) bending, (iii) twisting, and (iv) coupled motion. We compare the hysteresis effect between the opposite joint motion (ours) and the actuate-release joint motion (a commonly adopted mechanism for other TDCRs), evidencing significant hysteresis in joint twisting in both cases. Item **(G)** shows the hysteresis tests with repeated trials (3 cycles) for different modes of motion, illustrating the joint-tip motion relationship.

Comment 1.5 (Hollow Robots as the Subject of Investigation)

The manuscript presents the large hollow core as a primary justification for the single-tendon approach. However, this framing may understate the true innovation of the PPT mechanism. A large internal channel is fundamentally a feature of the robot's cross-sectional geometry—a design parameter tunable in any continuum robot—rather than a unique outcome of using a single tendon. The core contribution of this work appears to be the radical simplification of the actuation scheme, which unlocks unprecedented dexterity and maneuverability at a micro-scale. The resulting hollow space is a valuable consequence that facilitates functional integration, but the primary breakthrough is the new realm of manipulation capabilities. **Clarifying this hierarchy of contributions would better focus the narrative on the most impactful aspect of the design.**

In this work, we present a bio-inspired, omnidirectional, notched tube tendon-driven continuum robot that utilizes a single push-pull-twist (PPT) tendon joint, designed for miniaturization and enhanced spatial manipulability (**Fig. 1**). The proposed PPT tendon joint mechanism, previously undescribed, fundamentally advances the capabilities of notched tube continuum robots by extending beyond conventional unidirectional and bidirectional bending to enable axial twist and near-helical motion. (**Fig. 1**). Consequently, tendon-driven robots can be dramatically miniaturized by relying on a single tendon, and most importantly, without substantially compromising mechano-conductivity. The eccentrically placed tendon with the twistable motion can significantly improve the manipulability of the distal tip, facilitating a variety of potential applications where robot dimensions are highly constrained. To understand the functioning of the PPT tendon joint mechanism, this study investigates its behavior in representative TDCRs—primarily those with a hollow working channel—focusing on kinematics, performance, and potential applications that could leverage the additional twisting motion it provides (see **Movie 1**). To the best of our knowledge, this is the first time a general kinematic model has been proposed for single-tendon-driven continuum robots featuring PPT tendon joints. We anticipate that this work will inform future studies on tendon-driven continuum structures at a fundamental level, laying the groundwork for advances in design, optimization, control, and application.

6

Figure R13: **Action:** In the revised '**Contribution**' paragraph (immediately preceding the **RESULTS** section), we inform readers that representative TCDRs featuring a hollow working channel have been selected for analysis.

Answer: We agree that our study on the PPT tendon-driven mechanism deserves particular emphasis. The radical simplification of the actuation scheme is a key point for streamlining the narrative. This would enhance the generalizability of our method. We select "representative" TDCRs with an internal working channel, given the following consideration:

- Eccentric notched-tube TDCRs with hollow working channels have been introduced and studied in several well-known works, such as York et al. (2015); Chitalia et al. (2020). Their primary motivation lies in facilitating functional integration, particularly

in biomedical and industrial applications. We adopt this general design to:

1. Maintain alignment with existing research; and,
2. Justify our demonstrative applications that benefit from the newly proposed actuation mechanism.

- We provide the second moment of area I_s in Eq. (8) as

$$I_s = \frac{\pi}{4}\left(r_o^4 - r_i^4\right) + \pi r_o^2 \bar{y}^2 - \pi r_i^2 \left(\bar{y} - d_w\right)^2.$$

  A TDCR without a working channel can be considered as a special case of our presented TDCR, i.e., $r_i = 0$, and $\bar{y} = 0$, such that $I_s = \frac{\pi}{4} r_o^4$. We neglect the tendon channel, which is too small to cause a difference.

We appreciate the suggestion to clarify the hierarchy of contributions, as this will help highlight the potential impact of our work.

Comment 1.6 (Camera View De-rotation)

A critical aspect of teleoperated surgical systems is the human-robot interface, particularly concerning visual feedback. The "Twist" actuation, while mechanically powerful, causes the integrated camera's view to rotate along with the robot's body. This camera roll can be extremely disorienting for a human operator, making precise manipulation difficult and increasing cognitive load. This is a well-known challenge in steerable endoscopes. The manuscript would be significantly strengthened by acknowledging this issue and discussing potential solutions, such as software-based image de-rotation to stabilize the visual frame for the operator, which is often a prerequisite for clinical adoption.

Answer: Thank you for your suggestion. We acknowledge that the camera's view can be rotated under our actuation scheme, similar to other steerable endoscopes that achieve rotation along their flexible bodies. This camera roll may disorient the operator, making precise manipulation challenging and increasing cognitive load. We agree that this issue should be acknowledged.

Action: In the revised manuscript, we inform readers of this issue and outline potential solutions, including an additional reference (van Dijk et al., 2024). Please refer to the updated paragraph below (Figure R14).

> the centers of these passages were not aligned in the same plane, exceeding the robot's workspace capabilities without the ability to twist.
>
> Moreover, from a human–robot interface perspective, twisting motion, though mechanically advantageous, rotates the endoscopic view with the robot's body—similar to other steerable endoscopes that rotate along their flexible shafts—potentially disorienting the operator and increasing cognitive demands. This drawback can be mitigated by applying a derotation (de-rolling) algorithm to the camera view, either by compensating for predictable tip twisting or by using computer vision methods that periodically recognize visual landmarks (*52*). While such approaches may improve the operator's visual orientation, they could also disrupt the sense of teleoperation. The presented experiments did not implement a derotation algorithm, as this lies beyond the scope of the present study.

Figure R14: This additional discussion is added to the revised manuscript. See page 20.

Action: For your reference, we also provide a post-processed video (**Movie R1**) showing the camera view from Movie S4 **with derotation applied**, based on the tip twisting motion data presented in Figure 6. We believe that the original view in Movie S4 provides better guidance for teleoperation than the derotated version in Movie R1.

Reviewer#2 (See PINK text in the revised manuscript)

The paper presents a new and useful morphology for a TDCR with a single tendon capable of twist and push pull bending capabilities. The authors also offer several insights and innovations related to this design, such as using eccentric tendon/tool channel placement to improve tool channel/OD ratios, kinematics for combined bending and twisting with a single tendon, and several mock up experiments demonstrating the utility of such a device, particularly in medical applications. Overall, the paper provides novel and innovative work in the area of tendon driven continuum robotics with thorough experimental validation of the device. At times, the authors claimed contributions seem to require additional contextualization. Majorly, the work by Tummers in a Cosserat Rod Model for Concentric Push-Pull Robots also includes some work with relative torsion of tubes in what produces similar results as the authors contributions. The discussion should more clearly articulate how this work differs in mechanism and performance with this existing work. Also, claims of increased tool diameter to outer diameter neglect other classes of robots which can achieve even higher results (CTRs, CPPRs, etc). Given this, I am unsure that the contributions of this work extend beyond the continuum robotics and medical robotics specialties.

The main positive aspects of the paper are:

1. The concept of a single, twistable tendon is both interesting and useful. The authors do well to highlight the manufacturing benefits, manipulability benefits, and control benefits with hysteresis analysis.

2. The figures and supplementary videos do an excellent job of explaining device operation, experimental setups, and results. While they often contain a lot of information in one figure, they are able to answer many questions that emerge from the text.

3. The experiments and mock ups generally provide convincing evidence of the potential impact of the device and results. They display good agreement between the models, show the benefits of the device in complex navigation environments. In some of the cases, the benefits of the twisting are less clear than others. The tower provides a good example of capabilites, while some endoscopic uses are less clear in how such a device may be beneficial over a traditional multi-tendon or existing clinical tool which bends and rotates the entire tool. Better clarification of the need for the twisting would make the medical applications even more convincing.

Overall, the manuscript presents a promising and well-validated mechanism, but several claims require stronger contextualization against prior work. Addressing these would strengthen the clarity and impact of the contribution and potentially raise the contributions beyond the specialty field.

Comment 2.1 (Contextualization)

Some claims seem broad or need to be contextualized. Any claims about miniaturization compared to other robots should include the caveat that the concentric push-pull devices by Tummers, Childs, Oliver-Butler achieve these and even higher ratios of tool channel to OD ratio. The current comparisons are only made to multi-tendon robots or magnetic robots.

Answer: Thank you for your suggestion. We appreciate this observation and agree that certain statements could benefit from additional context to avoid overinterpretation.

Action: In the revised manuscript, we have clarified these points to ensure that the claims are appropriately framed within the scope of our findings. Please refer to Figures R15–R17.

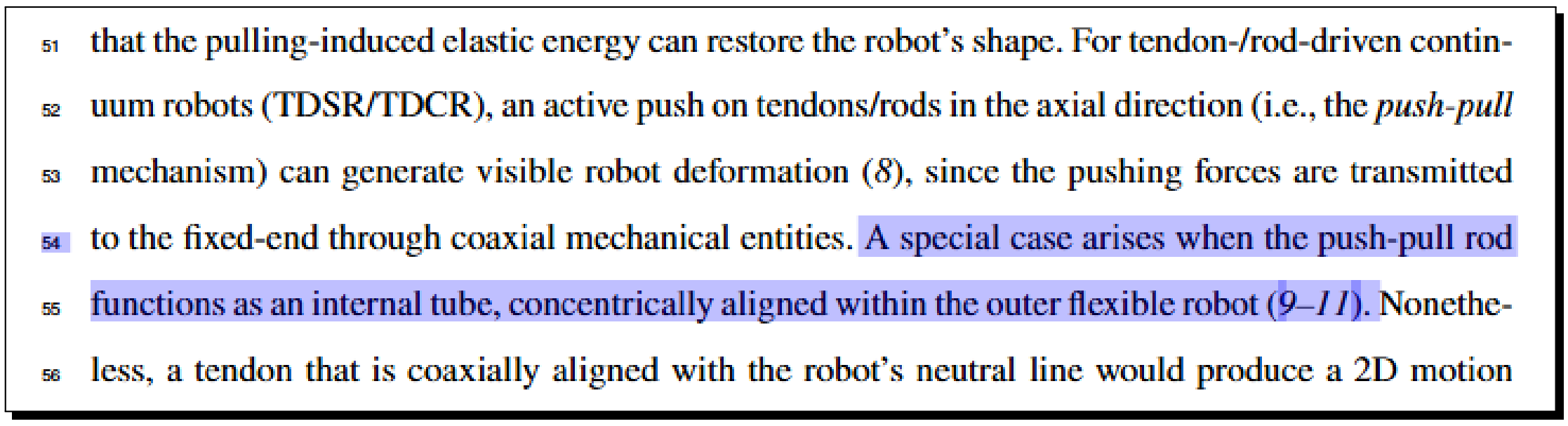
that the pulling-induced elastic energy can restore the robot's shape. For tendon-/rod-driven continuum robots (TDSR/TDCR), an active push on tendons/rods in the axial direction (i.e., the *push-pull* mechanism) can generate visible robot deformation (*8*), since the pushing forces are transmitted to the fixed-end through coaxial mechanical entities. A special case arises when the push-pull rod functions as an internal tube, concentrically aligned within the outer flexible robot (*9*–*11*). Nonetheless, a tendon that is coaxially aligned with the robot's neutral line would produce a 2D motion

Figure R15: We have cited these important works you mentioned in our original manuscript, including [9–11] Childs and Rucker (2023); Oliver-Butler et al. (2021); Tummers et al. (2025).

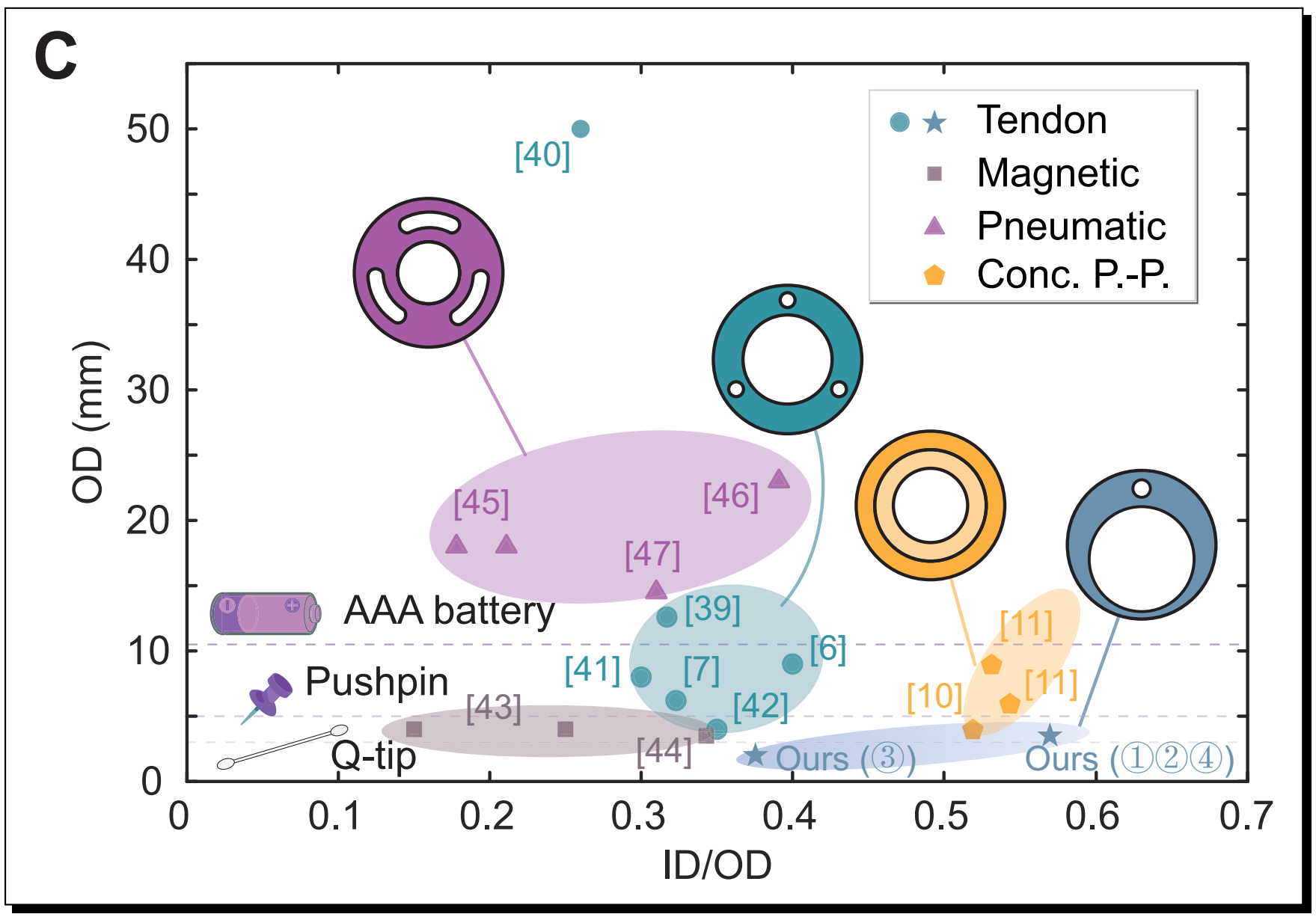

Figure R16: This is the revised Figure 3C, see Page 12 in the revised manuscript. The revised figure includes [11] Tummers et al. (2025) [IJRR] and [10] Oliver-Butler et al. (2021) [TRO]. The revised caption is: **Action:** (C) Compared to other soft continuum robots, our single tendon-driven design achieves one of the highest ID/OD ratios while maintaining an OD comparable to magnetically driven miniature continuum robots.

Adopting single-tendon actuation can reduce the dimensions of TDCRs without compromising the size of the inner working channel under identical fabrication conditions, thereby extending the upper limit of the circumferential hollow (ID/OD) ratio for steerable miniature continuum robots, particularly those fabricated from soft materials. For example, consider a traditional TDCR with three concentrically distributed tendons, having an OD of 5 mm, an ID of 3 mm, and a tendon channel diameter (TCD) of 0.5 mm, as shown in **Fig. 3A(i)**. Its thin wall (TW) thickness would be 0.25 mm. By controlling the OD, ID, and TCD, and employing a single tendon eccentric cross-sectional design, the minimal TW thickness would double to 0.5 mm, resulting in a stronger tendon channel (**Fig. 3A(ii)**). If the fabrication conditions allow for a minimum TW structure of 0.25 mm (regardless of durability), the robot's OD, with the same ID and TCD, would be reduced to 4.25 mm (**Fig. 3A(iii)**), achieving a 15% reduction in OD. In this regard, we further estimate the relationship between TCD/OD and ID/OD under various TW/OD ratios (denoted as η) to evaluate how the eccentric design can enhance the ID/OD ratio compared to the traditional concentric design with controlled parameters (**Fig. 3B**, see Supplementary Text). The result suggests that the eccentric design can substantially increase the ID/OD ratio. We also compare to other soft continuum robots driven by tendons, external magnetic fields, and fluid (**Fig. 3C**). The comparison reveals that our prototyped single TDCRs excel in room efficiency and robot dimensions among other steerable continuum robots (viz., tendon-driven (*7, 38–42*), magnetically-driven (*15, 43, 44*), pneumatically-driven (*45–47*)), and most concentric push–pull robots (*9–11*). It is worth noting that emerging concentric push–pull robots are constructed from nested thin-walled tubes; consequently, a larger outer diameter, for instance, those in centimeter-size, naturally yields a higher circumferential hollow ratio (*11*). While recent progress in elastic metals and laser machining could further advance their miniaturization (*9*), such approaches are not well-suited to soft-material-based continuum bodies. In a word, our robot designs offer effective solutions to the challenges of continuum robot miniaturization, ensuring the preservation of the working channel and, thanks to the tendon-driven mechanism, maintaining a high force output for interaction.

Figure R17: **Action:** The claim of achieving a high ID/OD ratio is now contextualized to specific cases, e.g., continuum robots made from soft materials and fabricated under identical conditions, with full acknowledgment of the advantages offered by concentric push–pull robots.

Comment 2.2 (Modeling Improvement by introducing Twisting)

The inclusion of twisting induced bending to the robot kinematics is interesting, however it is unclear how much this inclusion improves the modeling results. The presented bending kinematic models are the same as those presented by Webster, Oliver-Butler, and Rothe. It would be good to see the tangible benefits of the authors modeling improvement.

Answer: Thank you for your comments. The phenomenon of twisting-induced bending is observed in 'long' robots, a behavior that has not been fully characterized or exploited in prior works by major teams to this cutting-edge field[2]—such as Profs. Webster, Desai, Rucker, Burgner-Kahrs, Chikhaoui, among others. **Action:** The tangible benefits of our modeling improvement lie in its ability to capture twisting effects and, importantly, to describe the previously neglected twisting-induced bending that occurs in addition to push–pull-induced bending. Please see our revisions below:

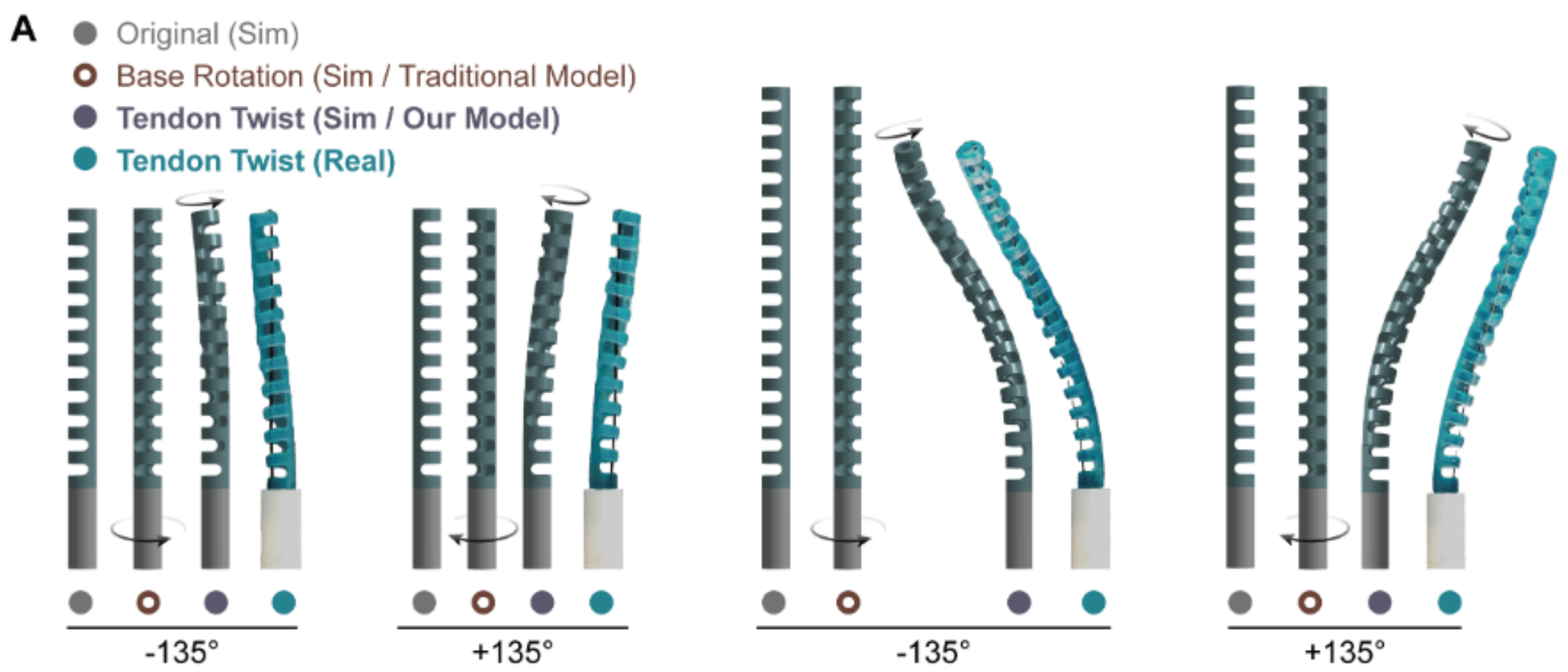

Figure 5: **Comparison of the traditional holistic base/robot rotation model with the proposed twistable tendon model.** **(A)** We capture twisting-borne bending effects by presenting kinematic simulations of two robots (Robot ① and ②) under ±135° base rotation and pure tendon twist, together with the corresponding real-world robot actuated by tendon twist. The traditional approach fails to capture twisting-borne bending, which is non-negligible in long robots, whereas our model can describe the resulting shape and morphology, demonstrating its practical advantages.

To investigate twist-induced bending, **Fig. 5A** compares the traditional holistic base/robot rotation model with our proposed twistable tendon model. Unlike most existing approaches that focus solely on base or robot rotation—which fail to capture the non-negligible bending effect—our model advances the state of the art by incorporating tendon twist, accurately delineating the robot's morphology with notch-level resolution and substantial twist-induced bending. In **Fig. 5B**,

Figure R18: Newly added Figure 5A and in-text description in the manuscript.

[2]Notched-tube continuum robots & Push–Pull tube continuum robots

Comment 2.3 (About the Volumetric Workspace)

The workspace analysis comes across as comparing the areas of projected workspace volumes. The methods and calculation of these values are unclear, and the comparison of projected area does not provide a complete comparison. I believe there are benefits to the omnidirectional motion, however the benefits are not clearly quantified; presenting volumetric workspace metrics or dexterity indices (e.g., Leibrandt (designing robots for reachability and dexterity)) would make the comparison stronger. While potentially outside the scope of this work, it would be an interesting comparison.

Answer: Thank you for the comment. We agree that the description of this section was unclear. Additional details should be provided, including an explanation of how the workspaces and quantitative metrics were obtained.

For the volumetric workspace problem, let's look at the theoretical workspaces, in the form of point clouds, shown in Fig. 6D:

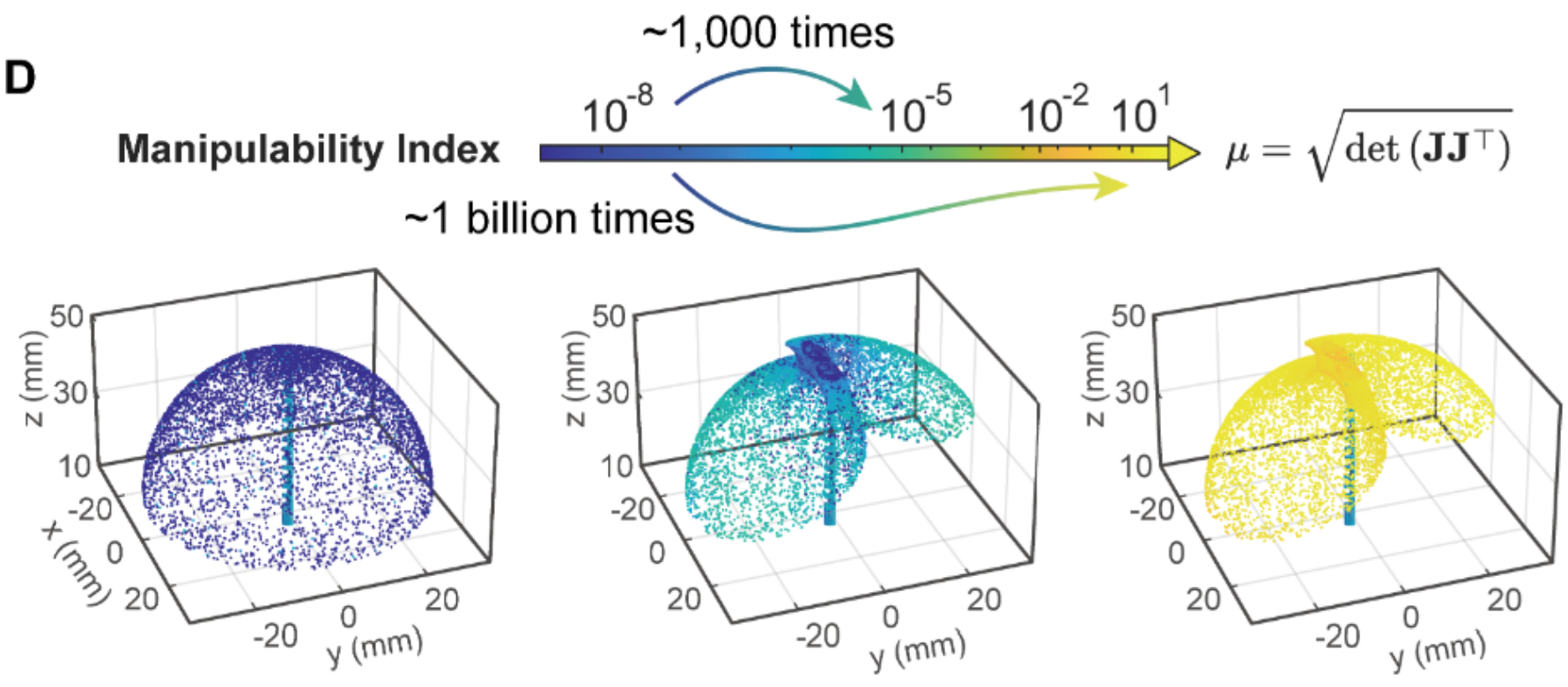

Bend (B)	✓	✓	✓
Twist (T)	✗	✓	✓
Insertion (I)	✗	✗	✓
Rotatable base (R)	✓	✗	✗

- The first point cloud illustrates the workspace and manipulability of the continuum robot tip under pure bending (B) combined with holistic base rotation (R). The second shows bending (B) with twisting (T), and the third includes bending (B), twisting (T), and the intention of insertion (I). The visualized outcome of the insertion is not shown to improve the figure's readability.
- **The distal workspaces generated by bending, twisting, and base rotation are not volumetric but rather form shell-like curved surfaces.** Only when insertion is incorporated does the workspace become volumetric.
- Therefore, we primarily compare their projections on the X–Y and X–Z planes.
- Quantitative results, expressed as percentage changes in area, are presented in the figure and detailed in the text (see Fig. R19).
- Reproducing the new metrics ($\mathcal{D}^{L}_{\mathcal{V}_3}$, $\mathcal{D}^{G}_{\mathcal{V}_3}$, $\mathcal{D}^{G,L}_{\mathcal{V}_3}$) introduced by Leibrandt et al. (2023) would require substantial effort to fully understand and implement, which is beyond the scope of this work. For the present study, we therefore adopt the widely-used manipulability index, $\mu = \sqrt{\det(\mathbf{J}\mathbf{J}^\top)}$, which is the $\mathcal{M}$ in Leibrandt et al. (2023), for ease of interpretation and general understanding. We acknowledge that employing a more advanced metric could provide deeper insights into our robot and actuation design, and

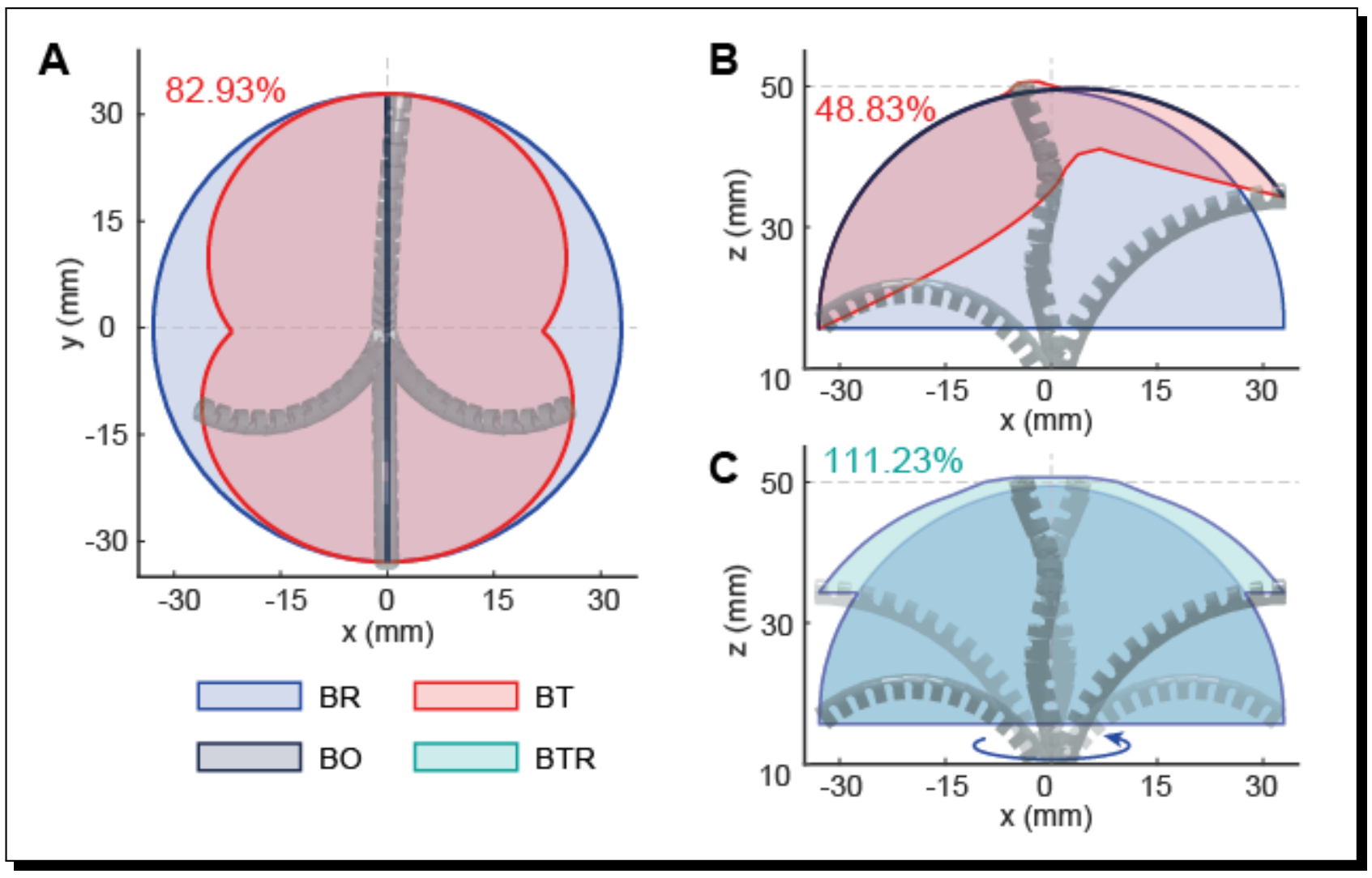

Figure R19: **(A)** Workspace comparison (top view) of Robot ② under various actuation mechanisms, including bending & rotatable base (BR), bending & twisting (BT), bending only (BO), and bending & twisting with a rotatable base (BTR). **(B)** Side view of the workspace comparison among BO, BR, and BT. **(C)** Side view of the workspace comparison between BR and BTR.

Action: We have highlighted this as an important direction in the **Discussion** and future work. See our revision as shown below (Fig. R20):

> **Manipulability breakthrough via tendon twist**
>
> The proposed twisting motion for the tendon joint offers exponentially enhanced manipulability of the continuum robot's tip compared to the traditional bending-rotatable base joint mechanism. As shown in **Fig. 6D**, we compare the manipulability index based on controlled parameters across the robot's motion range and demonstrate a ∼1,000-fold enhancement in the manipulability index by only replacing the rotating base with a twistable tendon motion. Our theoretical derivation (see Supplementary text) demonstrates that an additional twisting motion applied to a single actuation tendon significantly elevates the manipulability of the TDCRs, which remains unreported until now. This PPT joint mechanism has the potential to be generalized to scenarios involving multiple tendons in other TDCRs, enabling the assembly of out-of-plane twisting robot motions introduced by the fixed helical routing method (*71*), but in a controllable manner. Moreover, incorporating additional DOF into a single tendon joint can potentially reduce the necessity of employing multiple continuum segments, which were previously motivated by spatial motion and distal manipulation. While not yet experimentally validated, our twistable tendon actuation approach is expected to markedly improve manipulability, reachability, and dexterity in multi-tendon continuum systems (*72*), enabling more precise operations. Confirming this will require further investigation and should be highlighted as a direction for future work.

Figure R20: **Action:** This additional statement (colored) is made in the **Discussion** part. The reference here is Leibrandt et al. (2023).

- **Action:** In the revised manuscript, we provide a more detailed explanation of why we opt for two-dimensional workspace projections rather than volumetric representations (see Fig. R21). We also introduce a new subsection in the Methods, **Workspace Analysis**, which describes how the workspaces shown in Fig. 6 are computed, including the detailed settings and underlying considerations. See Fig. R22.
- **Action:** Matlab script for reproducing the workspaces is provided on the GitHub repo, as we believe that an interactive, 3D visualization will be more intuitive and easier to interpret than static figures in the manuscript.

Workspace, manipulability, and output force performance

The proposed PPT mechanism offers the robot a distal workspace distinct from the traditional push-pull mechanism on a rotatable robot base. To evaluate the impact on tendon twisting motion and understand how this mechanism compensates for the loss of additional workspace provided by a rotatable robot base, we primarily compare the theoretical distal workspace between bending on a rotatable base (BR) and bending with twisting (BT) on the same Robot ② (**Fig. 6A**). Additionally, we examine a more comprehensive motion involving bending with twisting on a rotatable base (BTR) (**Fig. 6B**). The configuration used for workspace computation is described in the Methods section. Since the distal workspaces generated by robot bending, twisting, and base rotation are not volumetric but rather form shell-like curved surfaces, we primarily compare their two-dimensional projections. We observe that the PPT mechanism results in workspace reduction with the same projection area from the X-Y plane (top view, **Fig. 6A**), covering 82.93% of the BR workspace by area. For Robot ②, the traditional BR motion demonstrates a hemispherical shell-like workspace, while the BT motion induced by the PPT mechanism produces an asymmetric shell. The pulling-induced bending side coincides with the BR robot motion, and the pushing-induced bending side extends toward the X-axis direction (**Fig. 6B**) due to the robot elongation. The twisting motion ($\pm 90°$) can partially cover the BR workspace (considering a symmetrical maximum range on the Y-axis) by 48.83%. Nonetheless, the twisting can exceed $\pm 90°$, and the bending can reach up to over $180°$ on both sides, thereby providing a higher percentage of workspace restoration. Introducing an additional rotating base for the TDCR with a PPT mechanism can enhance the workspace restoration to 111.23%, surpassing the conventional BR mechanism (**Fig. 6C**).

Figure R21: **Action:** The rationale for using area projections/workspaces instead of volumetric workspaces is explained in the revised manuscript.

Workspace Analysis

The theoretical robotic workspaces shown in **Fig. 6A–D** are computed using the kinematic models derived above (including twisting) and the traditional notched-tube kinematics (excluding twisting) (*26*), and are visualized in MATLAB R2022a (academic use, MathWorks). For traditional bending cases (BR, BO), the bending range is constrained to [0°, 180°], and rotation, where applicable, is set to [-180°, 180°]. For the twist-enabled cases (BT, BTR), the maximum positive bending, aligned with the notched side, is set to 180°, while the negative bending is defined as the inverse of the positive joint input, computed via either inverse kinematics or direct forward approximation. Twisting motion is set to [-180°, 180°], with corresponding tendon twist inputs calculated accordingly. This configuration establishes a fair basis for comparing our robot's workspace with that of traditional designs from a joint space perspective. The resulting joint inputs produce a workspace profile that is not perfectly symmetrical, as illustrated in **Fig. 6A–D**. For each case, a total of 100,000 uniformly distributed random joint inputs are sampled.

The 2D projections of the point-cloud–based workspace are generated through orthogonal projection onto the canonical planes (i.e., X-Y and Y-Z planes). To extract the workspace boundary from the scattered point cloud, we first identify the convex hull or alpha-shape boundary of the projected points in each plane. For cases with complex, non-convex workspace geometries (e.g., BT configuration), the boundary is refined by splitting the point cloud into upper and lower regions, computing separate boundaries, and merging them by identifying and excluding intersection regions using polygon intersection operations. The boundary vertices are then sorted sequentially to form a closed polygon. The area of each projected workspace is computed using MATLAB's `polyarea` function, which calculates the area enclosed by the boundary polygon using the shoelace formula. This method sums the cross-products of consecutive vertex coordinates to determine the signed area. Workspace coverage is quantified by computing the percentage ratio between different configurations (e.g., $A_{BT}/A_{BR} \times 100\%$), where A denotes the projected workspace area. The corresponding code for reproducing the results is available.

Figure R22: **Action:** A new subsection, '**Workspace Analysis**,' has been added under '**Kinematics**' in the **METHODS** section. This part details the procedures used to derive the workspaces shown in **Figure 6A–D** and the approach employed for their comparison.

Comment 2.4 (1,000 Times Improvement on Manipulability Index)

Calculation of the manipulability index is clear in the supplementary text, however the rationale behind the claims of 1000 times improvement is unclear to me. It seems intuitive that there will be large improvements to manipulability under this morphology, but the manuscript lacked justification for that claim.

Answer: Thank you for your comments.

- The 1,000 times improvement on the manipulability index is based on the theoretical results.
- **Figure 6D** down below presents the computed manipulability index and illustrates the difference using a gradient color map. MATLAB codes are available for reproduction.

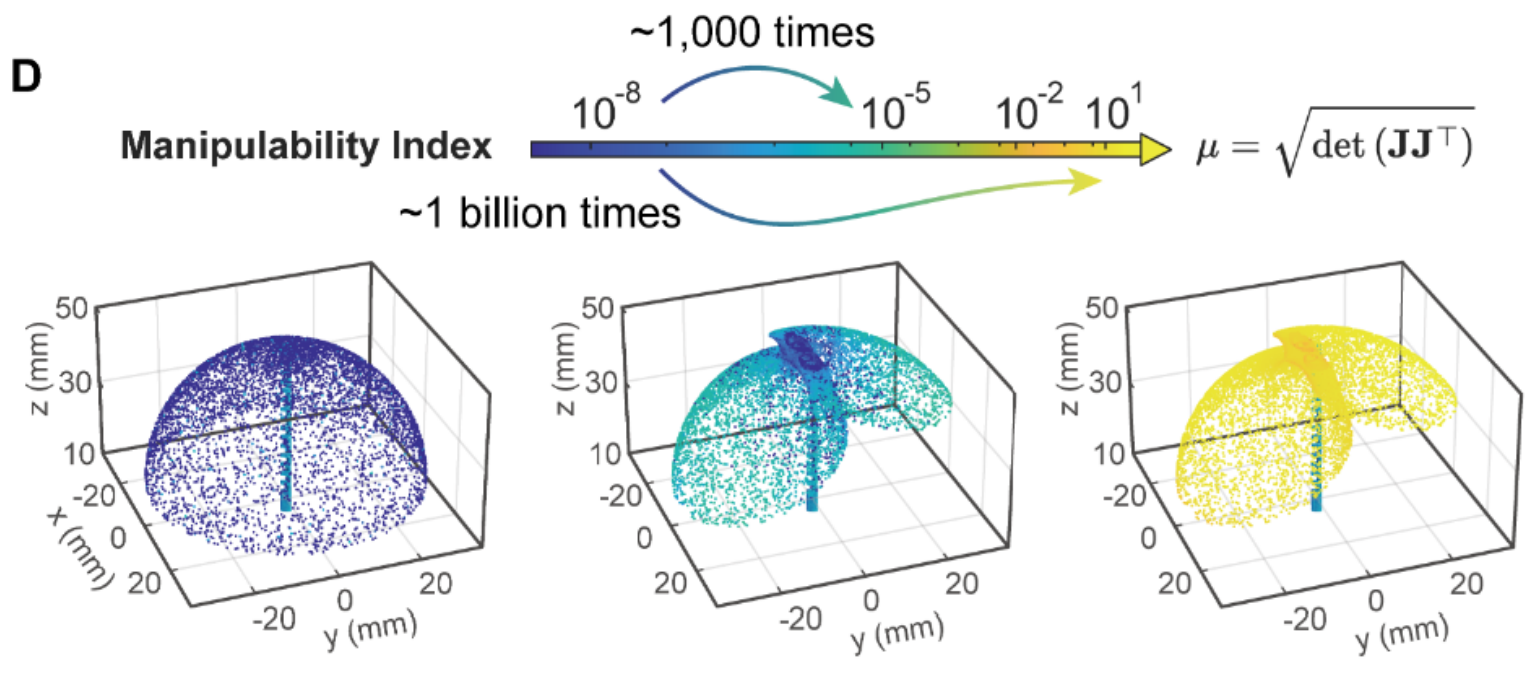

Bend (B)	✓	✓	✓
Twist (T)	✗	✓	✓
Insertion (I)	✗	✗	✓
Rotatable base (R)	✓	✗	✗

- For the 2-DOF systems, the theoretical manipulability index remains around 10^{-8} for BR motion and 10^{-5} for BT motion across the workspace, meaning BT is roughly 1,000 times higher than BR.

Action: In the revised manuscript, we provide a detailed explanation of how the approximate 1,000-fold improvement in the manipulability index was derived. See below (Page 16 in the manuscript):

> The proposed PPT mechanism contributes a substantial enhancement to the robots' manipulability. This is evidenced by calculating the manipulability index (see Supplementary Text). The manipulability index in robotics, introduced by Yoshikawa in 1985 (*49*), is a measure of how easily a robot can move its end effector in different directions from a given configuration. For the 2-DOF systems, the theoretical manipulability index remains around 10^{-8} for BR motion and 10^{-5} for BT motion across the workspace, meaning BT is roughly 1,000 times higher than BR (**Fig. 6D**). It is noteworthy that the manipulability index for BR motion remains nearly uniform across the entire workspace, whereas for BT motion it varies substantially: the index decreases near singular configurations, while other regions exhibit consistently higher manipulability. Moreover, if a general spatial manipulation is desired, an additional prismatic joint for the robot insertion-retraction motion will provide another million-fold manipulability enhancement. Therefore, the improvement in the manipulability offered by the PPT joint can efficiently extend the dexterity of the existing tendon-driven mechanism on a single tendon.

Comment 2.5 (Force Retention Analysis in Non-Tip Bending Plane)

In regards to force application, the ability of such a device to apply a force in the bending and twisting configuration is not clear. The authors state that it retains 70% of the force capabilites in one orientation but forces will be applied in multiple directions as the tension causes bending in a variety of planes depending on twisting. Force application is only tested in the tip bending plane from what I can tell, and more comprehensive results may give a complete understanding of device capabilities.

Answer: Thank you for your comment. We appreciate the suggestion to extend a more comprehensive analysis of the force test. We also believe that readers will be interested in the quantifiable force output performance achieved under our proposed twisting–bending motion.

Action: In our revised manuscript:

- We provide additional experiments on measuring distal lateral force outputs involving robot's twisting motions on bending-perpendicular and non-bending-perpendicular planes (as what you were suggesting). The new results are presented in **Fig. S6** (as shown in Fig. R23 in this letter).
- In the main text, we include a statement directing readers to the Supplementary Materials for further details. (See Fig. R24 and Fig. R25)

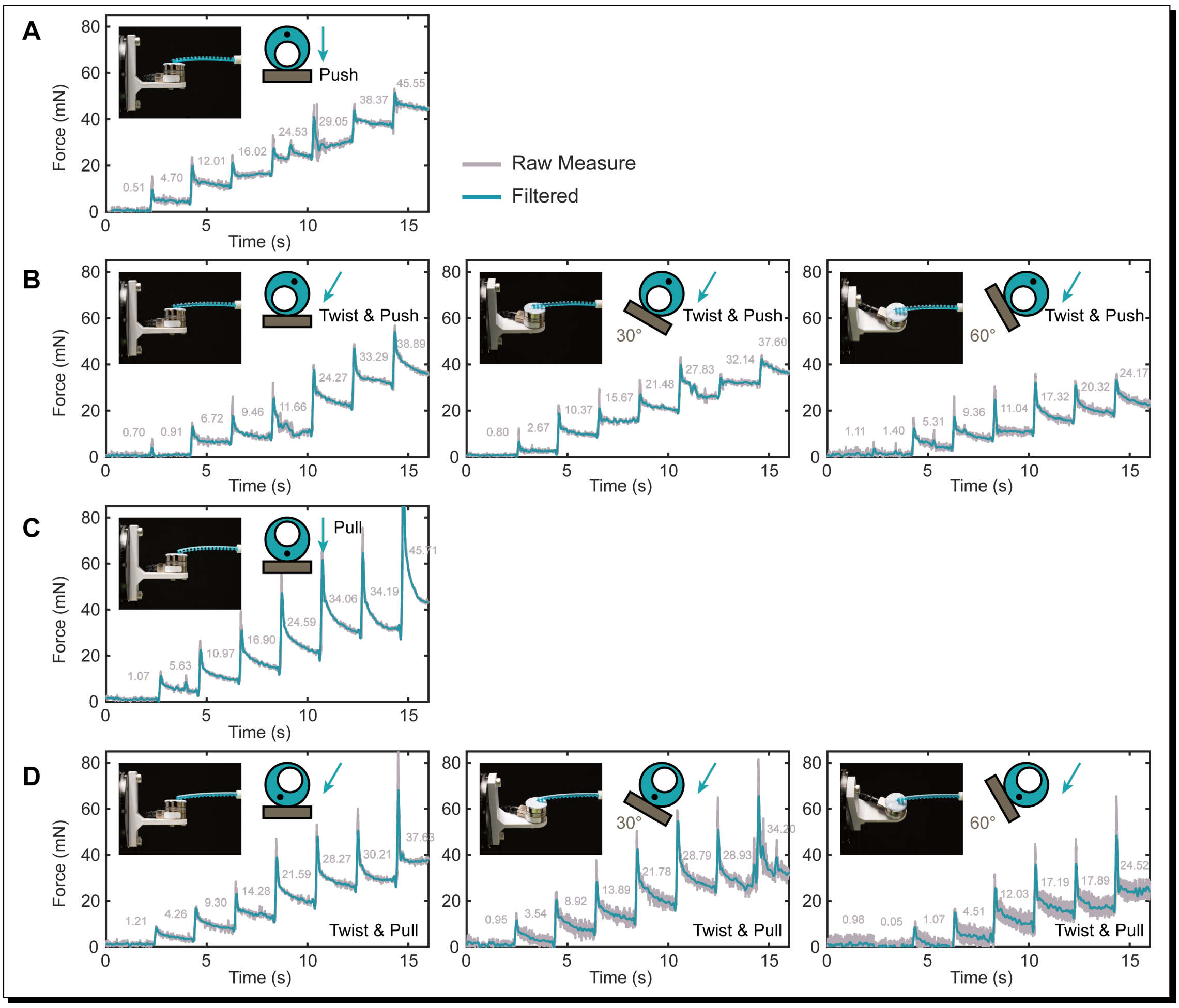

Figure R23: **Action:** These new results are presented in **Fig. S6** in the Supplementary materials. It shows more detailed, ablated results on measuring the force output on the non-bending-perpendicular planes under the same configuration settings (as in **Fig. 6E**).

induced bending motions (**Fig. 6E(iii–iv)**), with a maximum exertion force of around 40 mN. Additional results on measuring the force output on the non-bending-perpendicular planes under the same configuration settings (as in **Fig. 6E**) are supplemented in **Fig. S6**. Consequently, it can be concluded that the twisting-bending configuration retains at least 70% of the exertion force at the distal tip across various directions compared to the default non-twisted bending direction, demonstrating excellent omnidirectional manipulability for the TDCRs.

Hysteresis arising from tendon friction and flexural deformation is generally observed in tendon-

Figure R24: **Action:** In the main text, we include a statement directing readers to the Supplementary Materials for further details.

5. Analysis for Tip Lateral Force Exertion under Different Modes of Motion (Supplementary for Fig. 6E)

The evaluation of tip lateral force under different motion modes, as presented in **Fig. 6E**, was initially restricted to the direction perpendicular to the bending plane. While twisting enables robot–environment interactions across multiple surfaces—one of the key advantages of this motion—we extended the analysis to include forces measured on two additional tilted planes. Using the same experimental configuration as in **Fig. 6E**, the supplementary measurements shown in **Fig. S6** provide an ablation analysis of how exerted forces are retained beyond the bending-perpendicular surface. Specifically, the force sensor was positioned at tilted angles of ±30° relative to the bending-perpendicular plane, as illustrated in **Fig. S6B,D**. The results reaffirm that twisting induces a reduction of 20–30% in output force compared with bending-only scenarios. Nevertheless, tip forces remain effective in interacting with the environment, highlighting the omnidirectional manipulability of TDCRs achieved simply by incorporating an additional twisting motion on the actuation tendon.

Figure R25: **Action:** This is a screenshot of the Supplementary Text to explain the newly added **Fig. S6**.

References

Burgner-Kahrs, J., Rucker, D. C. and Choset, H. (2015), 'Continuum robots for medical applications: A survey', *IEEE Trans. Robot.* **31**(6), 1261–1280.

Childs, J. A. and Rucker, C. (2023), 'A kinetostatic model for concentric push–pull robots', *IEEE Trans. Robot.* **40**, 554–572.

Chitalia, Y., Jeong, S., Yamamoto, K. K., Chern, J. J. and Desai, J. P. (2020), 'Modeling and control of a 2-dof meso-scale continuum robotic tool for pediatric neurosurgery', *IEEE Trans. Robot.* **37**(2), 520–531.

Cho, B. Y., Esser, D. S., Thompson, J., Thach, B., Webster, R. J. and Kuntz, A. (2024), 'Accounting for hysteresis in the forward kinematics of nonlinearly-routed tendon-driven continuum robots via a learned deep decoder network', *IEEE Robot. Autom. Lett.* **9**(11), 9263–9270.

Dupont, P. E., Simaan, N., Choset, H. and Rucker, C. (2022), 'Continuum robots for medical interventions', *Proceedings of the IEEE* **110**(7), 847–870.

Leibrandt, K., Da Cruz, L. and Bergeles, C. (2023), 'Designing robots for reachability and dexterity: Continuum surgical robots as a pretext application', *IEEE Trans. Robot.* **39**(4), 2989–3007.

Oliver-Butler, K., Childs, J. A., Daniel, A. and Rucker, D. C. (2021), 'Concentric push–pull robots: Planar modeling and design', *IEEE Trans. Robot.* **38**(2), 1186–1200.

Rothe, A. K., Brumfiel, T. A., Konda, R., Williams, K. M. and Desai, J. P. (2025), 'Model-based parameter selection for a steerable continuum robot — applications to bronchoalveolar lavage (bal)', *IEEE Robot. Autom. Lett.* **10**(1), 414–420.

Simaan, N., Xu, K., Wei, W., Kapoor, A., Kazanzides, P., Taylor, R. and Flint, P. (2009), 'Design and integration of a telerobotic system for minimally invasive surgery of the throat', *Int. J. Robot. Res.* **28**(9), 1134–1153.

Tummers, M., Boyer, F., Lebastard, V., Offermann, A., Troccaz, J., Rosa, B. and Chikhaoui, M. T. (2025), 'Continuum concentric push–pull robots: A cosserat rod model', *Int. J. Robot. Res.* **44**(2), 216–246.

van Dijk, T., De Wagter, C. and de Croon, G. C. (2024), 'Visual route following for tiny autonomous robots', *Sci. Robot.* **9**(92), eadk0310.

Wang, Y. and Dupont, P. E. (2025), 'Comparison of classical, neural network and hybrid models for hysteretic single-tendon catheter kinematics', *IEEE Robot. Autom. Lett.* **10**(1), 96–103.

York, P. A., Swaney, P. J., Gilbert, H. B. and Webster, R. J. (2015), A wrist for needle-sized surgical robots, *in* 'Proc. 2015 IEEE Int. Conf. Robot. Autom. (ICRA)', pp. 1776–1781.

Zhao, Q., Lai, J., Hu, X. and Chu, H. K. (2022), 'Dual-segment continuum robot with continuous rotational motion along the deformable backbone', *IEEE/ASME Trans. Mechatron.* **27**(6), 4994–5004.

Zheng, X., Jiang, Y., Mete, M., Li, J., Watanabe, I., Yamada, T. and Paik, J. (2025), 'Metamaterial robotics', *Sci. Robot.* **10**(108), eadx1519.

(All of these references have been incorporated into the revised manuscript)

Response Letter – Paper ID: NCOMMS-25-76163-T/A/B

SINGLE TWISTABLE TENDON-DRIVEN CONTINUUM ROBOTS

Jiewen Lai[1,†], Yanjun Liu[1,†], Tian-Ao Ren[2], Yan Ma[1], Tao Zhang[1]
Jeremy Y.C. Teoh[3], Mark R. Cutkosky[2], Hongliang Ren[1]

[1]EE, CUHK [2]ME, Stanford [3]CUHK Medical School

{jwlai;hlren}@ee.cuhk.edu.hk

March 2026

Acknowledgments & Short Summary

Thank you again for reviewing our manuscript. Based on your latest comments, we have addressed each point with necessary clarifications, amendments, and revisions. To maintain a sober writing style, we have revised the manuscript by refraining from using terms implying novelty or exaggerated language. We would like to again invite you to use this response letter as a guide sheet to examine our revised manuscript (NCOMMS-25-76163-T/A) attached.

Color Scheme for this response letter
Red - Reviewers' comment
Blue - Our response & clarification
Green - Our action in the revised manuscript
Brown - Hyperlink

Color Scheme for FIRST ROUND of revision
Blue – Revision based on Comments from Reviewer#1
Pink – Revision based on Comments from Reviewer#2

Color Scheme for SECOND ROUND of revision
Cyan – Revision based on Comments from Reviewer#1
Orange – Revision based on Comments from Reviewer#2

Contents

Reviewer#1 **2**
Comment 1.1 (Precision in describing Hysteresis) 2
Comment 1.2 (Precision in describing Manipulability Enhancement) 3
Comment 1.3 (Typographical and Formatting Errors) 6

Reviewer#2 **7**
Comment 2.1 (Modify Figure 1 and Figure 5) 7
Comment 2.2 (Workspace Comparison) 9
Comment 2.3 (Clarify on Figure 6C caption) 12
Comment 2.4 (Avoid Exaggerated Comparison in Manipulability) 13

Reviewer#1 (See CYAN text in the revised manuscript)

The authors have done an impressive work on addressing my previous concerns. The addition of the mock intravascular experiment (Figure 5) perfectly justifies the motivation for a twistable tendon over a rotating base. Furthermore, the elaboration on the mechanical principles (center of stiffness offset), operational boundaries, and material failure modes significantly strengthens the scientific rigor of this manuscript. Before final acceptance, I only have a few minor suggestions for the authors to polish the manuscript:

Comment 1.1 (Precision in describing Hysteresis)

Precision in describing hysteresis (Page 19, Line 356): The phrase "tendon-twisting is the primary cause of robot motion hysteresis" is slightly imprecise. Tendon-twisting is the action, whereas the underlying physical causes are likely the tendon-routing friction (amplified during twisting) and the viscoelasticity/residual stress of the soft material. Consider rephrasing to reflect the physical root causes more accurately.

Answer: Thank you for your comment. This is very helpful. We agree that "tendon-twisting" describes an action rather than the underlying physical mechanisms.

exhibits rapid deformation upon tendon release and fails to return to the initial pose. A similar result is observed in the pushing-induced bending case (**Fig. 6F(ii)**). In an active tendon-twisting case (**Fig. 6F(iii)**), the forward and inverse twisting (from 0° to 175°, and from 175° to 0° in the joint space) show a more symmetrical plotting profile than the twist-release convention, suggesting a highly maneuverable angular velocity in robot twisting. However, reverse tendon twisting, even when ending at the zeroing position, results in over-twisting tip motion in the opposite direction with substantial hysteresis, whereas the twist-release method results in insufficient reverse motion (i.e., under-twisting), failing to return to the initial robot pose. The over-twisting occurs due to residual stress in the low-elastic soft material, which is subjected to high angular acceleration of the tendon. A similar result can be observed in the case of bending-twisting coupled motion (**Fig. 6F(iv)**). The observed motion hysteresis is primarily caused by amplified tendon-routing friction during twisting, together with the viscoelasticity and residual stress of the soft material. Nevertheless, hysteresis can be easily addressed using closed-loop control methods (*11, 50, 51*), which are beyond the scope of this work. **Figure 6G** presents the corresponding repeated trials (three times) for the results shown in **Fig. 6F**, reaffirming the robot motion hysteresis, particularly when tendon twisting is involved. Additionally, it is observed that tendon pushing can elongate the robot's length under excessive bending (**Fig. 6G(ii)**), resulting in a plateau in the bending angle. This plateau can be overcome by further stretching the tendon. The difference in bi-directional bending aligns with our

19

Figure R1: **Action:** We have revised the manuscript to attribute the observed hysteresis to increased tendon-routing friction during twisting and to the viscoelasticity and residual stress of the soft material, and we added a short explanation. The revised sentence appears on Page 19, near Line 356, as shown above in cyan text.

Comment 1.2 (Precision in describing Manipulability Enhancement)

Precision in describing manipulability enhancement (Page 34, Line 635): The claim that the proposed mechanism "enhances the robot's manipulability by over 1,000 times" is mathematically accurate based on the provided Yoshikawa index comparison (10e-5 vs. 10e-8). However, presenting this as a strict 1,000-fold improvement may be perceived as slightly exaggerated, as the index drops exponentially when approaching near-singular configurations. Consider softening or contextualizing this claim to reflect the underlying mechanism more rigorously.

Answer: Thank you for your comment. We agree that stating the improvement as an absolute, configuration-independent factor can be misleading, because the Yoshikawa manipulability index μ falls off rapidly near singularities. This is evidenced by our **Figure 6D** (uneven manipulability distribution).

Action: Following Reviewer#2's suggestion (which we find makes sense), we remove the BTI workspace, the one with pushing, to maintain a 2 DOFs vs. 2 DOFs fair comparison. See our revised **Figure 6D** below: with a in-figure note saying the μ drop near singularities.

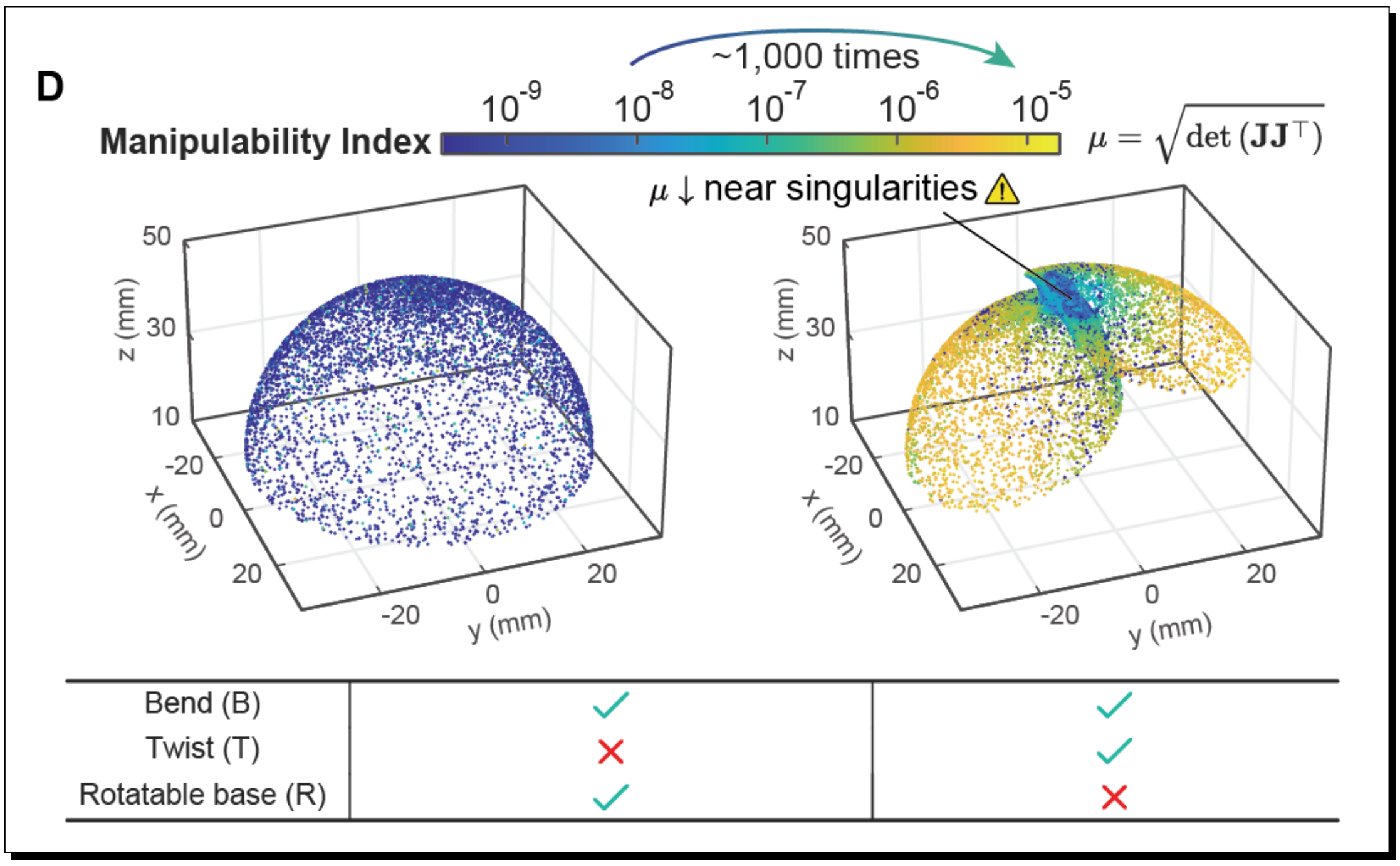

Figure R2: **Action:** We have updated Figure 6D to reminder readers that μ is configuration-dependent, and it drops near singularities.

Figure 6: **Motion and force performance of the TDCR (Robot ②) under the single tendon PPT mechanism.** **(A)** Workspace comparison (top view) of the same robot under various actuation mechanisms, including bending & rotatable base (BR), bending & twisting (BT), bending only (BO), and bending & twisting with a rotatable base (BTR). **(B)** Side view of the workspace comparison among BO, BR, and BT. Note that tendon pulling and pushing produce distinct bending angles under equal actuation. **(C)** Side view of the workspace comparison between BR and BTR. **(D)** The manipulability of the robot tip among BR and BT indicates that the tendon bending-twisting mechanism improves over 1,000 times in most configurations compared to the traditional bending-rotating base mechanism. Note that the manipulability is configuration-dependent. **(E)** Tip lateral force exertion under different modes of motion, viz., bending (pushing/pulling) and bending with twisting (pushing/pulling & twisting). **(F)** Hysteresis tests for different modes of motion, including (i-ii) bending, (iii) twisting, and (iv) coupled motion. We compare the hysteresis effect between the opposite joint motion (ours) and the actuate-release joint motion (a commonly adopted mechanism for other TDCRs), evidencing significant hysteresis in joint twisting in both cases. **(G)** Hysteresis tests with repeated trials for different modes of motion illustrating the joint-tip motion relationship.

Figure R3: **Action:** Updated caption for Figure 6D.

Action: In the revised manuscript, we have softened the wording to state the improvement as an approximate, **configuration-dependent** change. The revisions are shown in (see figures below):

- Discussion section (the place you mentioned), see Figure R4 for revision
- Results section (the place where we explain the **Figure 6D**), see Figure R5 for revision

We really appreciate the suggestion and have updated the text accordingly.

Manipulability breakthrough via tendon twist

The proposed twisting motion for the tendon joint substantially increases the manipulability of the continuum robot's tip compared to the traditional bending-rotatable base mechanism. Our theoretical derivation shows that adding a twisting degree of freedom to a single actuation tendon can elevate manipulability, a possibility that, to our knowledge, has not been extensively explored. At the tested configurations, replacing the rotating base with a twistable tendon motion increases manipulability by roughly three orders of magnitude in the poses we examined (**Fig. 6D**). Note that this improvement is configuration dependent: the manipulability index can fall sharply as configurations approach singularities, so the multiplicative factor reported here applies to major poses and nearby configurations rather than uniformly across the entire workspace. This PPT joint mechanism has the potential to be generalized to scenarios involving multiple tendons in other TDCRs, enabling the assembly of out-of-plane twisting robot motions introduced by the fixed

Figure R4: **Action:** Revised wordings in the discussion section.

The proposed PPT mechanism contributes a substantial enhancement to the robots' manipulability. This is evidenced by calculating the manipulability index (see Supplementary Text). The manipulability index in robotics, introduced by Yoshikawa in 1985 (*49*), is a measure of how easily a robot can move its end effector in different directions from a given configuration. For the 2-DOF systems, the theoretical manipulability index remains around 10^{-8} for BR motion and 10^{-5} for BT motion across the workspace, meaning BT is roughly 1,000 times higher than BR (**Fig. 6D**). It is noteworthy that the manipulability index for BR motion remains nearly uniform across the entire workspace, whereas for BT motion it varies substantially: the index decreases near singular configurations, while other regions exhibit consistently higher manipulability. Moreover, if a general spatial manipulation is desired, an additional prismatic joint for the robot insertion-retraction motion will provide another million-fold manipulability enhancement. Therefore, the improvement in the manipulability offered by the PPT joint can efficiently extend the dexterity of the existing tendon-driven mechanism on a single tendon.

Figure R5: **Action:** Revised wordings in the results section.

Comment 1.3 (Typographical and Formatting Errors)

Typographical and Formatting Errors: While the manuscript has been significantly improved, there are still a few minor typographical and spelling errors that should be corrected prior to publication:

- In the manuscript (Figure 11E, Page 32): In the second sub-figure of the top row, the text label is misspelled as "Appraoch". It should be corrected to "Approach"
- Typographical error in section heading (Page 50, Line 963): The section header "Robot fabrication and mechantronics" contains a typo. It should be corrected to "Robot fabrication and mechatronics"?

Answer: The typos are now corrected. Thank you for your careful reading and for pointing out the small mistakes we missed.

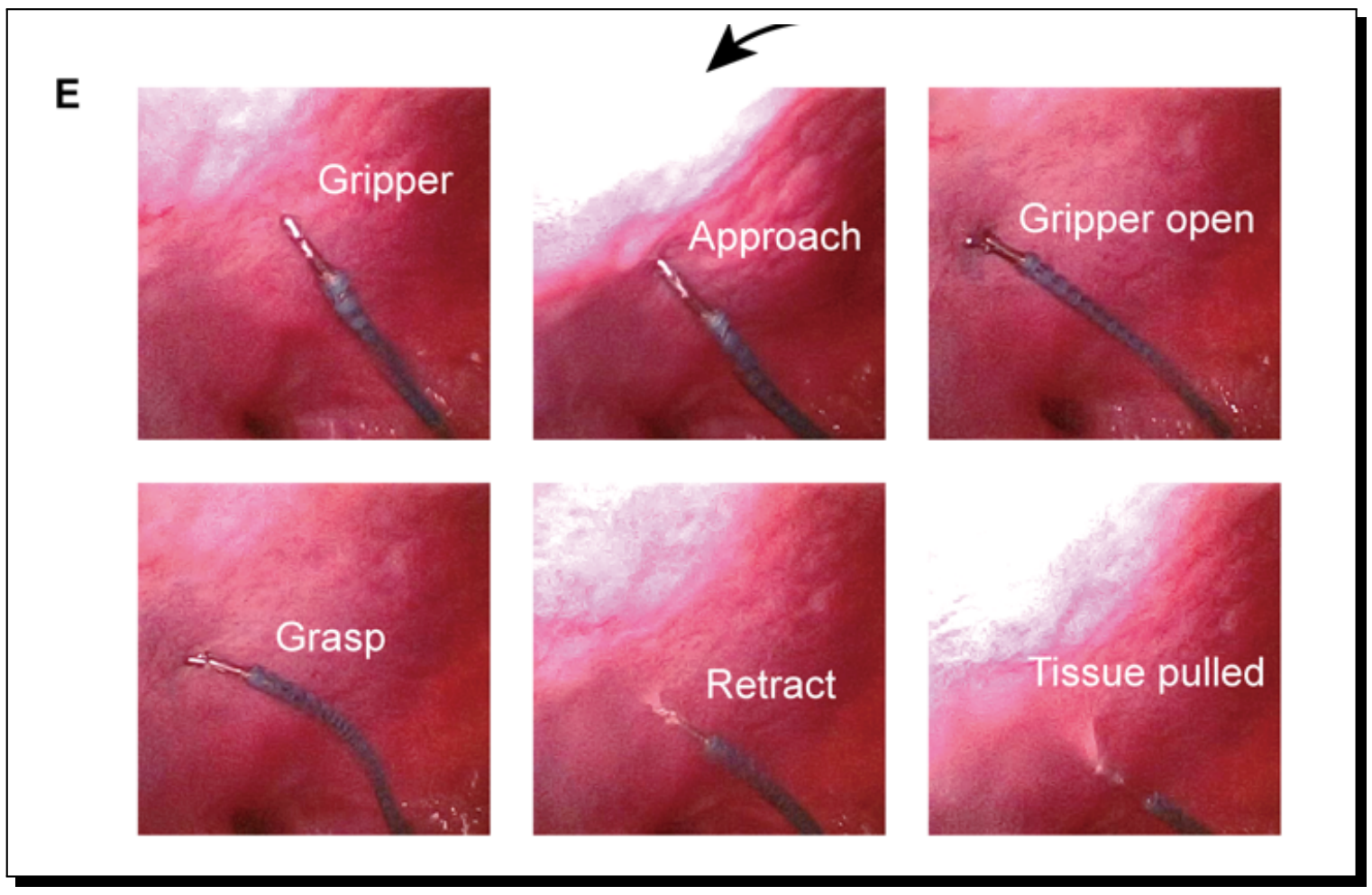

Figure R6: **Action:** Revised typo: **Figure 11E** "Appraoch" → "Approach"

Robot fabrication and mechatronics

3D-printed TDCRs

The continuum body is made from a digital blend of VeroCle
like polymer using a PolyJet 3D printer (J826 Prime, Strat:

Figure R7: **Action:** Revised typo: Section header "mechantronics" → "mechatronics"

Reviewer#2 (See ORANGE text in the revised manuscript)

Comment 2.1 (Modify Figure 1 and Figure 5)

Figure 1 does not properly convey the complex shapes that should be produced by the PPT under twist and pull or twist and push. The shapes look comparable to A despite the notches rotating. Potentially including another view would communicate the 3D effects of the actuation.

The addition and emphasis of direct control over distal tip orientation compared to base first bending in friction-heavy environments is compelling and a good addition to the contributions.

In Fig. 5, please label the robots in the figure as well

Answer: Thank you for your suggestion.

Action: We have updated **Figure 1** and **Figure 5** accordingly. See updated figures below:

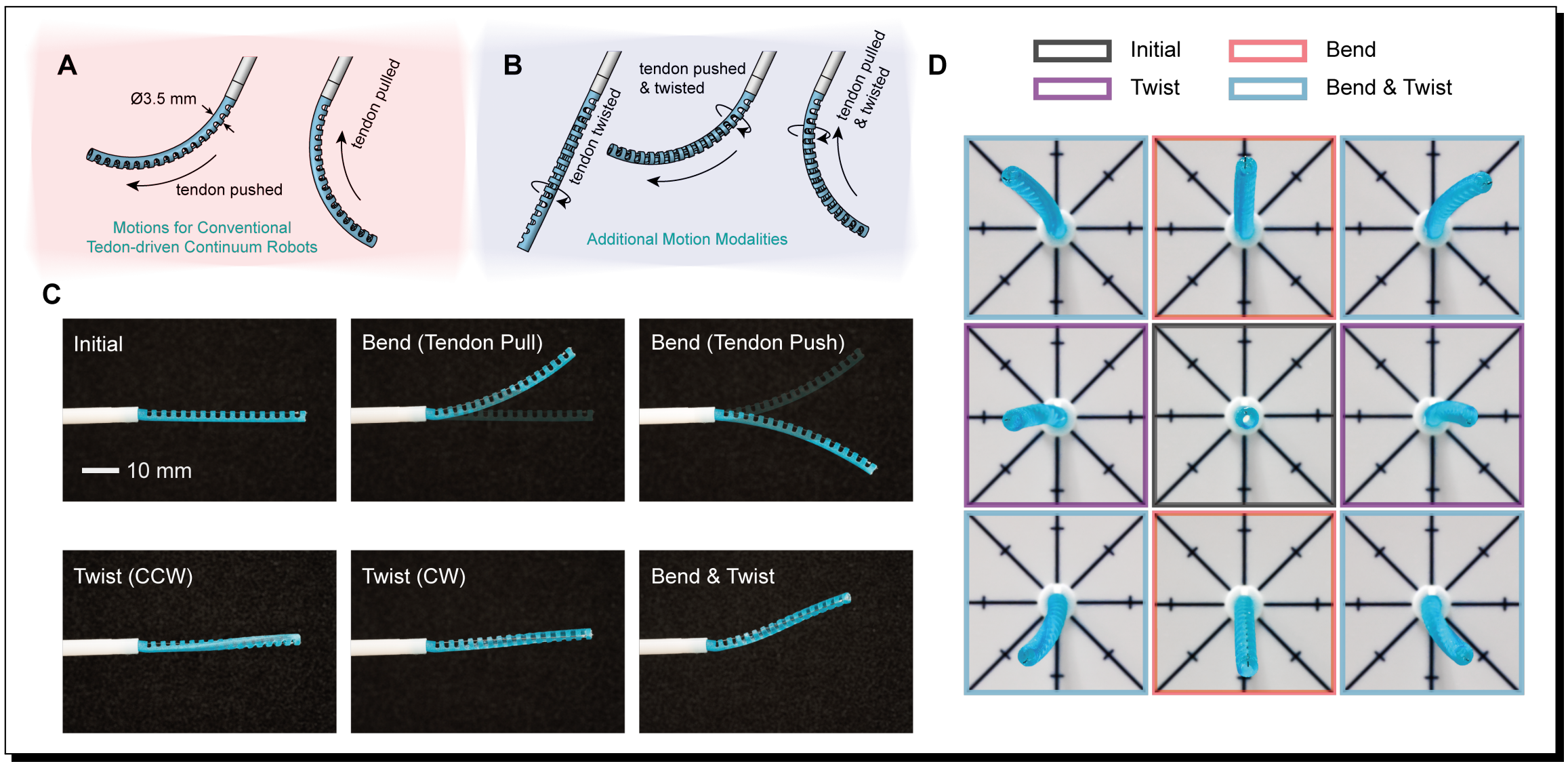

Figure R8: **Action:** Updated Figure 1 with Panel D, showing the front view.

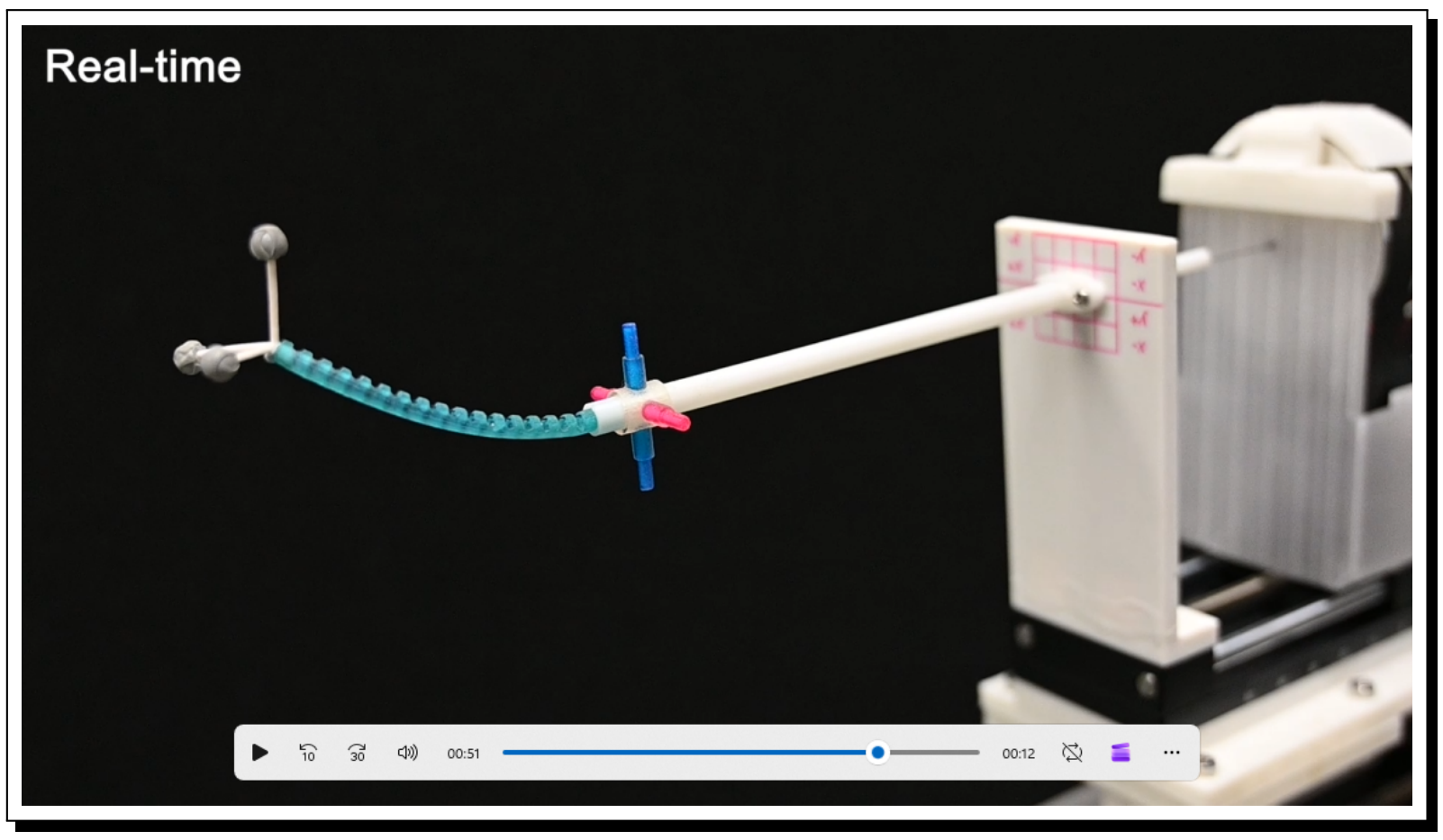

Figure R9: **Action:** Although not directly visible in the main text, our **Movie S2** provides a clear illustration (from an oblique observation angle) of the complex shape.

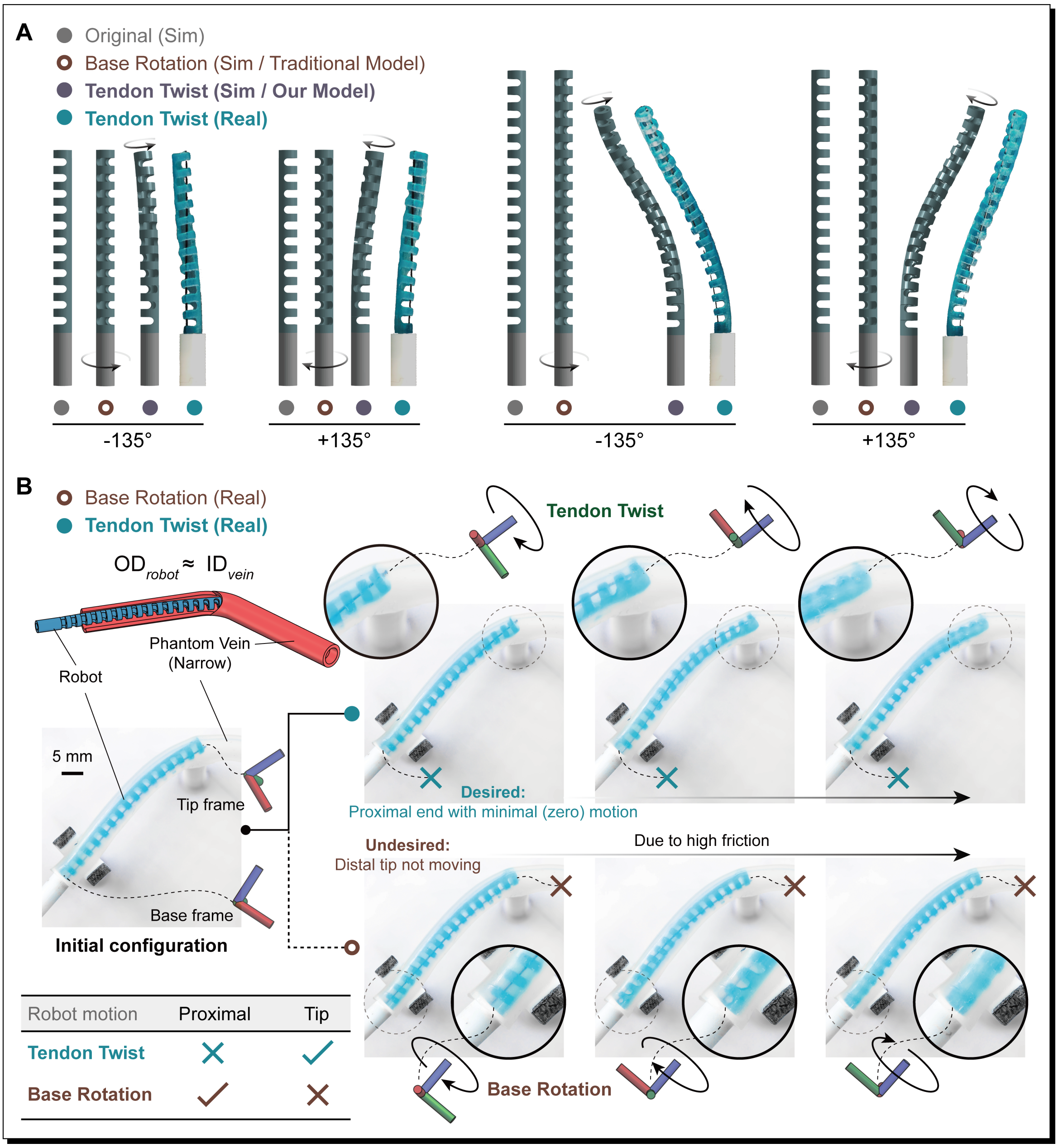

Figure R10: **Action:** In Figure 5, we added a label legend to Panel B. This should make the comparisons clearer.

In addition, although not directly visible in the main text, our **Movie S2** provides a clear illustration (from an oblique observation angle) of the complex shape.

Comment 2.2 (Workspace Comparison)

Thank you for the additional details on the computation of the workspace. The comparison of projected workspaces is still not fully convincing to me. Though the workspace shapes are shell-like structures, the shells take on a surface in the 3D space, and the areas of such surface can be compared, rather than the projection. The projection into 2D of the 3D still does not allow for completely accurate comparison of the workspaces (i.e. additional workspace at the edge of the curve would not correspond to a proportional increase in the workspace area once projected).

Answer: Thanks for your comment. We agree that orthogonal projection of such shell-type structures onto a side plane leads to substantial spatial overlap due to self-occlusion or curvature. Any area metric derived from these projections lacks actual physical meaning and cannot be representative for workspace.

Action: We have retained the projected views in the revised **Figure 6A–C** solely for qualitative illustration of overall workspace shape without reporting any numerical area values derived from them—**thus, only for visualization purpose**. In the revised **Figure 6A**, we estimated the curved surface areas of BR and BT, and found that the area of BT is approximately 64.87% of that of BR. These surface areas were obtained via point-cloud meshing using alpha-shape-based triangulation in MATLAB (Fig. R12). Codes are available in our GitHub repo as part of the supplementary materials.

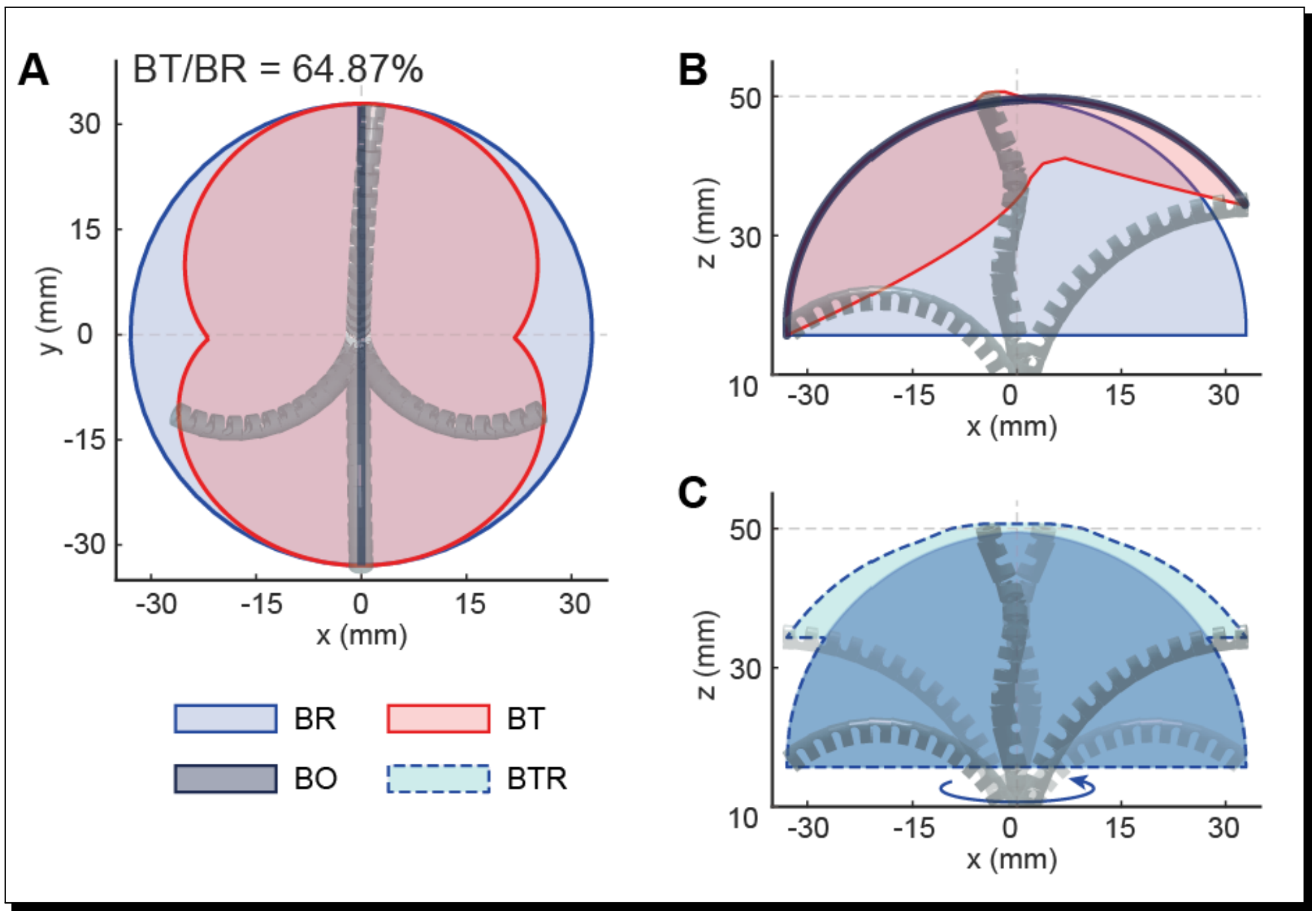

Figure R11: **Action:** Projections are only for visualization purposes. We estimated the curved surface areas of BR and BT, and found that the area of BT is about 64.87% of that of BR. The areas are no longer based on their projections but on their actual curved surface areas.

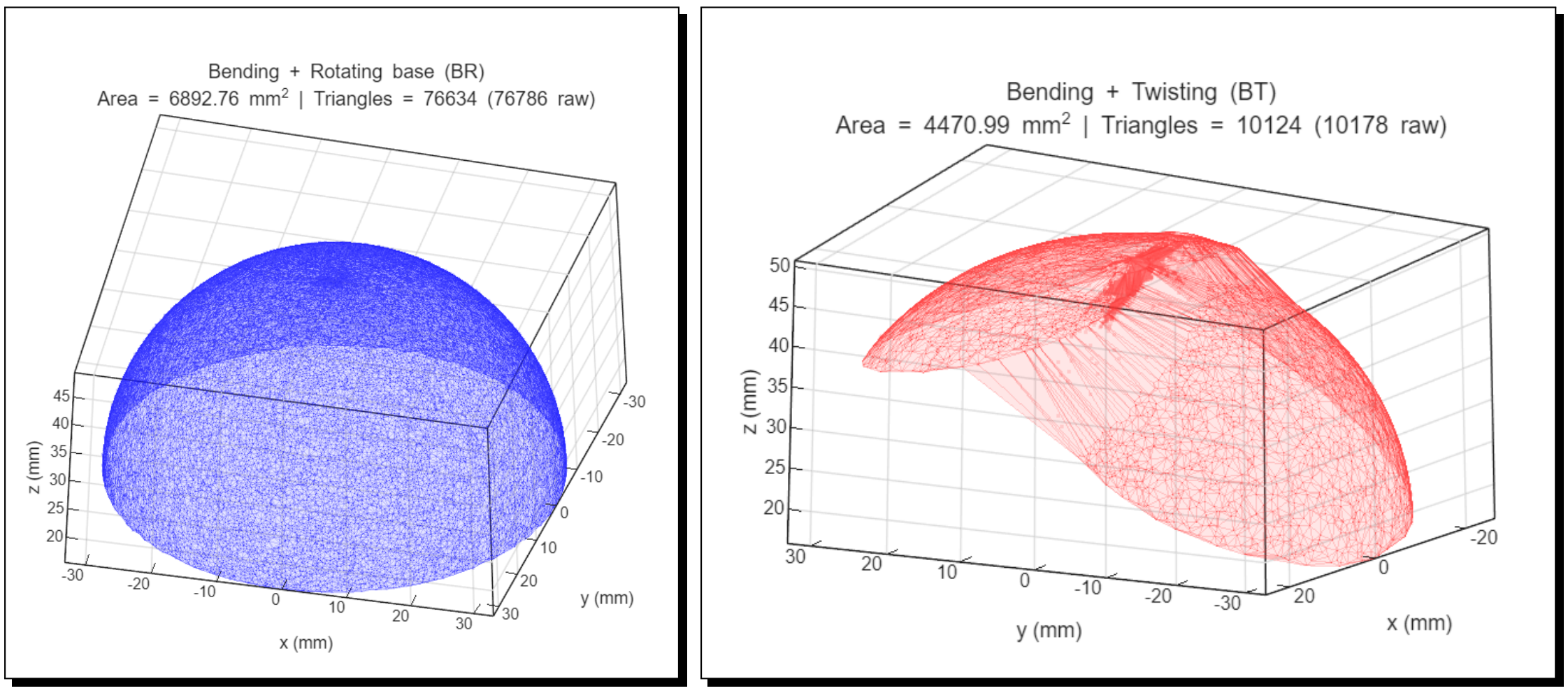

Figure R12: **Action:** We utilize a series of MATLAB functions to compute the surface area from the workspace point cloud. The method is described in the Method: Workspace Analysis section, and the corresponding code is available on our GitHub repository. These two raw figures are not included in the manuscript.

The configuration used for workspace computation is described in the Methods section. Since the distal workspaces are not volumetric but rather form shell-like curved surfaces, we primarily visualize them through two-dimensional projections (**Fig. 6A–C**). For Robot ②, the traditional BR motion demonstrates a hemispherical shell-like workspace, while the BT motion induced by the PPT mechanism produces an asymmetric shell. We estimate the curved surface areas of BR and BT using triangulated meshes (with code provided in the supplementary), and find that the area of BT is approximately 64.87% of that of BR. The pulling-induced bending side coincides with the BR robot motion, and the pushing-induced bending side extends toward the X-axis direction (**Fig. 6B**) due to the robot elongation. It should be noted that, in our analysis, tendon pulling and pushing produce unequal bending angles under identical actuation levels. Nonetheless, the twisting can exceed ±90°, and the bending can reach up to over 180° on both sides, thereby providing a higher

16

Figure R13: **Action:** The colored sentences are our revisions that address your suggestion.

The 2D projections of the point-cloud–based workspace are generated through orthogonal projection onto the canonical planes (i.e., X-Y and Y-Z planes). To extract the workspace boundary from the scattered point cloud, we first identify the convex hull or alpha-shape boundary of the projected points in each plane. For cases with complex, non-convex workspace geometries (e.g., BT configuration), the boundary is refined by splitting the point cloud into upper and lower regions, computing separate boundaries, and merging them by identifying and excluding intersection regions using polygon intersection operations. The boundary vertices are then sorted sequentially to form a closed polygon.

The surface area of the shell-like 3D workspace was quantified directly from the scattered point cloud using an alpha-shape-based surface reconstruction workflow implemented in MATLAB. The characteristic radius for triangulation was estimated from the point cloud using `criticalAlpha` function. An `alphaShape` object was then constructed and its boundary facets extracted via `boundaryFacets` to yield the triangulated open shell. Spurious bridge-like triangles arising from sparse sampling were suppressed by rejecting any triangle whose longest edge exceeded a threshold. The final surface area was obtained by summation of retained triangle areas. Workspace coverage is quantified by calculating the percentage ratio between different actuation modes.

Figure R14: **Action:** In our updated METHOD section, we describe how the curved workspaces are computed and compared. The upper pink text reflects our previous revision, where we clarified how the workspace projections are visualized. The newly added paragraph below, in orange, is for this round of revision and explains how the surface areas are computed, including the key MATLAB functions used.

Comment 2.3 (Clarify on Figure 6C caption)

Figure 6C, Why is the BTR not able to bend to the same amount as the BR? Clarify this in the caption.

Answer: Thank you for your careful checking. In our analysis, tendon pulling and pushing result in unequal bending angles under **identical actuation levels**. This was also explained in the method section. For instance, an actuation that produces a 180-degree bend on the notched side cannot achieve the same bending on the opposite side. By leveraging bending on both the notched and unnotched sides equally, the robot's workspace can be further expanded. In our work, we present a more conservative comparison to avoid exaggeration. This is also reflected in **Figure 6B & D**.

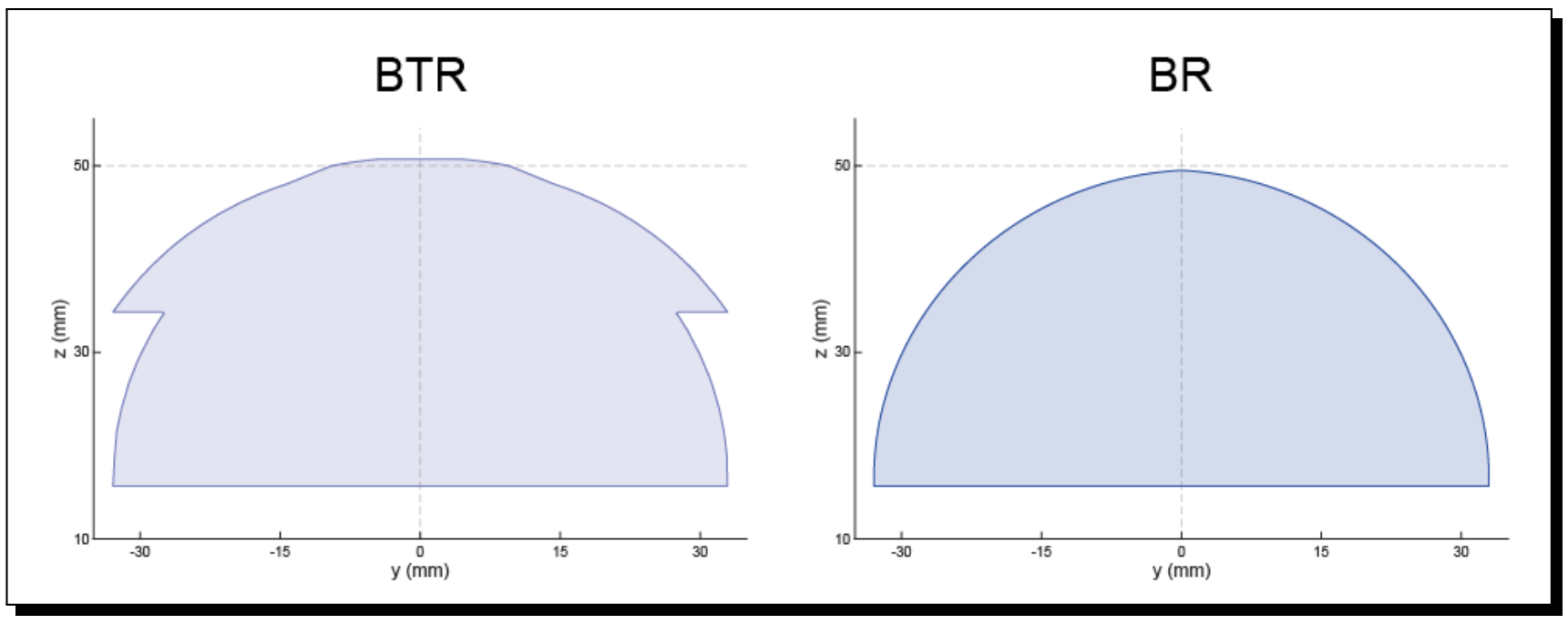

Figure R15: In our analysis, tendon pulling and pushing produce unequal bending angles under identical actuation levels.

Action: We realize that our choice of lines made the overlaid workspace projection unclear to interpret. We revised the line in **Figure 6C** to a dashed format for clearer presentation. In addition, we added explanatory sentences (both in the main text and in the caption) to highlight the unequal bending observed in cases involving twisting (T).

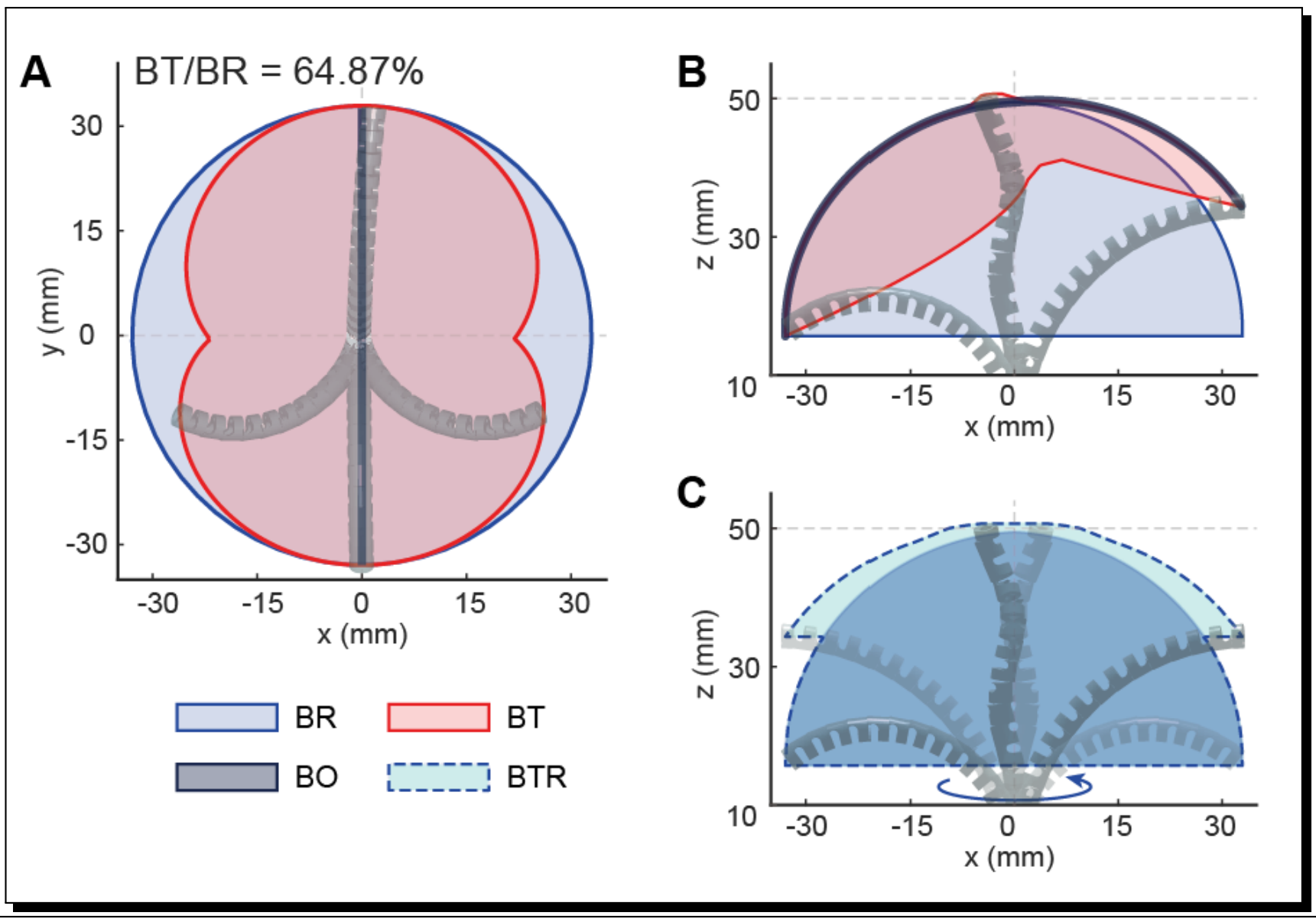

(B) Side view of the workspace comparison among BO, BR, and BT. Note that tendon pulling and pushing produce distinct bending angles under equal actuation. **(C)** Side view of the workspace comparison between BR and BTR. **(D)**

Figure R16: **Action:** **Figure 6C** and BTR plot have been updated with **dashed lines** to highlight their differences more clearly. We have also increased the line thickness of the BO curve. Orange sentence is our added clarification for **Figure 6** caption.

Comment 2.4 (Avoid Exaggerated Comparison in Manipulability)

Thank you for the specification on the manipulability. The comparison between 2 DoF and 3 DoF systems leads to an exaggeration of the device benefits regarding manipulability. A comparison with a base rotation, bending, and insertion robot would contextualize the ∼1 billion times increase. Alternatively, the comparison to a 3 DoF system could be removed entirely, with a comparison between the 2 DoF systems being the most relevant to this work.

Thank you for the additional experiments and commentary on the loading under bending and twisting.

Answer: Thank you for your suggestion. We agree that the most relevant and meaningful comparison lies between the two 2-DOF actuation strategies, namely the BT and BR modes. One of the key features we want to highlight in our work is that incorporating twisting (as opposed to the traditional base rotation) yields substantial improvements in manipulability under the same bending and overall DOF constraints.

Action: Thus, according to your suggestion, we have

- Removed the comparison involving the 3-DOF BTI mode (I for inserting);
- Removed any associated claims of a 1-billion-fold increase in manipulability.

The revised **Figure 6D** and accompanying text now focus exclusively on the comparison between BR and BT.

As we have removed the BTI, we recomputed the manipulability index and updated the color bar scale accordingly. The result is now even better in terms of visualization, showing distinct changes in colors from BR to BT.

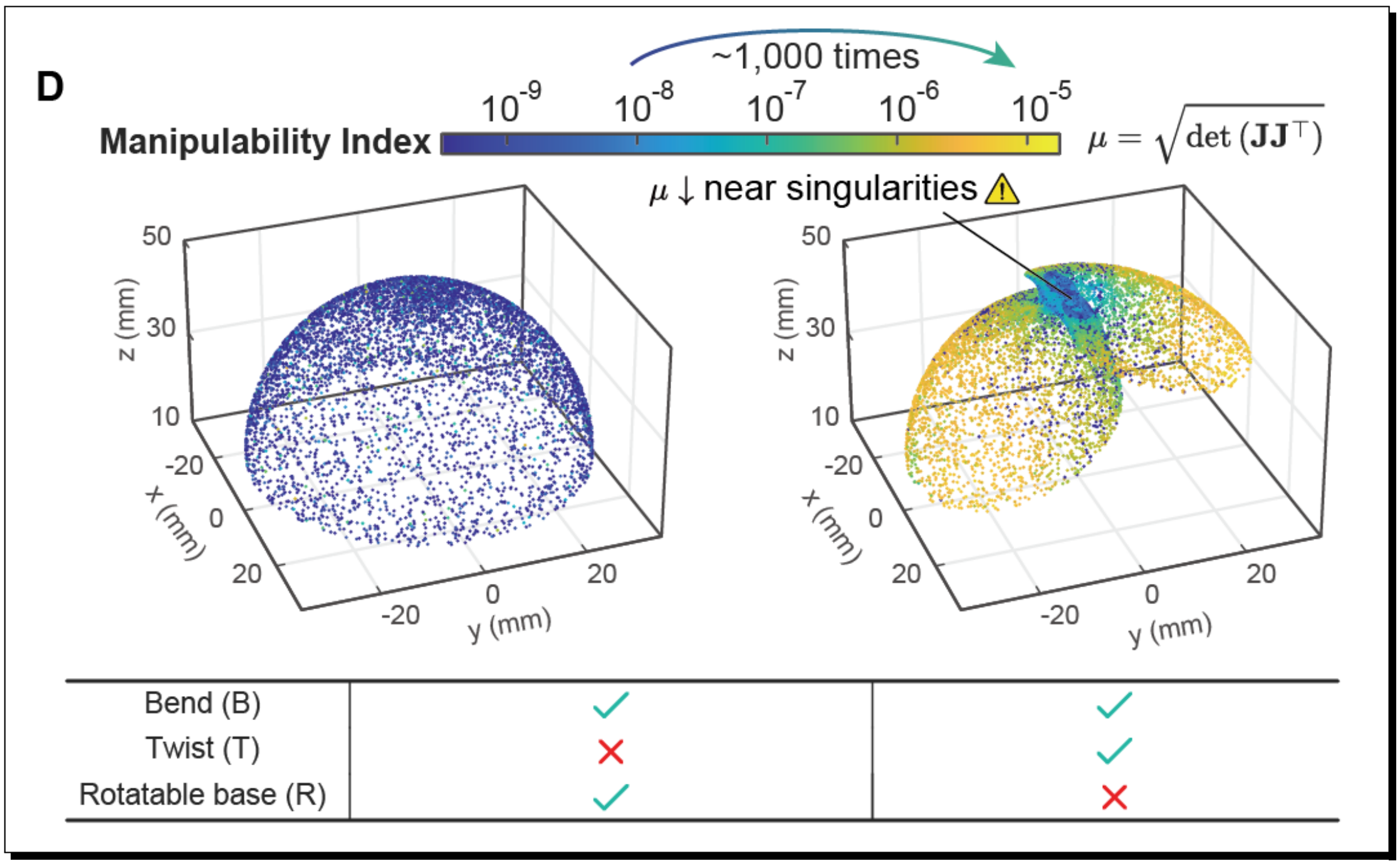

Figure R17: **Action:** Revised Figure 6D — and no more "1 billion" claim in the entire manuscript. As we have removed the BTI, we recomputed the manipulability index and updated the color bar scale accordingly.

Sober Writing Check

Abstract

- "... we introduce a new class of continuum robots capable of ..." (Keep)
- "This advancement broadens conventional thinking about how tendon-driven continuum robots can be driven and ~~paves a new way~~ for designing continuum robots." →

  "This advancement broadens conventional thinking about how tendon-driven continuum robots can be actuated and informs new design strategies for such systems." (Weaker tones)
- " We derive, for the first time, the kinematics for this class of continuum robots, establishing a theoretical foundation for their control and analysis." (Keep)

Introduction

- "Flexible robots can exhibit diverse motion modalities (including twisting) and ~~extreme~~ miniaturization (600 um in outer diameter) when they take on other forms, such as origami structures (15), pre-programmed pneumatic structures (16), and magnetically-driven structures (17), among others. Nonetheless, these novel structures and actuation methods may trade off spatial manipulability ..."→

  "Flexible robots can achieve diverse motions, including twisting, and substantial miniaturization (e.g., 600 um outer diameter (15)) when implemented as origami structures (16), pre-programmed pneumatic devices (17), and magnetically driven systems (18), and related architectures. Nonetheless, these novel structures and actuation methods may trade off spatial manipulability ..." (Introducing others' work. I think it is fine to recognize these works as novel)
- To the best of our knowledge, this is the first general kinematic model proposed for single-tendon-driven continuum robots incorporating PPT tendon joints. (Keep)

Results

- "Despite its ~~ultra~~thin diameter of 3.5 mm ..."
- "~~Successful~~ navigation and inspection within both phantom models and *ex vivo* porcine organs underscore the versatility and clinical potential of our proposed robots and the ~~novel~~ actuation mechanism. By offering high precision and enhanced flexibility, this ~~ultra~~thin, fully steerable robotic endoscope contributes to the advancement of robotic endoluminal navigation technologies." →

  "The navigation and inspection results in phantom models and *ex vivo* porcine organs suggest our robots and actuation approach is versatile and potentially clinically useful. Its precise steering and flexible operation can assist ongoing improvements in robotic endoluminal navigation." (More neutral)
- "... demonstrating ~~excellent~~ effective omnidirectional manipulability for the TDCRs. " (More neutral)

Discussion

- "The ~~novel~~ concept of coupling a prismatic (push-pull) and rotational (twist) joint at the same anchoring point on a single tendon, which goes beyond the separated radial and oblique muscle forms in biological systems (23,24,31)), represents a ~~significant~~ departure from the traditional TDCR actuation paradigm. This ~~novel~~ approach enables a higher

compactness in hollow manipulators and introduces a ~~novel~~ measure of continuum robot miniaturization. " →

"The concept of coupling a prismatic (push-pull) and a rotational (twist) joint at the same tendon anchoring point, extending beyond the separated radial and oblique muscle forms in biological systems (23, 24, 31), departs from the conventional TDCR actuation paradigm and allows greater compactness in hollow manipulators while providing a practical metric for continuum robot miniaturization." (More neutral)

- "For ease of comparison, **Table 1** summarizes recent reports of soft and continuum robots that exhibit twistable-body motion. While most studies exploit body twist either passively or through pre-programmed routines, the PPT tendon joint ~~mechanism provides a novel means to harness twisting via~~ enables twisting through dynamic, runtime configuration; ... " (More neutral)

- "These previously unreported designs and findings lay the foundation for exploring ~~new frontiers~~ in miniature tendon-driven continuum robots, offering more life-like and versatile motion, as well as enhanced manipulability." →

  "These previously unreported designs and findings provide a foundation for further development of miniature tendon-driven continuum robots, enabling more lifelike, versatile movements and improved manipulability." (Weaker tones)

Methods

- "Due to the eccentric placement of the tendon, the robot twist (about the tendon axis) is inherently non-axial (about the local notch frame), which can be ~~extremely~~ apparent on longer robots." →

  "Due to the eccentric placement of the tendon, the robot twist is inherently non-axial, and this effect becomes more pronounced in longer robots." (More neutral)
