# Supplementary material for: Single twistable tendon-driven continuum robots
Source: Nat Commun. 2026 Jul 4;17:8322. doi: 10.1038/s41467-026-74225-3 (PMC13469136; doi:10.1038/s41467-026-74225-3)
Supplement: Supplementary file 3 [file 41467_2026_74225_MOESM1_ESM.pdf]

Supplementary Materials for

Single twistable tendon-driven continuum robots

Jiewen Lai†, Yanjun Liu†, Tian-Ao Ren, Yan Ma, Tao Zhang,
Jeremy Yuen Chun Teoh, Mark R. Cutkosky, Hongliang Ren*

*Corresponding author. Email: hlren@ee.cuhk.edu.hk

†These authors contributed equally to this work.

This PDF file includes:

Supplementary Information

1. ID/OD and TCD/OD relationship in Fig. 2B
2. Geometry for Eccentric Notched Tube
3. Manipulability Index
4. Data Processing
5. Analysis for Tip Lateral Force Exertion under Different Modes of Motion (Supplementary for Fig. 5E)
6. Parameter Calibration
7. Four-story Tower
8. Dual Continua-based Gripper

Figures S1 to S11

Tables S1 to S5

Other Supplementary Materials for this manuscript:

Movies S1 to S15

Supplementary Information

1. ID/OD and TCD/OD relationship in Fig. 2B

In traditional TDCRs with three concentrically distributed tendon channels and an inner working channel, allowing for maximum TW widths to ensure balanced omnidirectional motion and robot durability, the OD can be calculated as the sum of four TW widths, the inner working channel diameter, and two tendon channel diameters (Fig. 2A(i)), as follows:

$$\mathrm{OD_{conc}} = 4\mathrm{TW} + \mathrm{ID} + 2\mathrm{TCD}. \tag{S1}$$

For the eccentric cases (2A(ii–iii)), the relationship becomes:

$$\mathrm{OD_{ecc}} = 3\mathrm{TW} + \mathrm{ID} + \mathrm{TCD}. \tag{S2}$$

The above equations can be rewritten as:

$$\frac{\mathrm{ID}}{\mathrm{OD_{conc}}} = 1 - 2\frac{\mathrm{TCD}}{\mathrm{OD_{conc}}} - 4\eta_{\mathrm{conc}} \tag{S3}$$

$$\frac{\mathrm{ID}}{\mathrm{OD_{ecc}}} = 1 - \frac{\mathrm{TCD}}{\mathrm{OD_{ecc}}} - 3\eta_{\mathrm{ecc}} \tag{S4}$$

where $\eta = \mathrm{TW/OD}$, $\mathrm{TCD/OD} \in (0, 0.4)$, $\mathrm{ID/OD} \in (0, 1)$. In Fig. 2B, the dashed lines are given by Eq. (S3), while the solid lines are given by Eq. (S4). The elevation on the y-axis represents the increase in the ID/OD ratio under the same TCD/OD condition.

2. Geometry for Eccentric Notched Tube

The shaded areas in Fig. 10B can be calculated as

$$A = 2\left(\frac{r^2\phi}{4} - \frac{1}{2}r\cos\left(\frac{\phi}{2}\right) r\sin\left(\frac{\phi}{2}\right)\right) \tag{S5a}$$

$$= \frac{r^2\left(\phi - \sin\left(\phi\right)\right)}{2}. \tag{S5b}$$

The centroids of the shaded regions (part of the sector shapes) can be represented in the local frame as

$$\bar{x} = 0, \tag{S6a}$$

$$\bar{y} = \frac{1}{A}\int_{r\cos\left(\frac{\phi}{2}\right)}^{r} \left(y\sqrt{r^2 - y^2}\right) \mathrm{d}y. \tag{S6b}$$

Given that $y = r\cos\widehat{\phi}$, so that Eq. (S6b) can be simplified as

$$\begin{aligned}
\bar{y} &= \frac{1}{A}\int_0^{\frac{\phi}{2}} \left(r\cos\widehat{\phi}\cdot r\sin\widehat{\phi}\cdot r\sin\widehat{\phi}\right)\mathrm{d}\widehat{\phi} && \text{(S7a)}\\
&= \frac{r^3}{A}\int_0^{\frac{\phi}{2}} \left(\cos\widehat{\phi}\cdot\sin^2\widehat{\phi}\right)\mathrm{d}\widehat{\phi} && \text{(S7b)}\\
&= \frac{r^3}{A}\int_0^{\frac{\phi}{2}} \sin^2\widehat{\phi}\,\mathrm{d}\left(\sin\widehat{\phi}\right) && \text{(S7c)}\\
&= \frac{r^3}{3A}\sin^3\left(\frac{\phi}{2}\right). && \text{(S7d)}
\end{aligned}$$

Note that the subscripts o and i are not used here, as the above calculation conforms to both cases.

3. Manipulability Index

This section introduces the manipulability of our continuum robots. The Jacobian matrix $\mathbf{J}$, which maps the joint velocities $\dot{\mathbf{q}} \in \mathbb{R}^3$ to the distal end velocities $\dot{\mathbf{x}} \in \mathbb{R}^6$ can be computed numerically by

$$\mathbf{J} = \frac{\Delta\mathbf{x}}{\Delta\mathbf{q}} = \begin{bmatrix} \dfrac{f(\mathbf{q}+\Delta q\,\mathbf{e}_1) - f(\mathbf{q}-\Delta q\,\mathbf{e}_1)}{\Delta q} \\ \dfrac{f(\mathbf{q}+\Delta q\,\mathbf{e}_2) - f(\mathbf{q}-\Delta q\,\mathbf{e}_2)}{\Delta q} \\ \dfrac{f(\mathbf{q}+\Delta q\,\mathbf{e}_3) - f(\mathbf{q}-\Delta q\,\mathbf{e}_3)}{\Delta q} \end{bmatrix}, \tag{S8}$$

where $f(\cdot)$ denotes the kinematics function $\mathbf{x} = f(\mathbf{q})$, $\mathbf{e}_i$ is the i-th unit vector of the canonical basis of the joint space, and Δq denotes a small perturbation for numeric computation—which is, in our case, $\Delta q = 10^{-6}$. Then the manipulability index μ can be computed based on its definition (*49*):

$$\mu = \sqrt{\det\left(\mathbf{J}\mathbf{J}^\top\right)}. \tag{S9}$$

Since the prismatic joint does not contribute to the angular velocity, only positional manipulability is presented in this paper, i.e., the first rows of the Jacobian are used for computing the manipulability index.

4. Data Processing

Note: The notation used in this section is independent from that of the main article.

Force. Force measurements $F[n]$, acquired during the quasi-static tendon loading, exhibited a series of step responses (violet curves in Fig. 5E). For analysis, we employed a multi-stage signal

processing pipeline implemented in MATLAB R2022a (academic use, MathWorks). Raw discrete-time signals $F[n]$ were initially preprocessed using a zero-phase fourth-order Butterworth low-pass filter with a 50 Hz cutoff frequency, yielding the filtered output $\tilde{F}[n]$ (cyan curves in Fig. 5E).

To further identify spike and settling windows, the velocity profile of the filtered signal was first derived as $\dot{\tilde{F}}[n] = \tilde{F}[n] - \tilde{F}[n-1]$. Subsequent convolution with a finite impulse response (FIR) moving average kernel $K[m]$ produced the smoothed detection signal $\mathcal{F}[n]$ as:

$$K[m] = \frac{1}{s}, \quad m = \{0, 1, \ldots, s-1\}, \tag{S10}$$

$$\mathcal{F}[n] = \left(\dot{\tilde{F}} * K\right)[n] = \frac{1}{s}\sum_{m=0}^{s-1} \dot{\tilde{F}}[n-m], \tag{S11}$$

where $s = 15$ denotes the kernel length. Spike windows $T_k^p \in [n_k^{ps}, n_k^{pe}]$ were identified as temporal intervals satisfying $\mathcal{F}[n] > \mathcal{F}_{th}$ (threshold $\mathcal{F}_{th} = 0.2$), discrete loading events indexed by k.

Settling windows $T_k^q \in [n_k^{qs}, n_k^{qe}]$ were algorithmically determined through relative temporal trimming with respect to neighboring spike initiation points:

$$\Delta T_k = n_{k+1}^{ps} - n_k^{pe}, \tag{S12}$$

$$n_k^{qs} = n_k^{pe} + \lfloor \alpha \Delta T_k \rfloor, \tag{S13}$$

$$n_k^{qe} = n_{k+1}^{ps} - \lceil \beta \Delta T_k \rceil, \tag{S14}$$

where empirical scaling factors $\alpha = 0.25$ and $\beta = 0.15$ ensured exclusion of transient regions adjacent to spike boundaries. The steady-state force value $\hat{F}_k$ for each event was computed as the temporal average:

$$\hat{F}_k = \frac{1}{n_k^{qe} - n_k^{qs} + 1} \sum_{m=n_k^{qs}}^{n_k^{qe}} \tilde{F}[m]. \tag{S15}$$

Optical posture. To analyze the motion of the robot's distal end, the position vector $\mathbf{P}$ and rotation matrix $\mathbf{R}$ are expressed using the homogeneous transformation matrix:

$$\mathbf{T} = \begin{bmatrix} \mathbf{R} & \mathbf{P} \\ \mathbf{0} & 1 \end{bmatrix} \in \mathbb{R}^{4\times4}.$$

Five relevant frames are defined as below to describe optical transform, as shown in Fig. S4C–D:

- $\{C\}$ – Camera frame: Origin at the midpoint between camera sensors;

- $\{B\}$ – Base frame: Attached to the first segment's cross section (origin and axes defined in Fig. 10);
- $\{E\}$ – Distal end frame: Attached to the final segment's cross section (origin and axes defined in Fig. 10);
- $\{S\}$ – Sensed marker frame: Origin at marker vertex V_2 with x-axis pointing to V_3, $x-y$ plane defined by $[V_1, V_2, V_3]$;
- $\{M\}$ – Designed marker frame: Origin at marker arm intersection, $x-y$ plane perpendicular to the extension.

The base frame $\{B\}$ remained fixed throughout experiments, while sensed data was acquired relative to $\{S\}$. This establishes raw pose data as $\mathbf{T}_S^C$ and target data as $\mathbf{T}_E^B$. Assuming coincident origins for $\{M\}$ and $\{E\}$, their orientation difference simplifies to a z-axis rotation:

$$\mathbf{T}_M^E = \begin{bmatrix} \mathbf{R}_M^E & \mathbf{0} \\ \mathbf{0} & 1 \end{bmatrix}, \tag{S16}$$

where

$$\mathbf{R}_M^E = \begin{bmatrix} \cos(\theta_m) & -\sin(\theta_m) & 0 \\ \sin(\theta_m) & \cos(\theta_m) & 0 \\ 0 & 0 & 1 \end{bmatrix} \tag{S17}$$

and θ_m was manually calibrated before experiments. The geometric transformation between marker frames is defined as:

$$\mathbf{T}_S^M = \begin{bmatrix} \mathbf{X} & \mathbf{Y} & \mathbf{Z} & \mathbf{OV_2} \\ 0 & 0 & 0 & 1 \end{bmatrix}, \tag{S18}$$

where basis vectors derive from marker geometry in Fig. S4:

$$\mathbf{X} = \frac{\mathbf{V_2V_3}}{\|\mathbf{V_2V_3}\|}, \quad \mathbf{Z} = \frac{\mathbf{V_2V_3} \times \mathbf{V_2V_1}}{\|\mathbf{V_2V_3} \times \mathbf{V_2V_1}\|}, \quad \mathbf{Y} = \frac{\mathbf{Z} \times \mathbf{X}}{\|\mathbf{Z} \times \mathbf{X}\|}, \tag{S19}$$

$$\mathbf{OV_2} = [-OV_2 \cos\alpha \sin\beta \quad OV_2 \cos\alpha \cos\beta \quad OV_2 \sin\alpha]^\top, \tag{S20}$$

$$\mathbf{V_2V_1} = [OV_2 \cos\alpha \sin\beta \quad (OV_1 - OV_2 \cos\beta) \cos\alpha \quad (OV_1 - OV_2) \sin\alpha]^\top, \tag{S21}$$

$$\mathbf{V_2V_3} = [(OV_3 + OV_2) \cos\alpha \sin\beta \quad (OV_3 - OV_2) \cos\alpha \cos\beta \quad (OV_3 - OV_2) \sin\alpha]^\top, \tag{S22}$$

with geometric parameters $\alpha = 30°$ (arm tilt angle) and $\beta = 120°$ (inter-arm angle).

Our experimental methodology assumed precise alignment between the robot's tip position measurements and its theoretical kinematic model in the rest state. Therefore, the initial base-to-end transformation $\mathbf{T}_E^B[0]$ could be derived from the robot's notch parameters. The initial reference posture $\mathbf{T}_S^C[0]$ was calculated through temporal averaging of the first ten acquired data points. The posture estimation framework at arbitrary timestep k follows these transformations:

$$\mathbf{T}_B^C[0] = \mathbf{T}_S^C[0] \cdot \left(\mathbf{T}_M^E \cdot \mathbf{T}_S^M\right)^{-1} \cdot \mathbf{T}_B^E[0], \tag{S23}$$

$$\mathbf{T}_B^C[k] = \mathbf{T}_B^C[0], \tag{S24}$$

$$\mathbf{T}_E^B[k] = \mathbf{T}_B^C[k]^{-1} \cdot \mathbf{T}_S^C[k] \cdot \left(\mathbf{T}_M^E \cdot \mathbf{T}_S^M\right)^{-1}, \tag{S25}$$

The verification experiment (Fig. 3) quantified the discrepancy between the model and experimental data for a specific joint configuration, with the terminal state $\mathbf{T}_S^C[k]$ calculated as the average of the last ten data points. In contrast, the hysteresis characterization experiment (Fig. 5F) analyzed the full motion trajectory using batched processing, where k represents the batched temporal indices for vectorized computation.

5. Analysis for Tip Lateral Force Exertion under Different Modes of Motion (Supplementary for Fig. 5E)

The evaluation of tip lateral force under different motion modes, as presented in Fig. 5E, was initially restricted to the direction perpendicular to the bending plane. While twisting enables robot–environment interactions across multiple surfaces—one of the key advantages of this motion—we extended the analysis to include forces measured on two additional tilted planes. Using the same experimental configuration as in Fig. 5E, the supplementary measurements shown in Fig. S6 provide an ablation analysis of how exerted forces are retained beyond the bending-perpendicular surface. Specifically, the force sensor was positioned at tilted angles of ±30° relative to the bending-perpendicular plane, as illustrated in Fig. S6B,D. The results reaffirm that twisting induces a reduction of 20–30% in output force compared with bending-only scenarios. Nevertheless, tip forces remain effective in interacting with the environment, highlighting the omnidirectional manipulability of TDCRs achieved simply by incorporating an additional twisting motion on the actuation tendon.

6. Parameter Calibration

The proposed kinematic model incorporates four empirically determined hyperparameters: the bending gains $\xi_{\pm}$ characterizing actuation asymmetry in bilateral bending, and the twist-induced bending coefficients $[K_x, K_y]$ governing coupled deformations along the x- and y-axes. The former ones quantify the combined effects of tendon transmission, adhesive bonding, frictional losses, and material nonlinearity during push-pull actuation. Although positive and negative bending constitute a single kinematic DOF, their mechanical responses diverge due to distinct notch closure versus opening dynamics, which necessitates independent calibration of ξ_+ and ξ_- as scalar variables. In contrast, K_x and K_y are inherently coupled through the robot's geometric length and internal stress distribution, and are thus treated as a vector in the optimization process.

To identify these parameters, a hierarchical grid search comprising two stages (Fig. S7A) is proposed: (1) optimization of the bending gains ξ_+ and ξ_-; (2) optimization of the twist-induced coefficients $[K_x, K_y]$ conditioned on the values from stage (1). In principle, $\xi_{\pm}$ could be calibrated directly from experimental data, assuming pre-characterized material properties E_s and E_t. However, in practice, E_s varies due to fabrication tolerances and environmental factors. To reduce sensitivity, we normalize $\xi_+ = 1$ and calibrate an effective E_s using data from positive bending trials, where tendon actuation occurs on the concave side and notch closure yields more predictable shape. This establishes a reference state for subsequent calibration of ξ_- and $[K_x, K_y]$.

The grid search is implemented with ranges determined by physical constraints and preliminary experiments. In stage (1), ξ_- is searched over $[0, 1]$ with 100 uniformly spaced candidates, using approximately 30 experimental trials comprising 3 distinct configurations with roughly 10 repetitions each (details in Fig. S5). The bounded search range reflects the observation that negative bending performance is compromised relative to the opposite situation due to variations in tendon stiffness during push-pull actuation. In stage (2), the twist-induced coefficients are searched over $[0, 0.15]$ for K_x and $[0, 0.05]$ for K_y, yielding a 50×50 candidate grid. These empirically determined ranges are validated using approximately 120 trials spanning 6 twisting and 6 coupled twist-bending configurations, each with roughly 10 repetitions.

The optimization criterion adopts a geodesic distance metric between model predictions and experimental measurements, which is geometrically appropriate for quantifying pose discrepancies

on the SE(3) manifold. For a parameter set $\boldsymbol{\theta}$ (either ξ_- or $[K_x, K_y]$), the optimal values are determined by minimizing the mean geodesic distance over the trials:

$$\boldsymbol{\theta}^* = \arg\min_{\boldsymbol{\theta}} \frac{1}{N} \sum_{i=1}^{N} \rho(\mathbf{T}_{e,i}, \mathbf{T}_{m,i}(\mathbf{q}_i, \boldsymbol{\theta})), \tag{S26}$$

where $\mathbf{T}_{m,i}, \mathbf{T}_{e,i} \in \mathrm{SE}(3)$ denote the model-predicted and experimentally measured poses of the robot tip for the i-th data point, respectively; $\mathbf{q}_i$ represents the corresponding actuator inputs; and N is the number of data points. Each pose $\mathbf{T}$ comprises a rotation matrix $\mathbf{R} \in \mathrm{SO}(3)$ and position vector $\mathbf{P} \in \mathbb{R}^3$. The distance ρ combines rotational and translational discrepancies as:

$$\rho(\mathbf{T}_e, \mathbf{T}_m) = \sqrt{\tilde{\theta}(\mathbf{P}_e, \mathbf{P}_m)^2 + \tilde{P}(\mathbf{P}_e, \mathbf{P}_m)^2}, \tag{S27}$$

$$\tilde{\theta}(\mathbf{R}_e, \mathbf{R}_m) = \arccos\left(\frac{\mathrm{Tr}(\mathbf{R}_e^T \mathbf{R}_m) - 1}{2}\right), \quad \tilde{P}(\mathbf{P}_e, \mathbf{P}_m) = \|\mathbf{P}_e - \mathbf{P}_m\|. \tag{S28}$$

In stage (1), negative bending data are used to calibrate ξ_-, yielding a convex error profile (Fig. S7B). In stage (2), $[K_x, K_y]$ are calibrated using data from pure twisting and combined twist-bending configurations, resulting in a convex heatmap over the parameter space (Fig. S7C). These profiles confirm the convexity of the kinematic model with respect to the hyperparameters, suggesting opportunities for advanced techniques such as adaptive parameter estimation and model-based control. For Robot ①, the calibration yielded $\xi_- = 0.56$, $K_x = 0.0735$, and $K_y = 0.0020$, while Robot ② exhibited $\xi_- = 0.68$, $K_x = 0.0306$, and $K_y = 0.0061$. The consistently small magnitudes of K_y across both instances corroborate the minor influence of coupling-induced bending, as this coefficient characterizes deformations aligned with the primary actuation direction.

The calibrated coefficients are inherently type-specific, influenced by design factors (e.g., length, notch density, wall thickness), material properties, and fabrication inconsistencies. Thus, we suggest executing this calibration protocol for each robot instance. Nonetheless, the hierarchical optimization framework is broadly applicable to diverse continuum robot architectures, albeit with variant parameter outcomes.

7. Four-story Tower

The tower shown in Fig. 6 consists of four evenly spaced disks connected by a central pillar, supported by a base disk (Fig. S9). Each disk features a 7 mm-diameter hole, offset by 45° relative

to the hole in the adjacent disks. This staggered configuration creates a tortuous path designed to evaluate the robot's motion capabilities. The structure yields a 28.34% overlap area in the top view and a 3.11 mm overlap projection length in the side view.

A smiling face marker was placed on the base disk and aligned with the final hole to evaluate the robot's ability to adjust its orientation. The robot's distal end was initially positioned just above the first disk, resulting in a total vertical displacement of 48 mm required to traverse the tower. Manual commands guided the robot through each hole in sequence until the marker became visible in the correct orientation through the embedded tip camera.

8. Dual Continua-based Gripper

The dual continua-based gripper features two soft robots mounted on a base fixture with two handles for manual manipulation. Each handle was threaded through the tendon via a 0.5 mm-diameter channel (see Fig. S11A–C) and positioned 30–40 mm from the fixture for easy manual access and steering. Adhesives were applied between the handles and the tendon path to ensure reliable transmission of manual control inputs. This design ensures that handle rotation generates pure tendon torque, while linear handle displacement, such as pulling and pushing, produces axial tendon forces. The fixture includes two parallel sockets on one side to secure the robots' bases and two thin chambers on the other side to guide the tendons smoothly (see Fig. S11D–F).

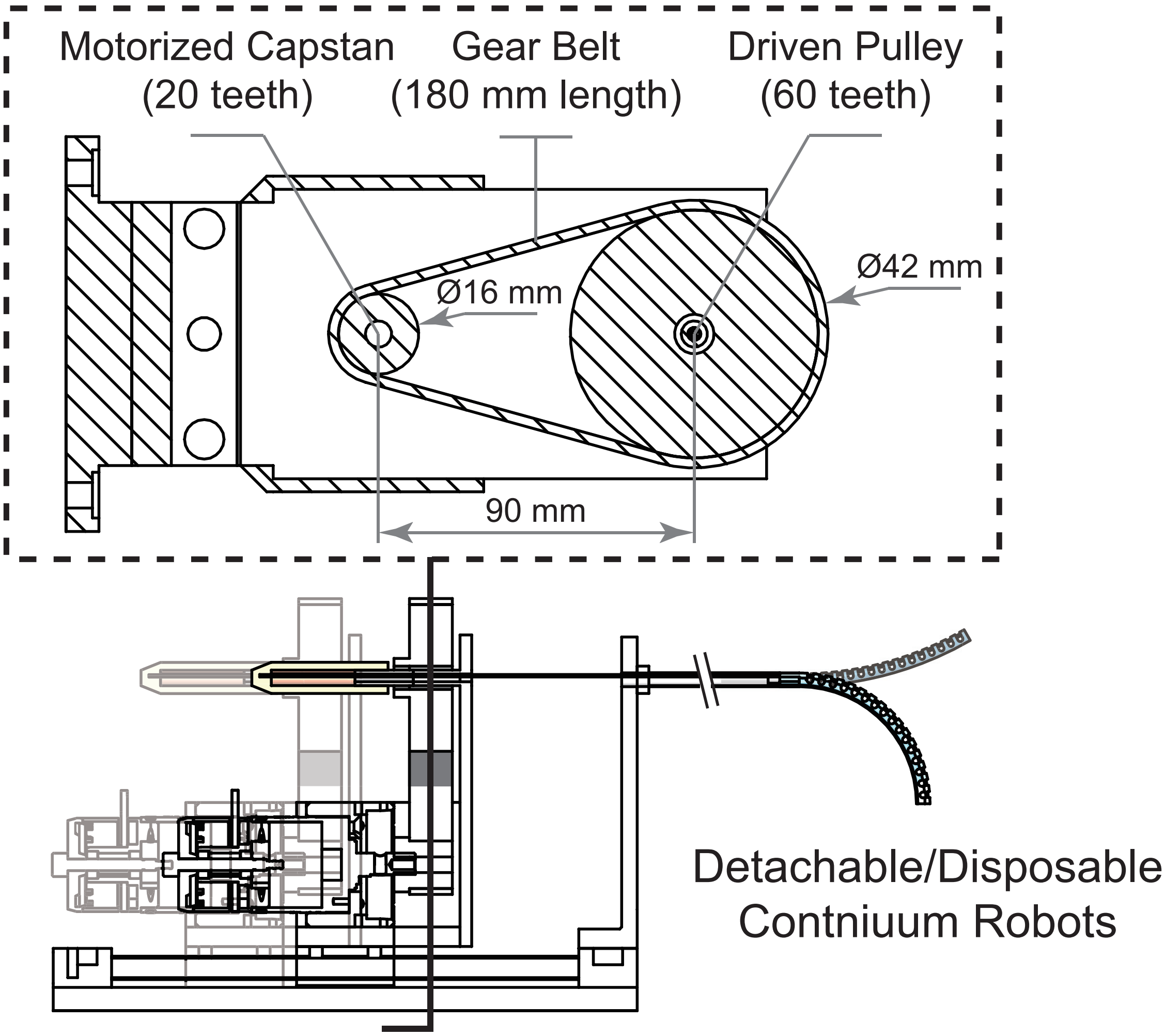

Figure S1: **Drawings of the rotation joint.** The prismatic joint and rotational joint constitute the PPT mechanism acting on the single tendon.

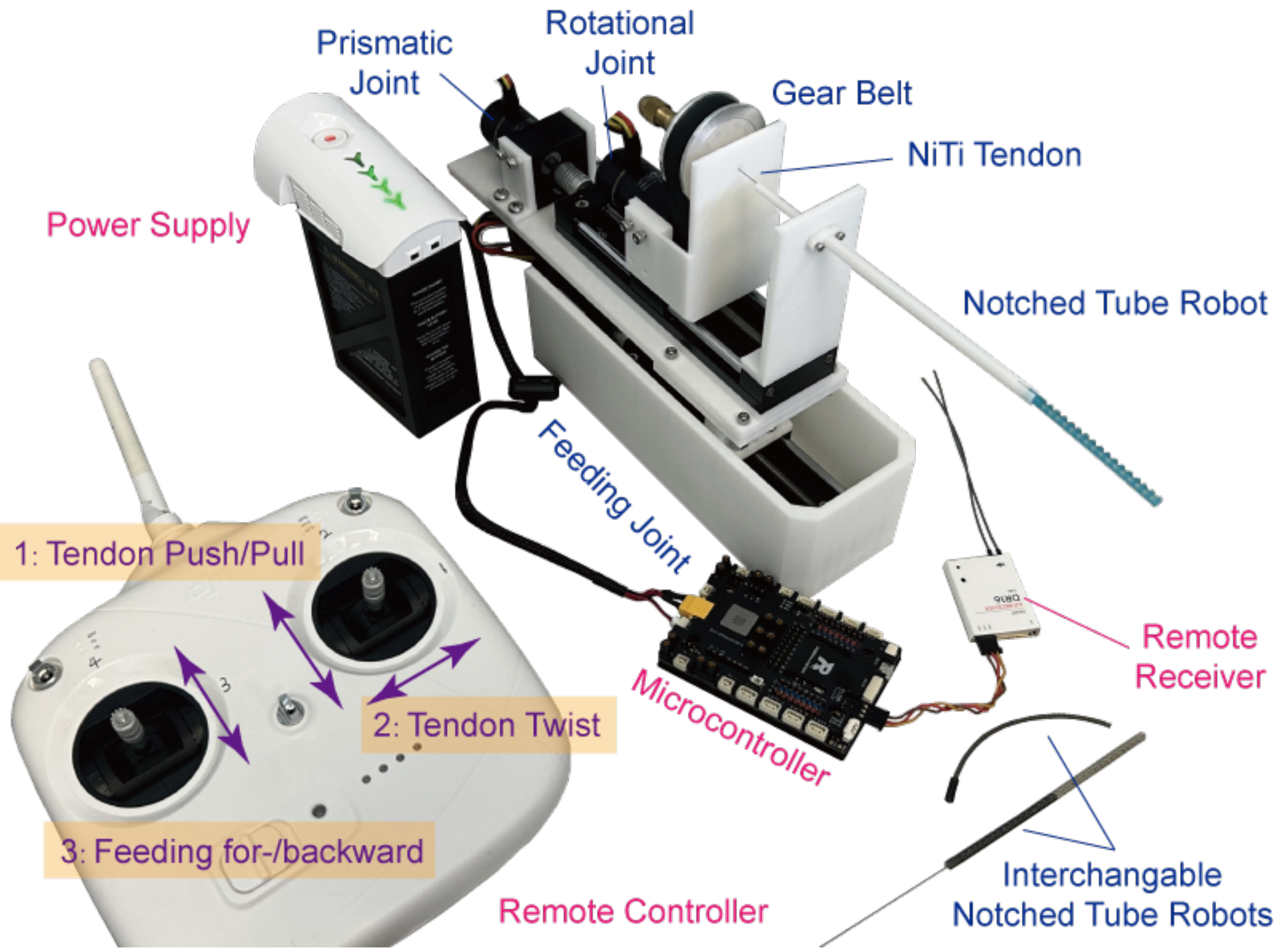

Figure S2: **Overview of the teleoperated single tendon-driven continuum robotic system.** The robot can be teleoperated via a remote controller or a computer (serial link connection). The notched tube TDCR can be conveniently detached for interchange.

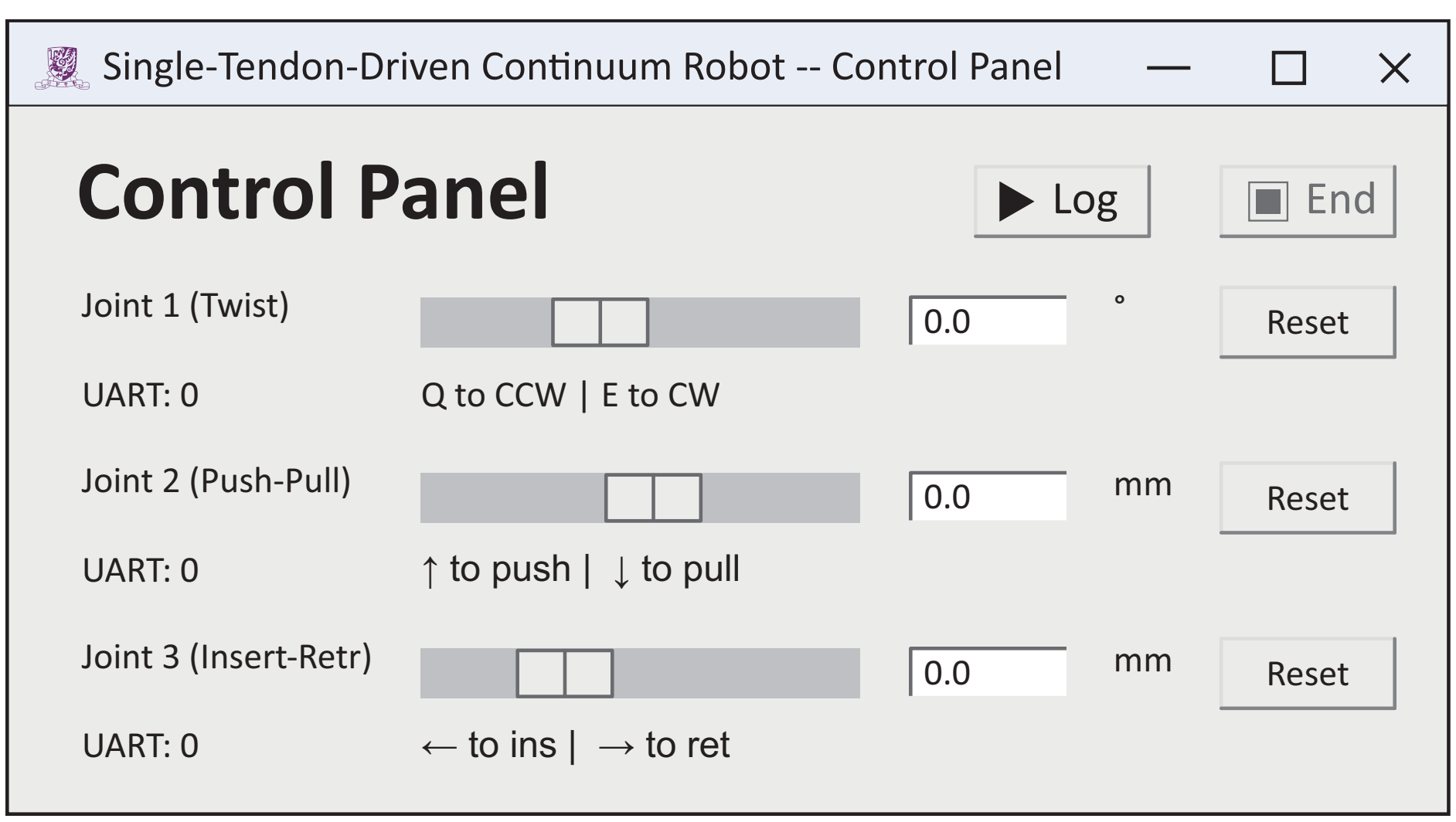

Figure S3: **Design of the graphical user interface (GUI) for robot teleoperation control panel.** The robot joints can be controlled using (1) slider bars, (2) numerical input, or (3) keyboard. Additionally, it features a data logging function to record temporal inputs.

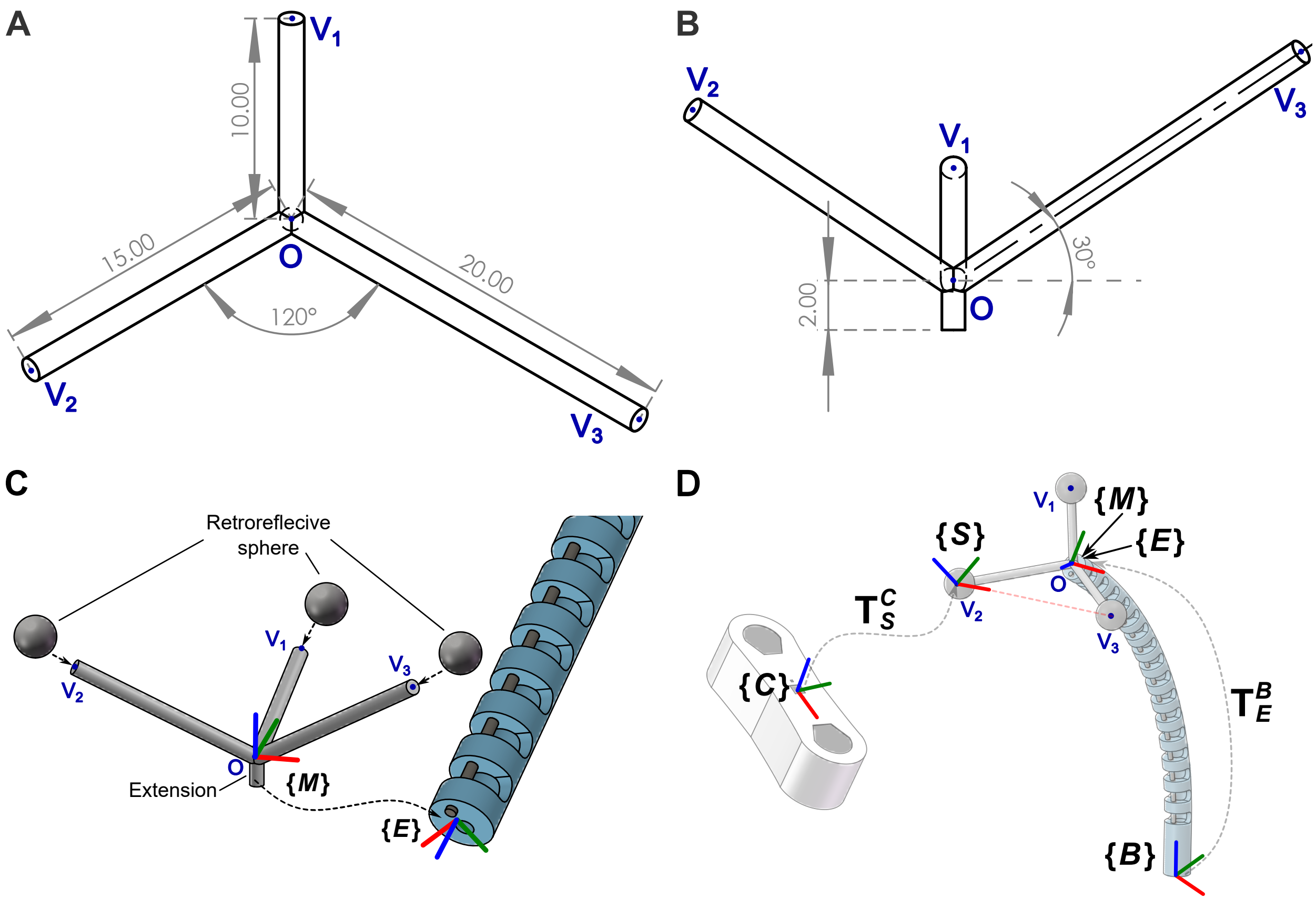

Figure S4: **Optical tracking system for robotic pose estimation.** (**A**) Top-view and (**B**) front-view drawings of the custom optical marker (units are in mm). (**C**) Assembly procedure of spheres, frame, and robot. (**D**) Coordinate system for posture data collection.

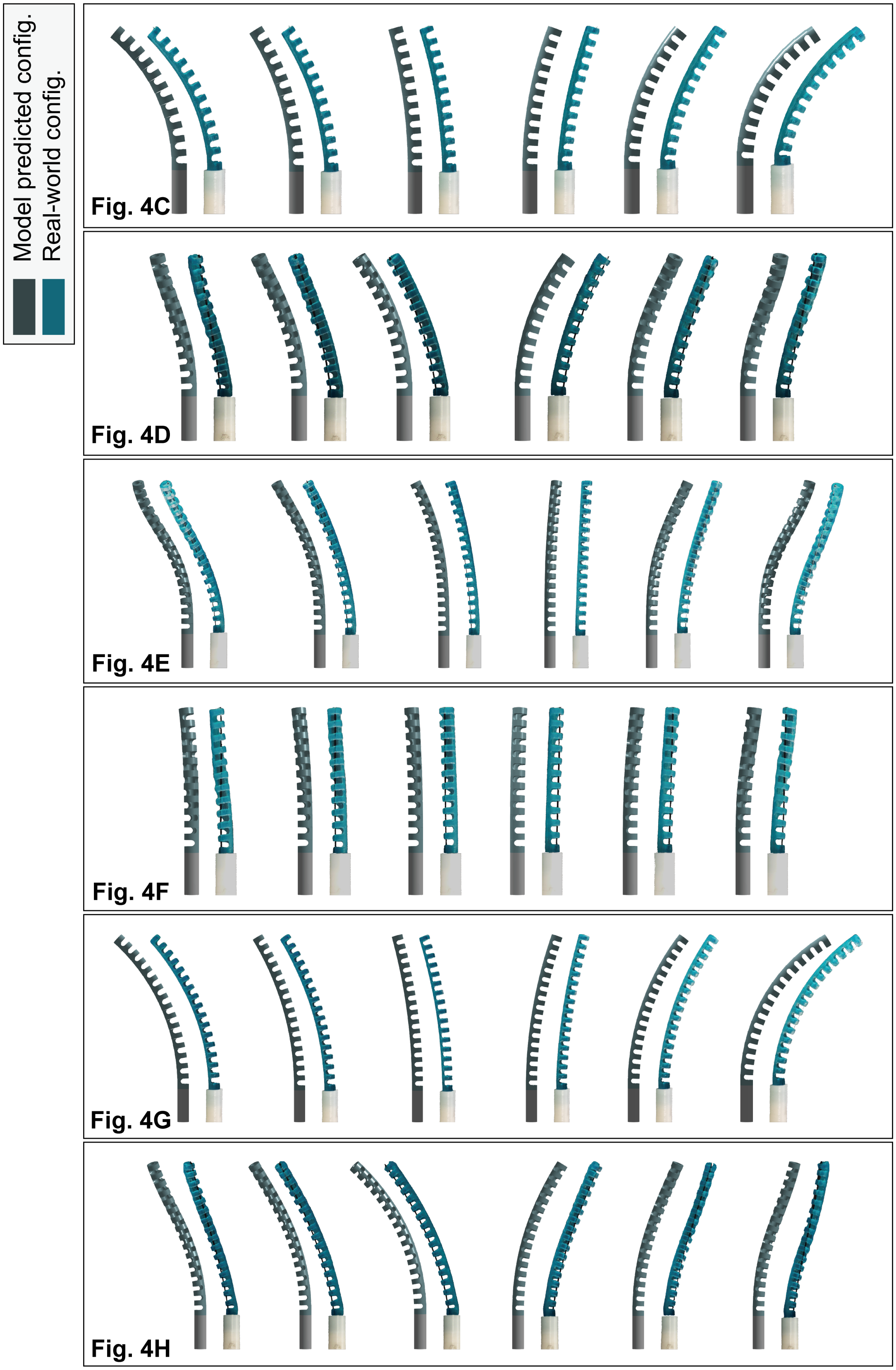

Figure S5: **Zoomed-in view of Fig. 3C–H**.

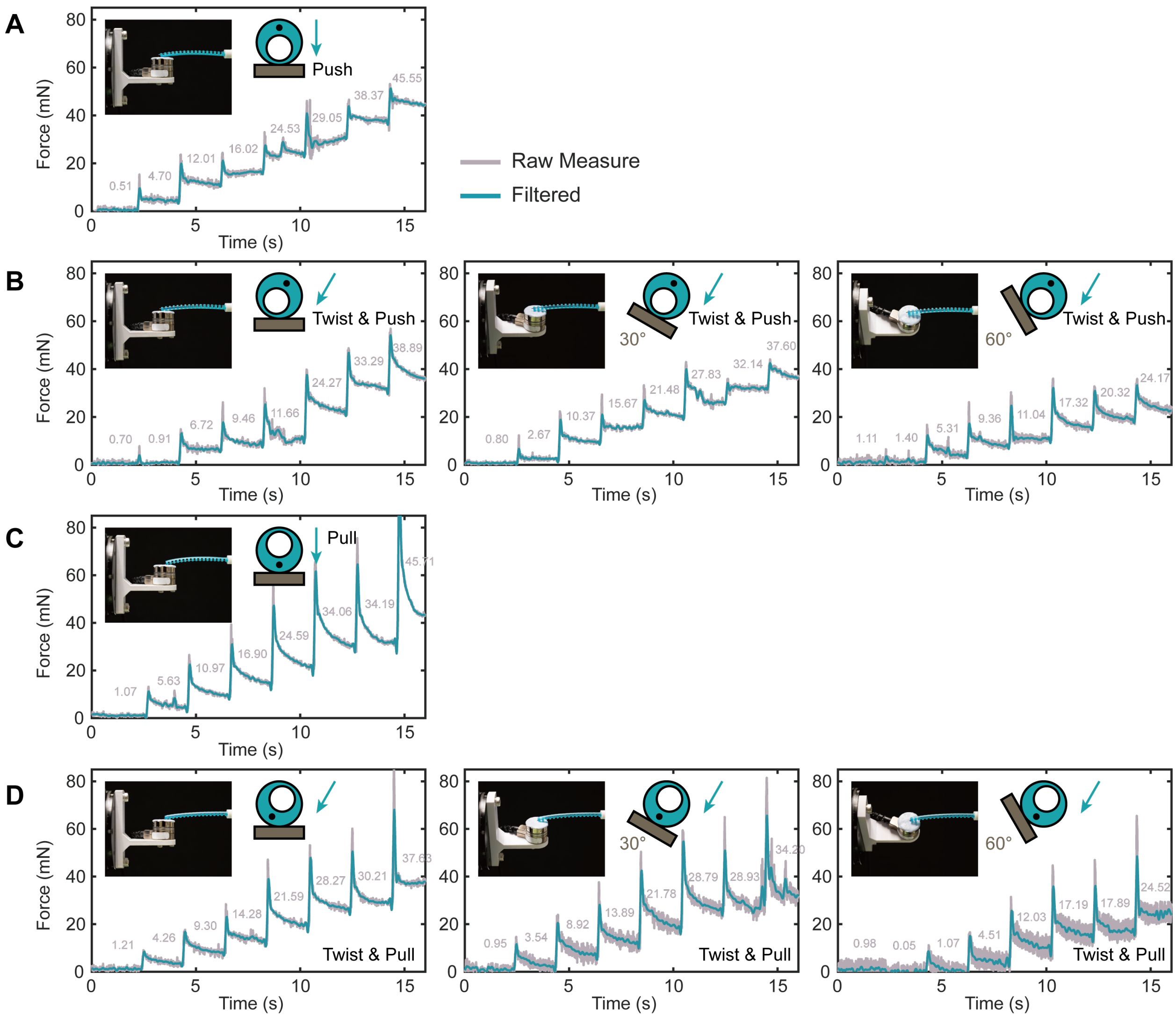

Figure S6: **Measurement on distal lateral force outputs involving robot's twisting motions on bending-perpendicular and non-bending-perpendicular planes.** **(A)** Robot bends through tendon pushing. This represents a repeated experiment corresponding to Fig. 5E(i). **(B)** Robot deforms through tendon pushing and twisting, with force sensor position on bending and non-bending planes. This represents an ablation experiment corresponding to Fig. 5E(ii). **(C)** Robot bends through tendon pulling. This represents a repeated experiment corresponding to Fig. 5E(iii). **(D)** Robot deforms through tendon pulling and twisting, with force sensor position on bending and non-bending planes. This represents an ablation experiment corresponding to Fig. 5E(iv).

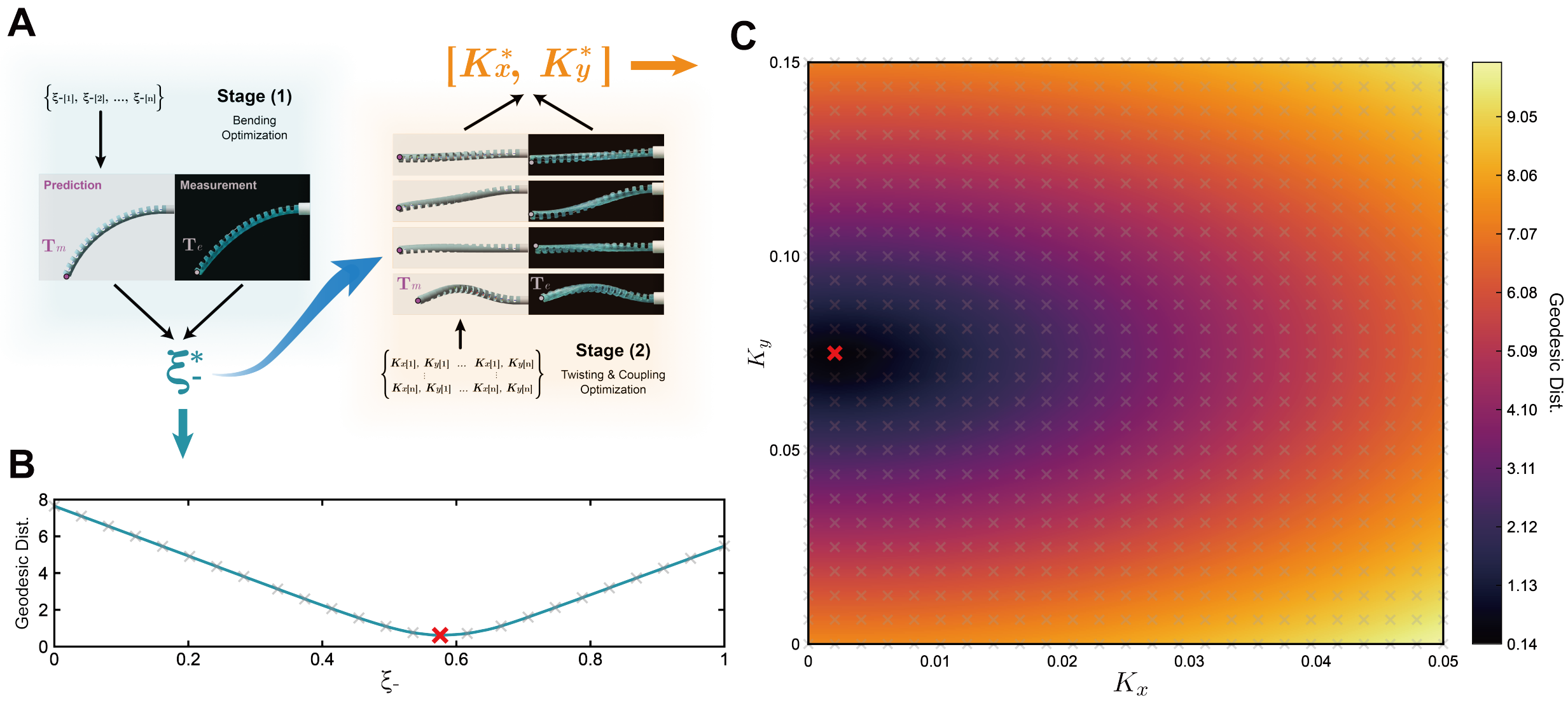

Figure S7: **Hierarchical parameter calibration.** (**A**) Schematic of the optimization workflow. Stage (1) involves fitting the negative bending gain ξ_- using data from pure bending trials; Stage (2) fits the twist-induced coupling coefficients K_x and K_y from twisting and coupled twist-bending trials. (**B**) Error profile as a function of ξ_- for Robot ①, showing a convex profile with a well-defined minimum (red cross). (**C**) Heatmap of the error surface across the parameter space spanned by $[K_x, K_y]$ for Robot ①, showing a convex basin highlighted by the global minimum (red cross).

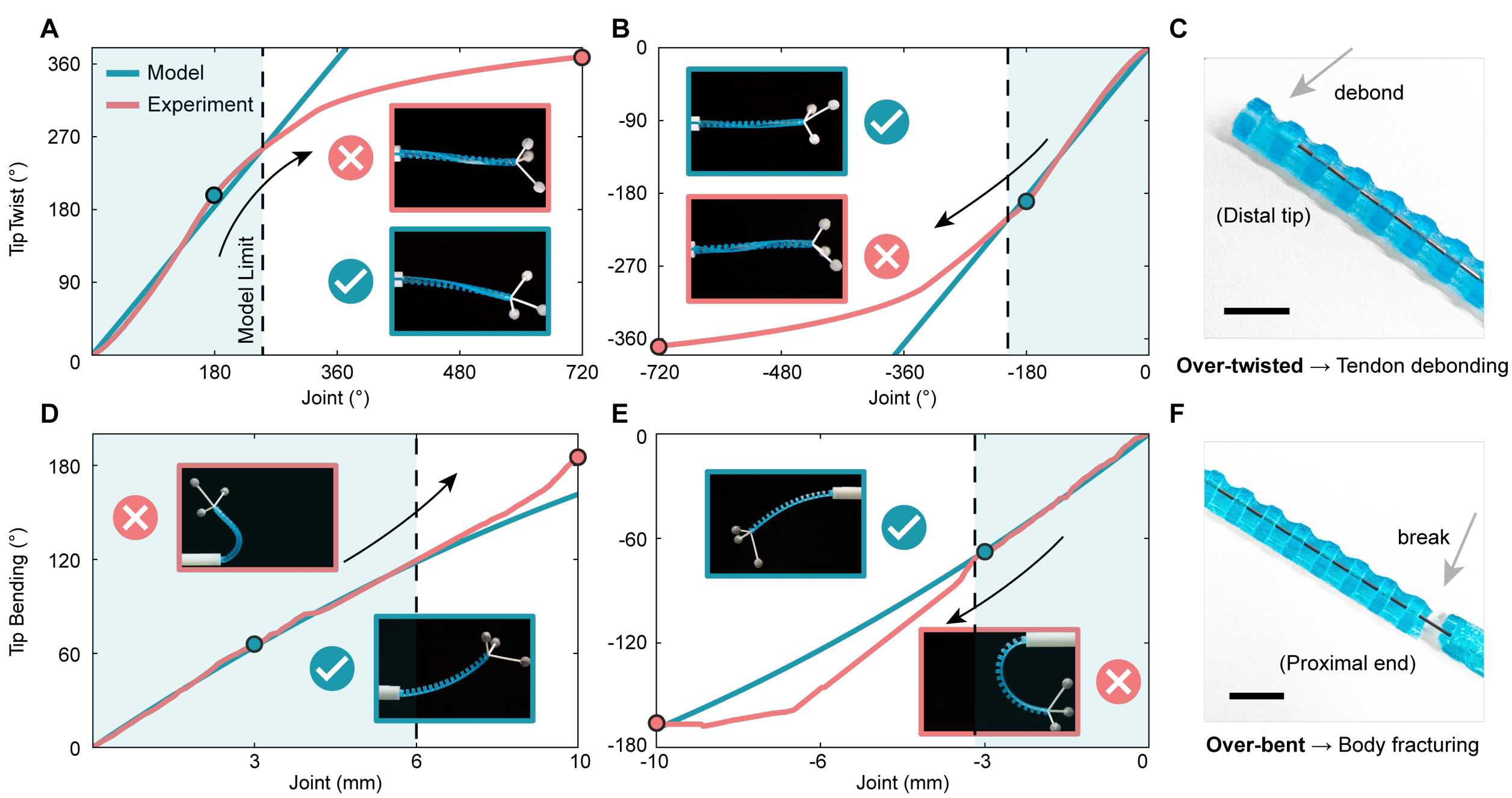

Figure S8: **The range of satisfactory model predictions (absolute tip bending or twisting error ≤ 3°) evaluated on Robot ②.** Experimental measurements indicate that **(A)** positive twisting reaches the predictable operational limit of 255°; **(B)** negative twisting reaches -209°; **(C)** excessive twisting may lead to debonding at the adhesive interface; **(D)** positive bending reaches the predictable operational limit of 119°; **(E)** negative bending reaches -70°; **(F)** prolonged or excessive bending may cause fracture of the soft body. These damages may occur after long-term use. (Scale bars: 5 mm).

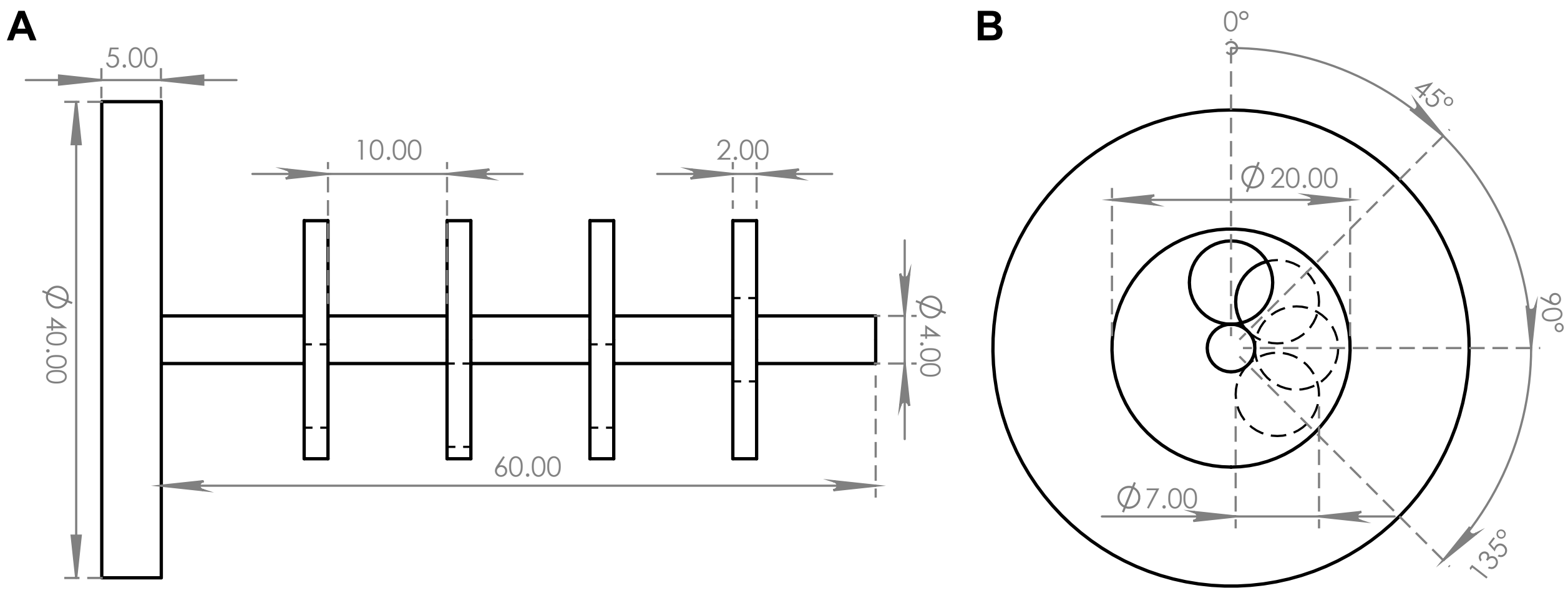

Figure S9: **Drawings of the four-story tower.** The units are in mm. (**A**) Front view (rotated 90°). (**B**) Top view.

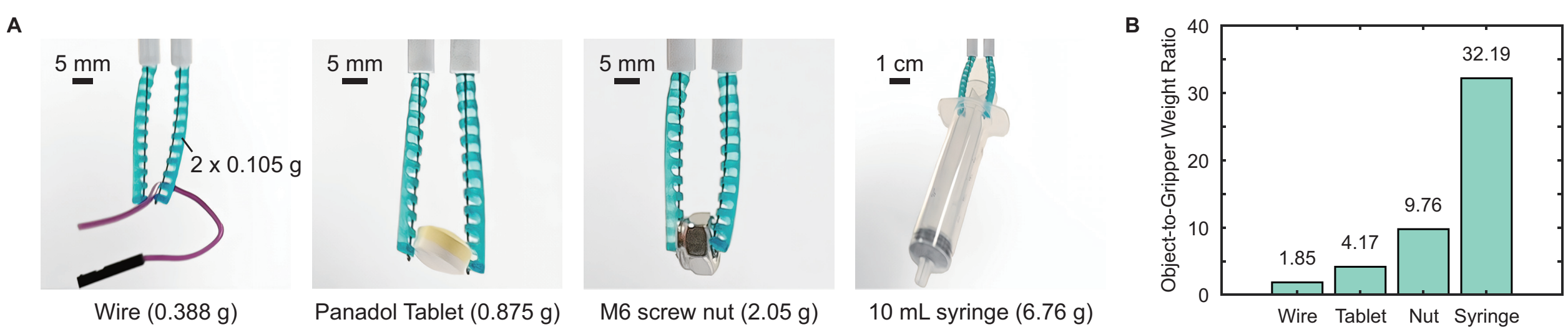

Figure S10: **Dual continua-based soft gripper grasping tiny, soft objects.** (**A**) The soft gripper is adaptive to objects of various shapes and weights, including a wire (0.388 grams), a Panadol tablet (0.875 grams), an M6 screw nut (2.05 grams), and a 10 mL syringe (6.76 grams), (**B**) correspond to 1.85 times, 4.17 times, 9.76 times, and 32.19 times the soft gripper's net weight, respectively.

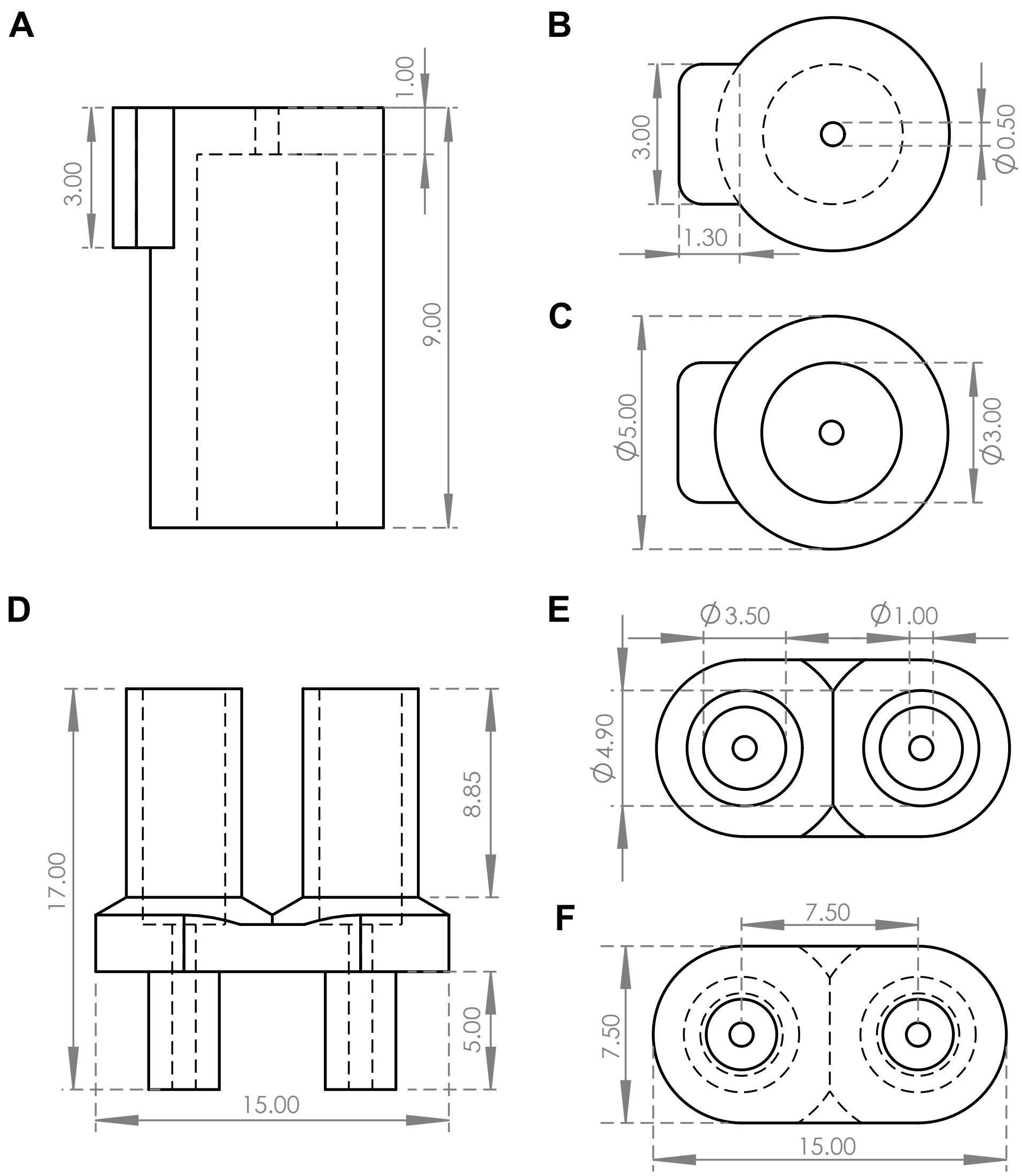

Figure S11: **Drawings of the gripper fixture and handle.** The units are in mm. (**A**)-(**C**) are the front, top, and bottom views of the handle. (**D**)-(**F**) are the front, top, and bottom views of the fixture.

Table S1: **Parameters for Robot ① shown in Fig. 1H**

**Param.**	**Value**	**Unit**	**Physical Meaning**
m	11	-	A notched tube TDCR with m notches
n	24	-	The robot has a total of n frames
k	-	-	The k-th frame, where $k = 0$ is the base frame
g	2.5	mm	Cutting depth of the notches
r_o	1.75	mm	Radius of the robot
r_i	1.0	mm	Radius of the inner working channel
r_t	0.3	mm	Radius of the tendon channel
d_t	1.04	mm	Distance of centroids between the robot and the tendon channel
d_w	0.44	mm	Offset of the inner working channel deviated from the robot centroid
h	1.5	mm	Length of the neutral line (within a single notch) on the bending plane
w	1.5	mm	Width of notched and uncut sections
L	35	mm	Length of the S-TDCR

Table S2: **Parameters for Robot ② shown in Fig. 1H**

**Param.**	**Value**	**Unit**	**Physical Meaning**
m	16	-	A notched tube TDCR with m notches
n	34	-	The robot has a total of n frames
k	-	-	The k-th frame, where $k = 0$ is the base frame
g	2.5	mm	Cutting depth of the notches
r_o	1.75	mm	Radius of the robot
r_i	1.0	mm	Radius of the inner working channel
r_t	0.3	mm	Radius of the tendon channel
d_t	1.04	mm	Distance between the centroids of the robot and the tendon channel
d_w	0.44	mm	Distance between the centroids of the robot and the working channel
h	1.5	mm	Length of the neutral line (within a single notch) on the bending plane
w	1.5	mm	Width of notched and uncut sections
L	50	mm	Length of the S-TDCR

Table S3: **Parameters for Robot ③ shown in Fig. 1H**

Param.	Value	Unit	Physical Meaning
m	24	-	A notched tube TDCR with m notches
n	66	-	The robot has a total of n frames
k	-	-	The k-th frame, where $k = 0$ is the base frame
g	1	mm	Cutting depth of the notches
r_o	1	mm	Radius of the robot
r_i	0.375	mm	Radius of the inner working channel
r_t	0.25	mm	Radius of the tendon channel
d_t	0.5	mm	Distance between the centroids of the robot and the tendon channel
d_w	0.25	mm	Distance between the centroids of the robot and the working channel
h	1	mm	Length of the neutral line (within a single notch) on the bending plane
w	1	mm	Width of notched and uncut sections
L	55	mm	Length of the S-TDCR

Table S4: **Parameters for Robot ④ shown in Fig. 1H**

Param.	Value	Unit	Physical Meaning
m	32	-	A notched tube TDCR with m notches
n	66	-	The robot has a total of n frames
k	-	-	The k-th frame, where $k = 0$ is the base frame
g	2.5	mm	Cutting depth of the notches
r_o	1.75	mm	Radius of the robot
r_i	1.0	mm	Radius of the inner working channel
r_t	0.3	mm	Radius of the tendon channel
d_t	1.04	mm	Distance between the centroids of the robot and the tendon channel
d_w	0.44	mm	Distance between the centroids of the robot and the working channel
h	1.5	mm	Length of the neutral line (within a single notch) on the bending plane
w	1.5	mm	Width of notched and uncut sections
L	98	mm	Length of the S-TDCR

Table S5: Comparative overview of continuum robots featuring twistable-body motion

Ref.	Actuation for Continuum	Mechanism for Body Twisting	3D Manipulability with Twisting Motion	Tip force Transmissibility ↑	Ease of Detachment
Oliver-Butler, *T-RO*, 2019 (*5*)	Tendon-driven	Converging tendon routing	Pre-programmed	High	✗
Russo, *T-MECH*, 2025 (*71*)	Tendon-driven	Helical tendon routing	Pre-programmed	High	✗
Peng, *Nat. Commun.*, 2025 (*40*)	Tendon-driven	Aerial base*	Passive	High	✗
Jones, *Nature*, 2021 (*17*)	Pneumatic-driven	Eccentric wall thickness	Pre-programmed	-	✓
Zhao, *T-MECH*, 2022 (*13*)	Pneumatic-driven	Rotatable base*	-	Medium	✗
Lee, *Nat. Commun.*, 2024 (*33*)	Pneumatic-driven	Mechanical constraint	Pre-programmed	Medium	✓
Zhao, *PNAS*, 2022 (*31*)	-	Twisted LCE ribbon	Surface rolling	-	-
Leanza, *Adv. Funct. Mater.*, 2024 (*23*)	LCE-driven	Helical LCE routing	Pre-programmed	Low	✗
Wu, *PNAS*, 2021 (*32*)	Magnetic-driven	Origami (Kresling unit)	Pre-programmed	Low	✓
Dreyfus, *Sci. Robot.*, 2024 (*18*)	Magnetic-driven	Helical protrusion	Steerable magnetic tip	-	✗
Tummers, *IJRR*, 2025 (*11*)	Push-pull concentric tubes	Helicoidal centroid lines	Dynamically configured	High	✓
**This work**	**Tendon-driven**	**PPT tendon joint**	**Dynamically configured**	**High**	✓

*Not an active continuum body twist in the strict sense.
