# Supplementary material for: Single twistable tendon-driven continuum robots
Source: Nat Commun. 2026 Jul 4;17:8322. doi: 10.1038/s41467-026-74225-3 (PMC13469136; doi:10.1038/s41467-026-74225-3)
Supplement: Supplementary file 4 [file 41467_2026_74225_MOESM2_ESM.pdf]

Description of Additional Supplementary Files

Supplementary Movie 1: Robot Motion Modalities (Lateral View) The robot, once installed, can perform bending and twisting motions by PPT mechanism. Also the animated version of Fig. 1C.

Supplementary Movie 2:RobotMotionModalities (Perspective View) The robot, once installed, can perform bending and twisting motions by PPT mechanism.

Supplementary Movie 3: Quick Robot Assembly & Detachment The single-tendon robot can be assembled and detached quickly and easily. Also the animated version of Fig. 2D.

Supplementary Movie 4: Navigating Through Tortuous Environments — Demonstration The robot navi gates through a complex four-story tower mimicking torturous environments before aligning to a marker at the tower bottom. Also, the animated version of Fig. 6A.

Supplementary Movie 5: Navigating Through Tortuous Environments — Comparison Comparison of the robot's performance when navigating through holes in the four-story tower with and without the twist mechanism.

Supplementary Movie 6: Dual Continua-based In-hand Manipulation The dual-arm gripper performs three types of in-hand manipulation: sliding, lifting, and rotation.

Supplementary Movie 7: Dual Continua-based Pick & Place The dual-arm gripper transports two non standard objects weighing 0.405 grams.

Supplementary Movie 8: Dual Continua-based Grasping The dual-arm gripper grasps various objects, in cluding a wire, tablet, screw nut, and syringe.

Supplementary Movie 9: Robotic Otoendoscopy Robotic transcanal endoscopy for eardrum inspection, fea turing collision avoidance and view adjustment through a twisting mechanism. Also, the animated version of Fig. 7C.

Supplementary Movie 10: EarwaxRemoval Earwaxremovalinanearcanalphantomusingascoopembedded at the robot's tip. Also, the animated version of Fig. 7F. S23

Supplementary Movie 11: Endoscopic Gastrostomy Endoscopic inspection of the mockup stomach body and pylorus for potential ulcers prior to and post-PEG. Also, the animated version of Fig. 8C.

Supplementary Movie 12: Ex Vivo Experiment on Porcine Heart Endoscopic examination of an ex vivo porcine heart. The robot initiates navigation within the pulmonary artery, traverses the pulmonary semilunar valve and achieves access to the right ventricular chamber. Also, the animated version of Fig. 8G.

Supplementary Movie 13: In Vivo Experiment on Porcine Bladder Third-person view of the robot demon strating its omnidirectional motion and tissue sampling capabilities using integrated tip forceps within a porcine bladder. Also, the animated version of Fig. 9D–E.

Supplementary Movie 14: Motion Gallery — Different Tendon–Robot Combination The choice of ten don–soft/continuum body combinations can lead to diverse morphological and kinematic behav iors.

Supplementary Movie 15: 3D Printing Process The 3D printing process of the continuum robot body using the Stratasys J826. Drops of photopolymer are jetted onto the build platform, then cured with UV lamps to bond layers
